# Experiences of trauma among persons living with psychosis in KwaZulu Natal, South Africa

**DOI:** 10.1371/journal.pmen.0000070

**Published:** 2024-10-03

**Authors:** Vuyokazi Ntlantsana, Maud B. Donda, Mihoko Maru, Nduku G. Wambua, Usha Chhagan, Saeeda Paruk, Bonginkosi Chiliza, Lauren C. Ng

**Affiliations:** 1 Department of Psychiatry, School of Clinical Medicine, College of Health Sciences, University of KwaZulu- Natal, Durban, South Africa; 2 Center for Innovation in Social Work & Health, School of Social Work, Boston University, Boston, Massachusetts, United States of America; 3 Mental Health Research, AHRI: Africa Health Research Institute, Durban, South Africa; 4 Psychology Department, University of California Los Angeles, Los Angeles, California, United States of America; University of Ghana, GHANA

## Abstract

Existing literature from high-resource settings suggests that exposure to traumatic life events is associated with increased risk for psychosis. Research on how people with psychosis in South Africa perceive and make sense of their past traumatic life events is lacking. This study aimed to understand the lived experiences of persons living with psychosis in KwaZulu-Natal (KZN), South Africa. The study explored how individuals living with psychosis describe and conceptualize their traumatic life experiences and how it impacted them. We used a qualitative narrative research study design. Individual in-depth interviews were conducted in English and/or isiZulu with 19 adult patients with early psychosis who were receiving treatment at provincial hospitals in the eThekwini district in KZN, South Africa (mean age = 28.7±7.6, mostly male (68%) and unemployed (74%)). We analysed transcribed data using interpretive phenomenological analysis. Participants’ descriptions of traumatic life experiences included parent-child relationship disruption during their formative years and other adverse events associated with being separated from their parental figures including residential instability, financial hardship, sexual and physical abuse, and peer and community violence. Participants also described the experience of psychosis as a traumatic event. These traumatic experiences led to sense of loss for some participants, while others described post-traumatic stress symptoms including reexperiencing their trauma memories through psychotic episodes. The study provides clinical insight on the experiences of trauma among people with psychosis and highlights the need for adopting a trauma-informed service model in early psychosis care in South Africa.

## Introduction

Psychotic disorders are associated with the highest burden of psychosocial disability worldwide [1]. Recent global epidemiological data show that the prevalence of schizophrenia in South Africa is 0.25%, affecting approximately 145,000 people [2, 3]. Individuals with psychotic disorders can experience long-term limitations in cognitive, social, and vocational functioning, among others [4].

Existing literature from high-resource/income nations suggests that exposure to traumatic life events is associated with increased risk for psychosis [5–8]. In South Africa, the association between trauma and psychosis is an extremely relevant area of further research given the country’s high reported rates of trauma. In a nationally representative sample of South Africans, 74% reported experiencing at least one traumatic event in their lifetime; higher rates (94%) have been reported among people with psychosis [9, 10] In a separate study, the odds of having experienced childhood trauma were 2.44 times higher for those with schizophrenia compared to matched controls without schizophrenia [11]. Furthermore, among 80 inpatients with schizophrenia, 4.3% met criteria for PTSD, though actual rates are likely to be under-reported [12, 13] History of traumatic experiences have also been found to be significantly associated with higher levels of both positive and negative symptoms of psychosis [14].

The high rates of trauma in South Africa can be attributed to historical, social and cultural issues [15, 16]. The history of apartheid characterised by racial oppression, marginalisation, and the displacement of communities, as well as mass violence due to the political repression and corruption during the post-apartheid era, has fostered intergenerational trauma. During apartheid, displaced communities were often separated from cultural systems and heritage, weakening social cohesion and community networks the once served as protective factors against trauma. Other contextual stressors, including poverty and other forms of social inequity, high crime rates, and unemployment have contributed to high levels of ongoing stress. Low social support combined with limited mental healthcare resources have increased the vulnerability of these communities to the impact of trauma, thereby increasing their risk for poor mental health.

Despite a growing body of evidence, the adoption of trauma-informed care within psychiatric practice in South Africa is still limited. Viewing the patient with a psychotic disorder through the lens of their trauma experience may allow providers to better meet their patient’s needs. More research is needed however, to develop appropriate techniques to address this gap in practice. To date, the authors are not aware of qualitative studies that have explored the experiences of trauma among South Africans who have a psychotic disorder. Specifically, how people living with psychosis in South Africa perceive and make sense of traumatic life events is an understudied area. This study aimed to understand the lived experiences of persons living with psychosis in KwaZulu-Natal, South Africa. The study explored how individuals living with psychosis describe and conceptualize their traumatic life experiences and how it impacted them. The following research questions guided the study: 1) What are the traumatic experiences of people with early psychosis? 2) What are the post-traumatic experiences? and 3) How did they make sense of the traumatic experiences and how it impacted them?

## Setting

The study was conducted at the psychiatric units of four hospitals in the eThekwini district in the KwaZulu-Natal Province, South Africa. The hospitals have both inpatient and outpatient facilities and medical care is provided by specialist psychiatrists, psychiatry residents, general medical doctors, and nursing personnel. eThekwini has a population of approximately 3.4 million inhabitants with a 64% literacy rate of grade seven and above, and high rates of crime and interpersonal violence [17, 18].

## Methods

### Ethics statement

The University of KwaZulu-Natal Biomedical Research Ethics Committee gave permission to conduct the study (BREC/00000089/2019). All participants were screened for capacity to consent by the treating clinician before referral and again by the principal investigator (VN). All participants provided written informed consent.

### Data collection

We collected data as part of a process towards culturally adapting an intervention for PTSD among patients with serious mental illness. The adapted intervention was to be piloted on patients in an existing cohort enrolled in a larger study on HIV among first-episode psychosis (FEP) patients [19]. We used a qualitative narrative research study design to explore the lived experiences of trauma among adult patients with early psychosis who were receiving treatment at any of the four hospitals.

### Study sample and recruitment

Patients were invited to participate in this study if they 1) had been diagnosed with a psychotic disorder within the last five years, 2) self-reported a history of exposure to at least one traumatic event in their lifetime, 3) were between ages 18–45 years, 4) had a level of education of grade 7 or higher, and 5) were able to give informed consent. Trauma histories were assessed by the researcher asking each participant whether they have had “difficult life experiences” or “traumatic events” in the past. Individuals who were 1) unable to give consent and/or 2) were actively experiencing psychotic symptoms and as a result assessed to be unwell by the treating physician at the time of recruitment were excluded from the study.

The principal investigator liaised with the treating physicians and screened clinical records of patients receiving in- or outpatient care to determine eligibility. A diagnosis of a psychotic disorder was initially made by the treating physician, using the DSM-5 criteria [20]. The principal investigator confirmed this diagnosis by reviewing the clinical records. A diagnosis of a psychotic disorder was confirmed if there was documentation of two or more of the following symptoms: delusions, hallucinations, disorganised speech, disorganised or catatonic behaviour, and negative symptoms including but not limited to flat affect and avolition. These symptoms had to have been associated with occupational or functional dysfunction, present for at least one month if untreated or a shorter duration if treated. Additionally, the symptoms could not be due to a direct physiological effect of substances or a general medical condition. Those who were eligible were approached for enrolment. Recruitment occurred until data saturation was reached and no new information was recorded. A total of 23 patients were approached, of which four were excluded because they did not report a history of trauma (*n* = 3), or declined to speak about their traumatic life experiences (*n* = 1), resulting in a total sample of 19 participants.

### Study procedure

Individual in-depth interviews were conducted in English, isiZulu, or a combination of both languages, based on the participant’s preference to allow the participants to describe their experiences fully using culturally-specific expressions. All interviews were conducted by the principal investigator (VN), a clinician with experience in the field of psychiatry and trained on qualitative research techniques. The transcripts are available in the supporting information section (S1 Data). VN was also trained on collecting data on trauma experiences by an established trauma researcher (LN) to ensure safety of participants during data collection, particularly around ways to manage participant discomfort and distress. Participants who were interviewed had no prior contact or provider-patient relationship with any of the investigators and this minimized conflict of interest and interviewer bias. Interviews were conducted face-to-face in the hospital setting and psychological first-aid was provided to participants when needed. Participants noted to be distressed were referred back to the treating physician for further assessment and management.

Data was collected between 23 January and 20 March 2020. All interviews were audio- recorded and transcribed by a bilingual research assistant into English. The principal investigator randomly selected transcripts and verified the data for accuracy. Transcripts were anonymised and participant names were removed and replaced with pseudonyms prior to data analysis. For protection of privacy and confidentiality, participants’ real names were not used. Common Zulu names were selected instead for pseudonyms.

### Instruments

A semi-structured interview questionnaire was developed to guide the interviews. The interview guide included questions such as, “what difficult experiences have you experienced in your life?”, “what was most traumatic for you?”, “how do you think the events have impacted you in your life?” Demographic data and information on clinical diagnosis were also obtained from the participants and confirmed on clinical records.

### Data analysis

Data was analysed using interpretive phenomenological analysis (IPA) [21]. The three theoretical frameworks underpinning IPA are phenomenology (the subject’s lived experiences), hermeneutics (the subject’s interpretation of their experiences and the researcher’s interpretation of the subject’s interpretation) and symbolic interactionism (how meanings are constructed within a social and personal world) [21, 22]. In IPA, participants are considered to be experts of the phenomenon under study (e.g., psychosis, trauma) and the researcher examines the “insider’s perspective” of the phenomenon. The researcher examines how participants make meaning of their lived experience, while also simultaneously interprets the personal world of the participant by paying attention to what the participants may be less aware of, or are not saying in their narrative [23].

In our study, we coded for the descriptions of the traumatic life experiences and their impact (phenomenon) directly from participants’ narratives. We also paid attention to any unintended information provided by participants about adverse or stressful experiences that the participants had not interpreted as traumatic [23]. Any description of events that the participants described as traumatic, distressing, or having a lasting negative impact on their life (e.g., having memories of the traumatic event that cause disruption in their daily functioning) were coded as “traumatic life events.” Negative life experiences that were part of the participants’ narrative but were not made explicit by the participants as traumatic were also coded under this code. The same approach was used to code data describing how the traumatic life events impacted the participants’ lives. We focused on the symbolic interactionism process to understand how participants made sense of their traumatic life experiences within their contexts as individuals living with psychosis.

Nvivo v.12 was used for data analysis which was led by the lead author, VN, and MBD. Raw data was first coded using inductive coding [24]. A codebook was developed after jointly coding five transcripts and until consensus was reached and no new codes were created. All transcripts were coded using the final codebook including the initial five used for codebook development. VN and MBD discussed divergent codes and themes until an agreement was reached to improve the reliability of the results. Another member of the research team (NGW) also randomly selected transcripts to check the coding for consistency using the final code book.

### Trustworthiness

Constructs of credibility, dependability, transferability and confirmability were used to ensure scientific rigor [25]. The lead author (VN) reviewed results with other members of the study team and her clinical and research supervisors (BC and LN). This was further facilitated through analyst triangulation by the first and second authors (VN and MBD). Transparency and auditability were facilitated by qualitative data analysis software program. Detailed in-text quotations in the presentation of results are used to enhance transferability.

### Reflexivity

The principal investigator and lead author (VN) identifies as an African female researcher living in a South African context. Given that most participants in this study identified as African and shared a similar cultural context to VN, she was intentional about including other races to achieve a heterogeneous sample that was consistent with the racial distribution of the population of KwaZulu-Natal. The other members of the research team came from diverse cultural and contextual backgrounds, and this added to the objectivity of the findings in the study. During interviews, VN found herself immersed in the patient experiences as some of them, for example, corporal punishment, had been a part of the culture and norm growing up. Although she went into the research in order to understand, she realised that she had not completely divorced herself from the clinician part of her identity, and the misconception that clinicians have a better understanding about clinical phenomena and diseases than patients. It had come as a surprise to her to realise that patients were able to make the connection between psychotic experiences and past trauma experiences, exposing her bias about assumed knowledge of participants.

## Results

Descriptive statistics of the participant demographic information are summarised in Table 1. Most participants identified as male, had not completed high school (grade 12), were single, unemployed, and had a diagnosis of schizophrenia. None of the participants were on treatment for trauma-related symptoms. No participant appeared to be visibly distressed at the end of the interview. However, information revealed during the interview for one participant had to be disclosed to the clinician in order to optimise the treatment plan. Permission from this participant was obtained before disclosure. Most participants reported their first traumatic event occurred in their childhood or adolescence, and the index trauma event occurred before a formal diagnosis of a psychotic disorder was made.

**Table 1:** Sociodemographic and clinical characteristics of participants (*n* = 19).

	Mean (SD)	n	%
Age	28.74 (7.59)		
Number of traumatic events(lifetime)	3.31 (2.54)		
Gender			
Female		6	31.6
Male		13	68.4
Race			
Black		15	79.0
Other		4	21.0
Highest level of education			
Below grade 12		9	47.4
Completed grade 12		6	31.6
Tertiary education		4	21.1
Employment status			
Unemployed		14	73.7
Student		2	10.5
Employed		3	15.8
Marital status			
Single		16	84.2
Partnered		3	15.8
First/native language			
isiZulu		14	73.7
English		2	10.5
Other		3	15.8
Diagnosis			
Schizophreniform disorder		3	15.8
Schizophrenia		11	57.9
Psychotic disorder due to another medical condition		2	10.5
Brief psychotic disorder		1	5.3
Psychosis not otherwise specified		2	10.5
Cannabis use disorder			
No		14	73.7
Yes		5	26.3
HIV infection			
No		15	79.0
Yes		4	21.1
Age of first trauma event			
12 years and younger		10	52.6
13–18 years		9	47.4

### Experiences of traumatic life events

Three major themes emerged that described participants’ traumatic life experiences: 1) experiences related to parent-child relationship disruption during childhood and adolescence, 2) bullying and violence from peers and community members, and 3) experiences of psychosis.

### Parent-child relationship disruption

Disruption in the parent-child relationship between participants and their biological parents or parental figures occurred for almost all participants in several ways. For some participants, their parents or parental figures were physically absent due to death, while others were separated from their parents due to other adversities, like poverty or financial hardship. Participants with unemployed parents, for example, were sent away to live in other households with better financial resources. Some participants reported that they lacked protective, nurturing caregivers and safe environments in their childhood and adolescence, sharing narratives about traumatic events including residential instability, abuse and neglect. These findings further generated the following three sub-themes: residential instability, financial hardship, and child abuse and neglect.

*Residential instability*. Most participants reported not having stable housing during their childhood and adolescence. Reasons for being removed from the home included death of a caregiver or parent, or for engaging in/being wrongfully accused of engaging in minor transgressions. Participants described that they often moved from one unfavourable living arrangement to the next, and faced new challenges and stressors each time they transitioned to a new environment.

*I lived there; I grew up there* [at participant’s aunt’s home]*. My mother preferred I went there than other places… I lived there for about four years… Then I came back to live with my mother. Then I was chased out by my uncle* [who lived with my mother]*, I was probably 13. He kicked me out and told me to go away, he said I was rude. Then I left… I lived in…* [name of township] *with some boy I was dating. He rented a room for me.*

Sane

Bheki spoke about losing a stable home, both physically and figuratively, after losing his parents at a young age.

*My mum passed away when I was eight and my dad when I was 12. My dad passing away, let me say, I never thought much about this ever since he passed away. But what I realized was that the only thing that changed was the fact that I lost my home yeah. So, I became, I could say, homeless, I became a person who never had a permanent home, you know.*

Neli, who also lost her parents at age seven, shared that she and her siblings were frequently moved from one household to another. She recalled that she did not have a stable caregiver who would protect them and provide a stable nurturing environment for them to live and thrive as children.

*We’d get separated, taken in by aunts and uncles. One would live somewhere and have the experience of how things were done in that home. When you’d hardly gotten used to it, then you’re removed and placed elsewhere to live. They were changing us around all the time like that. It was hurtful because there wasn’t anyone to speak for us anymore. No one to intervene for us in all this experience.*

*Financial hardship*. Another common traumatic experience discussed by the participants was financial hardship, which often began in childhood. Parent-child relationships were often disrupted due to the caregivers’ lack of financial resources to raise a family. For example, for households where the caregivers were unemployed, they often delegated child care to others (e.g., relatives, family friends, neighbours). The extent of financial hardship was sometimes very severe and affected participants’ access to basic needs, such as food and schooling. Sizwe, who was unable to go to school after his parents’ death, recalled the challenges of having to support himself.

*After my aunt kicked me out, I went to live by myself. Then I looked for a school but then I couldn’t get in because they required a school fee of R1000 (7USD). …When I think about it, the most traumatic event for me was that I couldn’t finish school. I see myself as a failure, as a loser because I did not finish school… I was working hard jobs that were not paying much. I was also living alone and sometimes I’d run out of food. And that would force me to sleep without eating for several days.*

*Child abuse and neglect*. Most participants also reported experiencing various forms of interpersonal trauma, including child abuse (e.g., physical, sexual, and emotional abuse and neglect) by their caregivers. Experiences of physical abuse occurred in the form of frequent corporal punishment for these participants.

[My caregivers] *didn’t have time to relax with me, or talk to me as a child. I would get a strong beating… They used a stick. I had bruises, I had bruises in my arm. I would go to school with bruises. And the way they were, they would influence my teachers to beat me again, you know… And sometimes I confessed that I want to go to my father’s side of the family, and I got a beating for that too.*

Londi

Shane, a 21-year old man who received corporal punishment from his father as a young boy, recalled, “*growing up*, *my father used to discipline me a lot*. *I would get a beating with a belt or with a whip or anything*, *even like metal pipes*. *I used to catch a lot of beatings growing up*.*”* Another participant shared that receiving physical abuse, which at times resulted in serious injury, was a regular occurrence for her and her siblings growing up.

*…when we got beaten up, we were beaten using a water pipe.* [One of my siblings] *had broken hands* [after a beating], *as it is, she has the experience of not being able to use them properly.* [Another sibling] *was beaten up by uncle* [name] *until his teeth came out.*

Neli

Sizwe also described the ways in which he received verbal and emotional abuse by his uncle, as follows—

*…sometimes, my uncle sent me to the tavern for beers in the middle of the night. That man didn’t like me. He always shouted at me for lies, and lied about me… I used to get shouted at. I lived under that pressure all the time, he’d place mistakes on me that weren’t mine.*

Participants who lived with relatives or in non-kin households with many children were often ostracised or neglected, particularly if their biological parents were not living with them. Preferential treatment was also often given to children whose biological parents provided financial resources to support the household. Sibusiso described the neglect and abuse he received from the caregivers he lived with. He was denied basic needs offered to the other children in the same household, such as a bath with warm water.

*I went to school, and* [my caregivers] *wouldn’t even buy me uniform and I was begging people… I was the only one getting beaten, they had many children but only I got a beating. If I’d gone to play only I would get beaten. Only I would be left to bathe with cold water.*

Participants also discussed experiences of child neglect by their parents/caregivers (e.g., neglect of their physical safety or emotional needs) and other poor caregiver-child relationships that lacked warmth or nurturance. Neli described the neglect she received from her parents when she was injured, as follows—

*I’ve had the experience that when I was a child, I got injured… by a window and it left a scar. Then I got burnt even on the body, burnt by water. I was living with my parents; I could say that they were negligent to me. Thereafter the parents passed away. When the parents passed away, we lived with granny. And when living with granny we had to live with aunt, and aunty didn’t treat us well, she treated us* badly.

Other participants were sexually abused, often by their caregivers or people they knew who were in a position of authority. Sexual abuse also co-occurred with other forms of abuse, such as neglect or child labour. Participants’ family members often turned a blind eye to the sexual abuse and in such cases, participants were left to tolerate the abuse alone. For some participants, their family members silenced them through fear and threats. Sane recounted her experience of being sexually abused by her uncle who was also her caregiver. The uncle was also the primary breadwinner of the family who provided her basic needs.

*[My aunt’s] man used to sleep with me forcefully… He slept with me and said I must keep quiet because I used to work and live there and did cleaning and everything. He was the one who bought me clothes and everything, he bought me clothes and my aunt would be furious and say I receive everything from her man… I never told my mother because she said [that] they [would] say I’m involved with him. I won’t tell my aunt, and I won’t tell my sisters too, because it’ll be as if I came to ruin. My aunt once swore at me and said I ‘make things happen’[seduce], at the beginning, when I tried to speak up. … And he was not doing it to me only. Everyone who came [to the house while] my aunt was working… the uncle would sleep with them.*

### Violence

Some participants reported that they experienced violence, including bullying, ostracism, and discrimination, from their peers or community members. Simangele was bullied for having a stutter and further punished for reporting the peer to adults for his actions.

*…The issue of being bullied and I have tongues, they call it stuttering. I call it tongues because if you say its stuttering, it hurts… They’re picking on me and I tell them I don’t like it… You get laughed at by everyone. And in a painful way, not laughing normally, they loud laugh, and they even come up to the face laughing. When you go home to report that person because they’re older, instead of [the perpetrator] being fetched from his home and told never to do it again, I’m the one who will get a beating.*

Similarly, Zara spoke about being bullied in school for doing well academically—

*I was always teased in school and sometimes they used to call me stupid. And they used to, like, make fun of me. And I used to do well at school used to get trophies and gifts and stuff, but they used to be a bit jealous. So yes, that’s their way of getting back to me, teasing me all the time.*

Sibusiso described the police brutality he experienced for not being able to afford clean clothes. He recalled, *“…there have been times when I… when I’d been beat up by police*, *beat up by security*. *Uh… a lot of securities*… *they beat me up and people [in the community] too…”* Another participant, Mpendulo, was forced to receive treatment. He reported that, when he was an adult, he was taken to a traditional healer against his will by his family for experiencing mental health symptoms. He was restrained to cure a mental illness that, at the time, he did not perceive himself to have.

[My family] *said we should get inside the car, and I got in the car. And it was obvious we were headed somewhere. We went—I didn’t know where we were going. My wife was not being told anything, nothing was said. And we parked at some traditional healer. I was taken and put on chains until my tummy would swell.*

### Experience of psychosis and receiving the diagnosis of schizophrenia

Some participants discussed that the experience of psychosis itself—the symptoms and being treated for them—was traumatizing to them. Perceptual disturbances, or hallucinations, and delusions were highlighted as traumatic experiences for these participants. A participant with an olfactory hallucination of his body odour described the following:

*It’s the odour. It’s just a smell from my body. I bathe, brush my teeth, but I just have a smell. I am just aware of it. It’s just the same smell that bothers me because I seem as if I’m not like other people. I think people will see me as someone who doesn’t love myself if I’m going to come up smelling…*

Thando

Bheki identified that his most traumatic experience was receiving the diagnosis of schizophrenia from his mental health provider. He recalled the reaction he had when he first heard about the diagnosis he was given:

*I never thought I [would] ever be diagnosed with such an illness. I never thought [that], actually, I [would] ever take treatment for my brain. Yeah, so, I always think, “what did I do?” and so, that’s a traumatic problem for me… “what happened?”, “where did I fall off?”, “what went wrong?”*

### Post-traumatic experiences

Participants also described mental health symptoms that were characteristic of post-traumatic stress symptoms based on the criteria set forth by the *Diagnostic and Statistical Manua*l [20]. These symptoms included flashbacks of trauma memories and avoiding thoughts about the traumatic experience or event [26]. Psychological distress and negative moods often followed flashbacks for Londi. She described how the heightened level of fear due to her trauma remained long into her adulthood, impacting her day-to-day life.

*It’s like something that plays on television but it’s in your mind. You have those flashbacks when you were a kid being hit by… hit by your grandmother. Getting beaten. You have those memories, and also that the memory that your mother died. All these things come back together and cause my mood to be… to be bad… At some point, when I’m in a… when I’m in panic attacks, it comes back. But sometimes it’s those traumatic experiences… when they come back, they overwhelm me… And these memories come back and haunt me… You know when I think about it… it’s causing me a memory of hate.*

Londi

Participants also shared that they engaged in maladaptive (e.g., substance use, withdrawal) and adaptive (e.g., physical activity) coping strategies to help them avoid thinking about their traumatic life events.

*I just don’t want to go anywhere because I’m afraid. You know, it feels like I can stay alone, do a lot of things alone, and social withdrawal and stuff… I would just become numb and I feel like going away and hidin’… I feel like going away when I experience a lot of shouting… Like, I’m always working out gym-ing. I’m always busy, so when I’m busy, I don’t get time to think about this, and when I come back from the gym I’m always tired I don’t have time to think about this. But when I stop gym-ing and stop doing these activities, you know, I stay alone a lot. And these memories come back and haunt me*.

Londi

Shane’s experience of childhood abuse led him to turn to substance use as a means of escaping distressing emotions.

*All I know [is that] I cried, and in my mind, I just said, “I hate this man” and, like, “why am I suffering?” and stuff. In the long run, when it came to high school, I realise that there are things I could do to escape. Like, for example, alcohol and drugs and stuff, so that’s when I started indulging in those things.*

Shane

Cognitive impairment was another mental health symptom reported by participants. Sizwe, who was repeatedly exposed to domestic violence through witnessing her sister getting assaulted by her husband, reported how intrusive memories of the trauma impacted his learning at school. He recalled, *“at school I couldn’t concentrate because I heard her voice because I’d wake up in the night and she’s calling me painfully*. *Because from her voice you could tell she needed help*.*”* Another participant, Nolwazi, was abandoned by a boyfriend when she fell pregnant and later discovered he had infected her with HIV. She could only give vague and general descriptions of the event that occurred 17 years earlier, unable to provide details as she had forgotten what happened.

### Making sense of the traumatic experiences

Two themes emerged that described how participants made sense of their past traumatic experiences. Participants described loss, post-traumatic stress symptoms, and the inter-relatedness of their experience of psychosis and trauma.

### Loss

The two main experiences of loss reported by participants were, 1) the loss of a sense of self or self-identity, and 2) the loss of a position in society or social identity. These losses were felt as a consequence of both the trauma experiences and having a diagnosis of a psychotic disorder. For participants who reported that their first psychotic episode or receiving the diagnosis was traumatic, recounted how their condition significantly impacted their sense of self. Some described it as losing touch with their inner self, or their identity, and they no longer knew who they were and did not feel in control of their behaviour. Participants described a loss associated with who they thought they could be in the future.

*Yeah, so,* [being diagnosed with schizophrenia] *has been the worst part for me… and for the world to know that I will never be myself again. I mean, like, I’ll never be able to not take treatment because I’m going to live on this treatment for… I’ll be taking the treatment for as long as I live. That’s the traumatic part… the fact that I’m diagnosed with schizophrenia. I’m not like somebody who’s not.*

Bheki

For Zara, her mental illness negatively impacted her self-perception and her belief about the world around her. It was challenging for her to have lost a sense of control in her life due to her psychosis and the trauma she experienced from it.

*The difficult thing I found to deal with is waking up every morning and not knowing how I’m going to behave towards the next person. It has totally changed the way I think and feel about the people around me and about society and about nature and about myself. It’s totally taking over my mind that it becomes a whole negative impact.*

Zara

Participants also described the loss of their identity within a community due to past personal traumatic experiences. This was the case for Sane, a 35 year-old female who had been sexually abused by her uncle. She described that the experience of losing her virginity due to sexual abuse meant losing her position within her social context, which resulted in being excluded from ceremonies like “The Reed Dance” [27], a community-wide rite of passage event that publicly celebrates and honours virgins.

*…there was that “why did it happen to me?” You see, wishing that I was also normal like other kids… and I didn’t have that experience. Because another thing that was painful was when people entered the reed dance… and as a child, I couldn’t go to the reed dance because I didn’t know where my virginity had gone.*

Sane

Sexual trauma also led to feelings of undue self-blame and loss of power and freedom to choose one’s identity. One participant described that their experience of being molested at a young age led to unresolved internal conflict of their sexual orientation.

*I was very angry at myself. Because I said, “how could this happen to me?”… Now, years after the event, I still feel traumatized. I still feel my dignity got lost. It caused a tension, like* [having a sexual identity, or sexual thoughts, is] *a wrong, wrong thing. It opened a wrong door, thinking that it’s right for me… to, to fantasize about myself with another man.*

Luke

### Interconnectedness of psychosis and trauma

Some participants shared insights into how their childhood trauma experiences manifested as psychotic symptoms, either around the time of abuse or later in life. Bheki had a history of severe childhood physical abuse. As a child, he would engage in repetitive play, mimicking the abuse he received—a common clinical expression of re-experiencing trauma among young children [20]. He conceptualized that this childhood play behaviour was related to his trauma and also considered it a symptom of psychosis. Bheki also shared his discomfort around the diagnosis of schizophrenia, which has led him to minimally report his symptoms and behaviours to his provider.

*I do remember when I was young, uhm, my mum used to hit us. My dad used to abuse us. So, I think maybe that’s where the whole thing started because that’s why I used to hit sticks you know. Like, I used to play with sticks alone. Like, I would take sticks and play with them, like, hit sticks. I would hit sticks as if, like… as if I was a crazy person. But I’ve never spoken to anybody about this. I think about it sometimes but then I don’t. Nobody knows. Even the doctors don’t know that. Because the way I look at schizophrenia, there are some things [that] can be considered as reasons why I was diagnosed, you see. But I don’t talk about it because every time I talk about it, it’ll come to be as if this person was supposed to be diagnosed with schizophrenia. But I don’t feel comfortable with being diagnosed with schizophrenia.*

Londi also recalled that growing up in an environment where she remained constantly guarded against physical abuse made it more likely for her to experience paranoid delusions.

*In the beginning of my sickness, I thought everybody was out to harm me, you know? So, getting beaten psychologically made me feel like most people want to harm me… when my sickness began, I felt, like, “oh, everybody is out to harm me. Somebody is going to cause me to suffer.” Only to find out that the people are not going to harm me. It’s all in my head.*

For others, their experiences of past trauma and related memories were more pronounced in their psychotic symptoms. In other words, the content of the psychotic symptoms were directly related to the traumatic event they experienced, Zara, for example, spoke about her constant paranoid delusions of reference—thoughts she described to have started when she was the target of bullying in school which she iterated, *“it was the teasing*, *and sometimes they used to call me stupid*. *And they used to*, *like*, *make fun of me*.*”* Her past trauma memories of being excluded and feeling judged by her peers was described as a form of intrusive thoughts that were also not reality-based. “*The day-to-day becomes weird*, *it becomes something weird to me*. *Certain times*, *I feel as though everyone around me has something to say all the time*. *They’re going to judge [me]*.*”* Simangele, a 30-year-old male was also a victim of bullying at school as a child. He was frequently targeted and ridiculed by his peers. He recalled, *“they’re picking on me and I tell them I don’t like it… [but I] get laughed at by everyone… and in a painful way*, *not laughing normally*, *the loud laugh and they even come up to [my] face laughing*.*”* This memory left a lasting impact on him and as he later began experiencing psychotic symptoms, he reported hearing sounds reminiscent of the laughter of those childhood peers who tormented him. *“It began when I was still young*. *It still happens even now*, *when I am about to fall asleep*, *I would hear noisy things as if it’s a lot of people screaming and laughing ‘Ye-e-e-a-a-h-h’ and drum like things ‘dudududu’*.*”*

Some participants reported the overlapping experience of psychosis and trauma symptoms. As Neli, who was sexually abused multiple times in her adolescence, perceived her stress symptoms to be part of her psychotic symptoms. She described, what appeared to be a panic attack, it felt like shadows around her were *“holding”* her breath.

*I used to see shadows coming towards me, as if they’re going to press against me, wanting to kill me… yes, I felt like I was being pressed, as if I’m going to die. It was hard to breathe, it was hard to breathe*.

Sane, who was also sexually abused by a relative, fell pregnant from an older boyfriend and had a miscarriage at the age of 14 years.

*There’s just a voice that says she won’t complete school. There is a voice that just keeps saying inside my ears, that this one is going to get pregnant. And I keep praying for her over and over. And her father and grandmother become angry when I pray for her and told not to pray for her because the thing keeps telling me to pray for her. And hold her here, in the stomach because she’s had a problem; there’s my sister’s child that came for a visit and stayed there at my house… He tried to sleep with her. I caught him, and he tried again.*

Sane’s auditory hallucinations, hypervigilance, and stereotypic psychotic behaviour she exhibited, resulting to her hospital admission, were related to her and her daughter’s past trauma experiences of sexual assault.

## Discussion

The present study examined the lived experiences of people with psychotic disorders, focusing on their descriptions of traumatic life experiences and how these experiences have influenced their lives. Three main themes emerged from participants’ accounts of past traumatic experiences. The first theme, parent-child relationships disruption, captures adverse experiences stemming from disruptions or changes in family structure during individuals’ early childhood years. The findings elucidated that separation from biological parents had a ripple effect, extending to other adverse experiences that were traumatic. These experiences encompassed poor living conditions, financial hardship, and abuse and neglect. Unstable family structures, characterized by constant changes in family members or being raised by non-biological parents, are common experiences for children in the general population in South Africa [28, 29]. Experiences of parental or caregiver disruption can have lasting effects through one’s life-course. Early parental disruption can consequently lead to increased vulnerability to stress and a greater likelihood of mental health problems for these children as they develop [30, 31]. Furthermore, disruption in one’s living situation during childhood through frequent relocation and separation from social networks can significantly increase distress in children [32]. Non-nurturing or abusive caregiver/parenting experiences in childhood can also result in poor caregiver-child attachment and higher risk for physical and mental health problems later in life, including psychosis [33–35]. High mortality rates and labour migration have been cited as reasons behind the fluid family structure in the country [28], which was also evident in our findings.

The second theme describing traumatic life events—violence—was reflective of the pervasive contextual stressor of inequality experienced by South Africans, particularly those residing in marginalized urban communities. Data from low- and middle-income countries show that urbanisation has exacerbated social inequalities and poverty, disproportionately affecting the urban poor compared to their rural counterparts [36, 37]. In their South African study set in the largest most populous province with high urbanisation rates, Breetzke [38] reported that suburbs with higher familial instability had higher levels of interpersonal violence and contact crimes. Communities in resource-poor residential areas have limited to no access to meet basic needs, and such social inequity increases rates of violence and crime, which in turn threatens human security [39].

The third theme centred around participants’ report of psychosis as a traumatic experience. It has been documented that the trauma of psychosis can be severe enough to lead people to experience distressing symptoms that meet DSM criteria for PTSD [40]. One systematic review reported that rates of PTSD from experiencing symptoms of psychosis and receiving treatment ranged from 14–47% [41]. In their cohort of patients with FEP, Dunkley, Bates and Findlay [42] found that the trauma of having psychosis (e.g., perceived enforced treatment, paranoia around caregivers and fellow patients, having the illness as an ongoing problem), extended beyond the first episode.

The presence of PTSD symptoms among patients with psychotic disorders can complicate the clinical presentation and pose challenges for providers for making appropriate diagnoses and treatment plans. According to DSM-5, PTSD diagnostic criteria is met if an individual has direct or indirect exposure to a serious traumatic event, including actual or threatened death, serious injury, or sexual violence [20]. The individual subsequently experiences intrusive symptoms such as recurrent dreams or memories relating to the event, avoidance of stimuli related to the event, and, two or more negative alterations in mood or cognition. Alterations may include inability to remember important information about the event, negative beliefs about oneself or the world, or inability to experience positive emotions. In clinical practice, it is often difficult to distinguish between psychosis and PTSD symptoms, particularly if the conditions are comorbid, as some of the symptoms may be similar [43]. It may be difficult to distinguish flashbacks of past traumatic events, a symptom of PTSD, from visual hallucinations, a symptom of a psychotic disorder. Similarly, the negative cognitions of PTSD, such as a negative view of the world, may present as paranoid delusions, while negative symptoms of psychosis may be difficult to differentiate from loss of interest experienced in PTSD.

While participants in this study were not assessed for PTSD, we found that most reported symptoms characteristic of post-traumatic stress symptoms including re-experiencing, avoidance, hyperarousal, and poor cognitive functioning. Similar findings have been reported in another study conducted in sub-Saharan Africa [44]. In a qualitative study investigating trauma in patients with serious mental illness and other stakeholders in rural Ethiopia, participants reported experiencing post-traumatic symptoms commonly seen in the local context as well as those consistent with Western-defined set of clinical criteria [20, 44]. Rates of PTSD are also reported to be higher among those with schizophrenia compared to the general population in South Africa [10, 12]. In a South African multicentre case control cohort, 94% of patients with psychosis reported having experienced at least one traumatic event in their lifetime compared to 90.5% of those who did not have psychosis [9].

The third research question explored how the participants interpreted and made sense of their traumatic life events. Participants discussed loss of self- and social identity, experiencing mental health symptoms which were characteristic of post-traumatic stress, and psychotic symptoms that resembled or were connected to their memories of their past traumatic experiences. Changes in self-concept, including losing a sense of self or identity, are common experiences among people who experience psychosis and/or PTSD [42, 45–47]. Waterman [47] describes identity loss as a possible impact of trauma and this identity loss may either have brief or have a lasting effect on mental health outcomes.

Participants in our study shared that the content of psychotic symptoms was closely related to their trauma symptoms and/or memories. This finding is consistent with existing work proposing the interconnectedness between past trauma and the nature of psychotic experiences, or that past traumatic experiences could be re-experienced during a psychotic episode [48, 49]. In a study of patients at clinical high risk for psychosis or diagnosed with a psychotic disorder where about half the patients reported child abuse and 38% also met criteria for PTSD, most believed that past trauma experiences impacted their psychotic symptoms [50]. Similarly, in a qualitative study of patients with trauma and psychosis, participants reported that their trauma experiences were related to their psychosis, while some shared that their psychosis may have been prevented had their trauma experiences been addressed by their providers [51].

## Limitations

The study has some limitations. First, some phrases expressed in the isiZulu language by the participants did not have direct translations in English. Therefore, some nuances in meaning may have been lost which may have compromised the quality of the data. We attempted to minimize any translation-related problems by using a study team that consisted of an interviewer, translator, and co-coder who were all fluent in both languages and had similar cultural backgrounds to that of the participants. Second, this study used retrospective data on participants’ recounts of their past trauma experiences which may have contributed to recall bias. However retrospective self-report on childhood trauma has been shown to be reliable in populations with psychosis [52, 53]. Third, culture-specific trauma symptoms did not emerge as a theme in our study, and this may possibly be because of the nature of open-ended questions in our study without a direct question about culture-specific trauma. Another local study found 20% higher rate of PTSD when using a culture specific tool to screen for PTSD [54]. Finally, given that this is a qualitative study conducted with a specific cohort of participants, transferability of findings is limited and cannot be applied to other settings or populations.

## Clinical implications and future direction of research

The findings of this study underscore the complexity of the association between trauma and psychosis among people with psychotic disorders with a past traumatic life experience. Similar to existing research, the distinction between psychosis and trauma symptoms remained arbitrary [55]. It is likely that environmental factors together with cognitive, behavioural, and affective symptoms influence the development of post-traumatic stress and psychotic disorders [56]. The underlying mechanisms, pathways, and the directionality of the relationship between trauma and psychosis are still unclear and more research is needed.

Our study also highlights the need to adopt trauma-informed services in early psychosis care in South Africa. Studies have consistently found higher trauma rates and a higher risk for meeting criteria for PTSD among people with psychosis compared to the general population [9, 57]. Despite reports of post-traumatic stress symptoms, none of the participants in our study were being treated for trauma-related conditions at the time this study was conducted. This lapse in clinical practice is a global trend. Studies conducted in well-resourced settings also report the low integration of trauma interventions in clinical care, despite high trauma rates seen among patients with psychosis [58]. The importance of screening and treatment of trauma-related symptoms or PTSD in clinical practice has been recommended by other researchers in the field [49, 59]. Clinical guidelines to promote trauma-informed care for psychosis have been developed in some countries [60, 61]. The use of trauma-informed practices can lead to appropriate and timely diagnosis and treatment that jointly address trauma symptoms and psychosis [62]. More implementation research with similar populations is needed to change current standards of clinical practice in South Africa.

The impact of past traumatic experiences can have long-term effects and should not be left un-addressed in clinical practice. We recommend clinicians managing patients with psychosis to exercise caution in focusing solely on the psychosis diagnosis, and instead to thoroughly address all symptoms and past life experiences that may be causing distress to the patient, particularly after the acute and overwhelming psychotic symptoms have subsided. Trauma-informed care does not need to be limited to clinical settings. Our study revealed that childhood trauma due to parental disruption was a common experience among our participants, which highlighted the need for trauma-informed parenting support or interventions to prevent family trauma. Community or school-based interventions for youths in South Africa should incorporate positive parenting/caregiving strategies to minimize risk for child abuse/neglect. Additionally, more social welfare programs that provide financial support and resources are needed to support families and caregivers who care for multiple children to prevent family separation and poor living conditions for youths. In Southern Africa, positive caregiving after the death of one or both parents has been shown to foster resilience [63]. More research in this area to promote positive mental health outcomes among youth and prevention for child trauma in South Africa would contribute to improving child psychiatry practices. This, in turn, could help prevent development of serious mental illnesses later in life.

## Conclusion and contribution

The present study addresses a gap in literature and provides further clinical insight on how trauma presents among people with psychotic disorders in South Africa. Results from this study underscore the importance of addressing personal trauma histories in the context of psychosis treatment and supporting patients to understand the complex relationship between post-traumatic stress and psychosis. Further research to understand the underlying mechanisms or processes linking trauma exposure to the onset of the first episode of psychosis could inform early identification and intervention for young people who may be at higher risk of developing psychotic disorders. Additional research is also needed on culturally sensitive psycho-social intervention strategies for low-resource settings to address the existing treatment gap in mental healthcare in South Africa.

## Supporting information

S1 Data Transcripts of participant interviews. (PDF)
