# Supplementary material for: Experiences of trauma among persons living with psychosis in KwaZulu Natal, South Africa
Source: PLOS Ment Health. 2024 Oct 3;1(5):e0000070. doi: 10.1371/journal.pmen.0000070 (PMC12798335; doi:10.1371/journal.pmen.0000070)
Supplement: S1 Data — (PDF) [file pmen.0000070.s001.pdf]

Transcript for Interview QTS01

VN: Please tell me about traumatic experiences that you've had. You say that you've had a few

Participant : yeah

VN: aha

Participant : Ok it will be like growing up, my father used to discipline me a lot. I would get a beating with a belt or with a whip or anything, even like metal pipes. So that was like number one for me Like on traumatic experience. I used to catch a lot of hiding growing up. And uhm yeah that's like…and then other than that I think uhm relationship-wise you know like with a girlfriend or something. I've been involved in a lot of relationships and it didn't work out well. Like I've been heartbroken and stuff A lot and that's why I choose to be single right now.

VN: Ok

Participant : even though I've got like friends and stuff but like I still keep my distance. Yeah I don't open up very easily and yeah that's just like me

VN: Ok so when you say you don't open up easily you mean...

Participant : Like it's hard for me to... well to make friends I'm good at that, like speaking, I'm outspoken and stuff. But like when it comes to the more intricate stuff, like personal feelings and stuff like that, then I choose just to avoid it.

VN: Oh ok so you mentioned Relationship trauma and Trauma with your dad giving you a hiding, which one do you think was the most traumatic experience?

Participant : It's hard to say

VN: You're not sure between the two?

Participant : yeah I'm not sure

VN: oh ok I'm going to ask you to tell me about both experiences then. So how old were you when you when your dad started, what was the first time More or less the age?

Participant : I can remember like...Say about... well from a young age I know he used to discipline me as a small child Like under 5 years

VN: aha

Participant : He used to discipline me. So yeah and it's been going on like ever since

VN: Okay until?

Participant : the last time I think was last year. Last year

VN: ok. When you were five Just give me an example of how it would happen. How it would start.

Participant : I can remember one incident where I made my brother cry. My baby brother. I was small and maybe my baby brother was 1 at that time. I made him cry for something and then he took off with me and he hit me in then yeah

VN: Oh how old was your baby brother?

Participant : About one or two years

VN: and how old were you at that time?

Participant : About 5 6

VN: oh okay. And what happened exactly did he take something

Participant : He hit me with the belt

VN: and how long did the Beating go on?

Participant : Until it was...for me it is like it never ended. And he just like carried on. He kept going overtime I developed like a hate for him. Like even now he's my father yeah, I respect him yes but I don't love him.

VN: hm

Participant : It's very hard for me to love him. Like even when we're talking in the group and stuff, I can't be myself around him I always have that like fear that I got for him And yeah that's me.

VN: Ok and with the incident that you remember about the beating, how did you react at that time?

Participant : All I know I cried and in my mind I just said I hate this man. And like why am I suffering and stuff

VN: aha, during the time. And in the long run? How have you been feeling?

Participant : In the long run I rebelled when it came to heist I was a top student I should get like several awards and Stuff in primary school. But when it came to high I realise that there are things I could do to escape. Like, for example, alcohol and drugs and stuff, so that's when I started indulging in those things.

VN: Ok so there are thing...you're saying there are things that you started doing?

Participant : hm

VN: Okay and how did that affect your life?

Participant : Well I wasn't getting a straight A's anymore that was one of it

VN: aha

Participant : So I was joining the wrong company and getting into trouble. But like I didn't care I was just like I got too much worries at home and I'm just going to enjoy my life And not like...because like now that I look back on it I understand, like I stopped everything. I only smoke cigarettes now and like my parents know that

VN: aha

Participant : but like Other than that when I was in school and stuff with friends I would just be like ey I'm out of this house and he just gets on my nerves. Like he shouts for everything in house doctor, like everything. Just...ok I'm working now right, on my days off what do you do on your days off? You want to relax

VN: aha

Participant : no you can't relax because you wake up early or wash the dishes, Go wash the vehicles, clean the yard, Sort the car out. I mean it's my day off, I work hard and I like some time to rest like and you even can't watch your own programs. If you're watching a Cartoon for example I like dragon balls, it's...it's I know it's like childish but like I grew up watching that and I enjoy it. But I'm not allowed, I'm going to be [age] this year but I can't watch certain things on TV and he doesn't allow me and it just gets frustrating.

VN: Certain things like what?

Participant : like watching a program or something

VN: aha

Participant : Like a TV program he says no it's bad and this and that. And I mean I know other friends they go clubbing and all, my age, I don't do that but yet he still...It's hard to explain like everything he does Like I can't wait to get a nice earning job And get out of the house and live on my own and I'll see from there

VN: ok. Do you somewhat, feel responsible for what happened to you?

Participant : There is someone, the responsibility I take because I chose to do that led me to my life that led me to schizophrenia

VN: aha

Participant : But I also blame like other people. Well not blame them, if I look back I know it's my fault. But I see the actions and the, the path that led me to what I did. And I know God is there and nothing happens for no reason that he has a better plan for me I know that. I'm a Christian I believe in God very strongly and yeah I just feel that things happen for a reason.

VN: So you're saying that there's a path that you could have, should have taken? You could have taken a different path? Or this is a path that was meant for you?

Participant : Well you see there could be a different path right. In primary when I was small I used to picture myself going studying Living a good life, going out with friends and stuff. But like right now I only have two friends, and they come and see me like I'm not allowed to go out with them and stuff. And like everything is very restricted. But it was always like this that's why I started to rebel. Like even from small I started having a girlfriend. My dad wouldn't send me for her party and parties and stuff. And then, then I just rebelled. Yeah I just rebelled

VN: Ok so I asked earlier, do you think there are things you should have done differently?

Participant : Well it could have been

VN: hm

Participant : I'm sure there is

VN: Or other people should have done differently

Participant : yeah

VN: ok what are those things?

Participant : I mean like in my father be more caring and loving. Like I'm 100% sure I wouldn't have started indulging in all these things, these wrong things. And he also used to like fight with my mother and stuff, like hit her and stuff. So like I grew up not knowing how to love the opposite sex. I always viewed it as that was it. So whenever I got in a relationship I wouldn't like love completely

VN: hm

Participant : like a couple months ago, I think the longest relationship I have been in was for about a year and then I would always cut it off. I've had plenty of relationships but I always end them when things start getting serious.

VN: So what do you think caused that?

Participant : I think it was the relationship between my parents

VN: Ok so you mentioned things that could have been done or happen differently in your life that would have affected, that would have resulted in you in a different path and maybe not ended up where you are. Do you think there's anything that can be done now? Differently

Participant : As I said I stopped indulging...well dagga was my number one weak point

VN: aha

Participant : Because I started with that. Well I have stopped that. And whenever I used to smoke I could hear the voices

VN: whenever?

Participant : Whenever I smoked dagga

VN: ok

Participant : I could hear...I still hear voices now and then. But it's like good, like decision making. Like if I need to go somewhere I'll always ask myself and then they'll be like yes or no and then like motivation wise. Like just the other day I woke up early, I was off, the weather was nice. It was about 9:00 10:00. I said it's a nice day to go run, then I heard a voice say like [name]. I hear things like that

VN: hm

Participant : it's like motivation and decision-making. So in a way I don't look at this illness as something bad. I, I believe I am like special to even have this, because people always look for that voice

VN: aha

Participant : Maybe it could be god if you believe in God. Maybe it could be something else but I look at it in a good way and I think I'm special because I have this illness.

VN: ok so when you think back about the traumatic experiences does it ever give you palpitations, do you ever have flashbacks about it? Do you ever have any...?

Participant : yeah I do

VN: okay what exactly do you experience when you think back on your trauma?

Participant : I can, I can picture it in my mind and then I'll think about why everything happened. And if it's like a bad thing I'll just, I'll just go outside and smoke a cigarette. Like that's my escape.

VN: aha

Participant : I just escape that way

VN: ok is that why, how you react to every other bad thing that happens in your life? Or is it specifically when you think about your childhood?

Participant : It's hard to say. I never thought about it like that.

VN: Okay no it's fine. And if there was an Intervention that someone developed and they said maybe it's a relaxation technique would, to whatever you are feeling, having those bad feelings and you're thinking about what happened, would you be interested in taking up something like that?

Participant : uhm Yeah I would but obviously like a work shift and sometimes I start at 4 in the morning and like till 10 in the night. So it's like a bit difficult for me to commit to anything

VN: If it's something that you can do at home, some relaxation technique wherever you are would you do it?

Participant : yeah I would

VN: would you be willing to do it?

Participant : yeah

VN: Oh okay. And is there any, any other trauma you would like to talk to me about?

Participant : hmmm

VN: Or experience? You said something about relationships

Participant : yeah

VN: okay, what happens just describe to me how, what happens in a relationship when you start a relationship, and what really traumatizes you or gives you pain

Participant : hmm I just think back to my first relationship. Like I loved that girl. And because she broke my heart and like she left me for an older guy in the same school. I actually wanted to be like that older guy and that older guy was indulging in dagga, drinking, listening to all types of music and his lifestyle was very opposite of what I was at that time. But I said like if this girl Leave me for him and I really liked her, I was like ok I'll become like him. So Then I started joining him doing all those things and stuff and then after that every relationship I got into I had like no feelings. Normally you get those butterflies and stuff but I never get those. Then I think my last relationship in school that was the longest relationship I've had. And that was, that was something special. But because it was getting serious I had to end it. Because I can't handle like confrontation and that seriousness. I've always been that joker type of person

VN:

Participant : So when it came to that serious type, part I just let it go. But when I finished school like about one year later I regretted it. That's when I tried to commit suicide

VN: Ok

Participant : I overdosed from pills that I got from the pharmacist, with alcohol

VN: What pills were those?

Participant : It was something for pain, something

VN: aha

Participant : And I overdosed on that. That night that I overdosed I was supposed to go to work. I left I didn't go to work, I went to the bar. I got some pills I went to the bar and I added them in...I was drinking beer and hot stuff, everything I was mixing it. My intention wasn't to mix...to go for suicide. I was just like...I was out of it at that time, I was not thinking straight. And then that happened and I couldn't sleep that whole night. I just stayed awake playing with things in my room and stuff. And then the next day was my mum's [number] birthday

VN: aha

Participant : So then they were drinking and stuff and then I started drinking. And then everything was still in my system. So that's when I lashed out and I started fighting with my dad and stuff and we fight a lot. Like ever since I got big we fight a lot

VN: aha

Participant : Like just earlier today I actually borrowed my mum's phone because she has Wi-Fi on it. She's got a Rain SIM card or something else. So then I borrowed it so I can come to (*unclear*) I said I'm going to be here for 3-4 hours, let me keep the network and play with it. And then as I'm coming here my dad phones me and he starts (*unclear*) with me "why you took the phone I'm paying for the phone and paying for the network" and then everything is about money.

VN: hm

Participant : Like I'm paying for it you've got no say. And when I was not working it would drive me crazy because I would hear words upon words. And the medication that I'm on made me used to sleep in, I used to rest a lot. Like getting used to...because when you in the wine, you only do is rest. Your body, like I was very thin after I got out of the wine gained a lot like 20 kgs

VN: hm

Participant : Yeah so now when your body gets used to that you become like, slow, but you didn't understand that. Ok he gave me leeway for like the first couple of months. But after that he was like "go get a job you can't sit at home". And I understood where he was coming from his point of view but like give me some more time. I mean yeah

VN: Ok so you said something about suicide, was that before you got diagnosed and started medication?

Participant : no that, that was what led me to getting into hospital.

VN: Ok so you were upset about a girl that you ended up ended a relationship the Year previously?

Participant : hm

VN: It just came, you were thinking about it?

Participant : hm

VN: Okay were you having any flashbacks, were you dreaming about it?

Participant : No but I dream a lot, like I dream a lot like every night like three four dreams. And sometimes my dreams come true. Like for example, for getting a job I dreamt that I'm going to be late on a certain day I'm going to meet a friend who was in [city]. And that actually happened

VN: hmmm

Participant : Yeah another time I dreamt that the van was going to get into an accident, our van and I'm going to get a job. It was a weird dream we went to a park that day we got a puncture and we met someone who's actually hiring. They hired me and I got the job.

VN: hmmm

Participant : And now I'm dreaming that I'm going to get accepted into the [occupation type]. And I've applied there so now I'm just waiting for that. If that comes true it'll be like my dream

VN: Oh that will be a dream come true

Participant : a real dream come true, that'll be my dream job. Because after school my aim was to get experience in every field so I know where I can stay And further my career forever like till I retire. And after school I'm sure you'll be shocked at it, guess how many jobs I've had

VN: It hasn't been long since you left school so

Participant : [year] I left school

VN: uhm...I don't know

Participant : take a guess, a random guess

VN: 3, 4?

Participant : 15

VN: (*laughs*)

Participant : 15 jobs

VN: wow! So this will be your ultimate job?

Participant : Yeah a [occupation] because I've worked at I've done [occupation], in a [workplace], I've worked in [workplace], in [workplace], everything. This job that I have now is just so I can earn some money, so I can support myself. Because My Parents They used to buy me like cigarettes. That's like the only thing I need right now

VN: ok

Participant : I only, I only need that other than my phone data and stuff. But like things are hard at home as well. So I got a job I also help...I give half my salary to school…uh to my home. I just got this job now and I wrote that test in...Uh [month] I think. [month] or [month]? One of those dates I wrote...no [month] actually. I wrote it in [month] so I'm just waiting now, they started calling my friends and stuff and then letting them know and they haven't been successful. So they haven't called me yet I'm just praying I'll be successful

VN: yeah you're hopeful?

Participant : Yeah I'm hopeful

VN: oh okay and then tell me about, I want to know around the Time when you almost committed...was the girl still around in your life?

Participant : no

VN: Oh so you were not in contact with her?

Participant : hm

VN: Okay and how did that make you feel when you realise what had happened?

Participant : I've, I wasn't thinking...at that time even through Hospital, I didn't think about the whole thing. I just thought in my mind it was like a game to me

VN: It was a game?

Participant : yeah. Like that schizophrenia part played a trick on my mind as well. Like this whole thing is just like a game for me. That I'm playing it and play it to the best of your ability. Whatever is (*unclear*) to you then just keep going forward.

VN: hm Ok. Okay it's been very nice talking to you. Thank you so much for sharing your experiences, I'll stop the recording now.

Transcript for QTS02

VN: Okay so just like I've already explained to you, one of the things we are interested in in our study is to help people cope with difficult situations in their lives. I would like you to tell me about some of the difficulties that you've experienced in your life.

Participant: uhm some experiences?

VN: yes difficult experiences, traumatic experiences that you have experienced

Participant: I was molested at the age of 5

VN: aha

Participant: And then it continued and made me think that I was over then everybody else yeah

VN: Oh okay with that a once-off incidence or is it something that continued?

Participant: It continued again when I was 7 years old

VN: So it was once off when you were 5?

Participant: yes

VN: And then again when you were 7?

Participant: yes

VN: Oh ok tell me more about the incident. What happened?

Participant: uhm Another guy he told me that I must, that He wants to take my penis and put it in his I must put it in his bum, so that's what happened

VN: Oh okay

Participant: and then that's what caused the whole thing

VN: you say it caused the whole thing?

Participant: It caused a tension, like [having a sexual identity, or sexual thoughts, is] a wrong, wrong thing. It opened a wrong door, thinking that it's right for me… to, to fantasize about myself with another man.

VN: aha

Participant: Instead of being, just being just being a child and Experiencing a normal life of a child

VN: hmm ok so was this incidence the most difficult one you've ever experienced, the worst thing that ever happened to you?

Participant: Yes

VN: Okay so why exactly was it, do you think about above everything else, it was the most difficult?

Participant: Because uhm I had to, I just think and say okay this thing happened to me now how will I go forward now, go forward in life from now on to whenever I meet my life partner. And how will it affect the our marriage or our relationship being together

VN: Okay is that what you thought when you were 5?

Participant: no. It's only now recently

VN: Oh ok so it happened when you were 5, how did you react at the moment? Like after it happened

Participant: I was very traumatized uhm I was sad. I didn't know, I was confused because I was still young. But my mother told me everything that was going to be okay. And it'll all work well on me and that I have a bright future

VN: So immediately after it happened what did you do?

Participant: uh we went to the police station to go report the matter, me and my mother. We went to go report the matter at the police station

VN: aha. Ok so he made you do what he made you do. And then afterwards immediately what happened?

Participant: And then uhm oh! Then afterwards with him, what happened with him?

VN: aha and with you what were you thinking, how did you react? How did you...

Participant: I reacted in a very sad way. Like a very bad way I was very angry at myself. Because I said how could this happen to me

VN: aha

Participant: And I remember I don't know what to think, or what to do at that age. I was still young, the tender age of 5

VN: so how did your mum come to find out about it?

Participant: I told her

VN: When, how long after the incident?

Participant: straight after. I need to go to the toilet please doctor

VN: Ok it's fine, ok take a break

VN: so now it's been quite a few years after the event

Participant: yes

VN: how do you feel about it, when you think about it?

Participant: Now, years after the event, I still feel traumatized, I still feel my dignity got lost

VN: aha

Participant: In the event because that's your, your that's what you keep with yourself, you don't just give it away to anybody

VN: aha

Participant: It's very precious to you

VN: hmm. So when you say you feel traumatized can you describe that kind of feeling for me?

Participant: I feel sad and I feel down and out, sometimes when I think of it. But I just pray and I say Lord please help me to forgive those people that have done the wrong to me

VN: Okay so you've just mentioned that you pray

Participant: yes I do pray

VN: okay is there anything else you do to cope? What do you do to cope with the event?

Participant: I pray and I do some sports sometimes I do sports

VN: aha

Participant: And anything to occupy my mind yeah

VN: Ok so sometimes when something bad happens to people they change the way they think, their behaviour. Uh have you ever noticed that in other people?

Participant: Yes it's because uh As soon as something bad happens it triggers the mind. The mindset of a person is changed forever

VN: aha

Participant: Because uh that's also part of their life now. It's the scar inside them knowing that they got molested or sexually assaulted by somebody else

VN: Is that what happened to you?

Participant: yes

VN: Ok so you're saying your mind has become changed forever?

Participant: but not forever because in a Christian we believe in God you can renew your mind With the Bible when you can carry on and read the scripture

VN: Has that event affected the way you do things on a daily basis now?

Participant: No I still do the things the same way (*unclear*)

VN: So it hasn't changed the way you do things?

Participant: no

VN: but you say it has changed the way you see yourself?

Participant: Yes

VN: oh ok. And at work you said you're not working at the moment?

Participant: yes. But I used to work before

VN: where?

Participant: I was a [occupation]

VN: Oh okay

Participant: at (unclear)

VN: Oh really? So did it change the way you worked

Participant: no it didn't

VN: It didn't affect the way you... so what do you think helps you cope?

Participant: I think what helps me to cope?

VN: aha

Participant: is me just, me just not running around talking about things. And not stressing too much, I mustn't stress a lot. So I can be able to cope and manage myself well

VN: aha. Is there anything that you think should have been done to prevent the situation from happening?

Participant: yes I think I should have been told earlier on by my mother or somebody else and they were not allowing us to touch you inappropriately

VN: Ok and after it happened is there anything that you think could have been done to help?

Participant: uh

VN: Afterwards, do you think there's anything that can be done now, to help with the way you're feeling?

Participant: yes I think there can be. I think there can be awareness, there could be talking about just this whole situation. And how we go through and how we go through as people who are mentally disturbed. Because of certain problems in our lives

VN: aha Okay so you're saying that some people get mentally disturbed because of certain problems in their lives?

Participant: yes

VN: do you make a connection with your condition?

Participant: yes

VN: Okay what is the connection, what do you make of it?

Participant: I think uh that, that the recognition that is if this thing didn't happen to me I would be more closer to my dreams or be more far further in my life, but that happened to me.

VN: aha

Participant: But I always say god is in control because God knows why this happened to me and so I can be a living testimony to others and share the God's word to others

VN: Ok that something that you do?

Participant: yes I do

VN: Oh okay. Is there anything else that you would like to talk to me about?

Participant: No that's all

VN: ok so sometimes people with schizophrenia like you've mentioned, or mental illness uh get to be forced to come to hospital things get done to them. Have you experienced any traumatic event in that way?

Participant: No no

VN: Oh ok, so your admissions have been without any stress or trauma to you?

Participant: yes

VN: Or discomfort?

Participant: no discomfort everything is okay

VN: Oh ok no that's fine. So those were the two incidences? It was only two times

Participant: yes

VN: Okay and the events that happened when you were seven?

Participant: at the age of 7?

VN: aha

Participant: There was another guy

VN: someone else?

Participant: yes. That told me I must do the same thing

VN: aha

Participant: And then I thought it was normal because I was still young at that time. So I ended up doing it again

VN: Aha and how was that now that it was happening again?

Participant: it was very sad again because at 5, at the age of five I still remembered what Happened at the age of 5 to me. But now at the age of seven it was very hard for me to, to put myself to to bring myself together. But I eventually done it

VN: it was hard for you to?

Participant: Bring myself together because of what happened. Because when I, I started beating myself again doing the same thing

VN: so you're saying you are you were...you did it to yourself?

Participant: No I'm saying I repeated the same thing again

VN: Oh ok

Participant: So it was difficult again

VN: so did you take responsibility for the 2nd Event that happened to you again? Did you feel responsible for it?

Participant: Yes I did feel responsible for it. I sort of prevent it from happening

VN: How old was this person who...?

Participant: he was my age

VN: he was your age?

Participant: yes

VN: Oh okay and you blamed yourself?

Participant: yes I did

VN: Oh okay did you ever have thoughts about it?

Participant: yes

VN: Later on and flashbacks, or nightmares, what exactly did you experience?

Participant: no uhm just thought about it and how traumatizing it was for me

VN: OK considering that Incidence what do you think should have happened to prevent it? Do you think anything should have happened to prevent it?

Participant: uhm I don't know what I'm going to say. But I think the only thing that's going to help me to prevent it, was me being uh me being more vigilant

VN: aha

Participant: And knowing that some people are out, they are out there to hurt you and steal from you, your joy and whatever your uh say whatever you say is true in your life but they make it false because they wanted to steal joy and they are Dream stealers

VN: Ok when you say they are Dream Stealers you do recognise that the problem is with the person who is doing it?

Participant: yes

VN: Not with you as a young child

Participant: Yes

VN: ok because that's what you need to understand, is that you are not responsible for what happened and you're not responsible for your safety as a 7 year old

Participant: Oh okay

VN: You need to understand that. Okay we'll talk further this is the end of our interview for now. But we'll just have another chat about what you've just said.

VN: Ok like we've already spoken and you have agreed one of the things we are interested in is how to help people cope with difficult situations

Participant: Ok

VN: I would like you to tell me about any difficult situation that you have experienced in your life. What are the difficult situations you have experienced?

Participant: Well uh the one difficult situation that I've gone to or that I have experienced is being diagnosed with schizophrenia

VN: being?

Participant: being diagnosed with schizophrenia

VN: oh! Being diagnosed with schizophrenia?

Participant: yes

VN: oh ok uhm…

Participant: I realized that uhm Because looking back, looking back Towards the years that I before being diagnosed, you know, I realized that uhm...although I wouldn't say I wasn't supposed to be diagnosed with schizophrenia but I'll say, looking back I can say Ever since being diagnosed, my chances of becoming a Somebody That I Once wished to Be Have decreased. Not because I'm not someone who is capable of making something out of my life but Because of the ill...of the fact that I've gone to realize my, my weakness as a person, you know, who is diagnosed with schizophrenia. In other words I'm saying but That's has helped me now because it helps me realize if Just because I'm diagnosed with schizophrenia doesn’t mean I can't do anything you know. It just means that I need to be I need to work harder into being somebody. Yeah because before now I was able to uh...I never used to like laugh unnecessarily you know. And I never used to... I used to think but my thoughts were not too much you know. And I’m also used to...I was a lonely person

VN: you were a...?

Participant: I was a lonely person

VN: lonely?

Participant: Yeah

VN: ok

Participant: Because on my chill time I used to stay in the room all the time alone writing lyrics, you know the songs? Yeah But then ever since I got diagnosed I started having these thoughts like you know what I never thought I never thought I would ever be diagnosed with such an illness. I never thought that, actually, I would ever take treatment for my brain.. Yeah so I always think “what did I do?” and so that's a traumatic problems for me. Like what did it do because before this time I was not like this you know and Like what happened, where did I fall off, what went wrong. Yeah so that has been the worst part for me to and for the world to know that I will never be myself again. I mean like I'll never be able to not take treatment because I'm going to live on this treatment for...I'll be taking the treatment for as long as I live so that's the Traumatic part. But the best I've learnt that I can do is just not think of that too much

VN: So I'm going to take you back a little bit you said it shows your weaknesses?

Participant: yes

VN: In what way?

Participant: Yeah, so, being diagnosed has been the worst part for me… and for the world to know that I will never be myself again. I mean, like, I'll never be able to not take treatment because I'm going to live on this treatment for... I'll be taking the treatment for as long as I live. That's the traumatic part… the fact that I'm diagnosed with schizophrenia. I'm not like somebody who's not but People Somebody who, like if I could take like two people like a person who is identified as excellent person in terms of health And then the person who is like me you find that that uh You find the person that is considered excellent in terms of health and me you find quite a lot of similarities Will be taking salaries compared to me because you know I'm Schizophrenia you see. Or the fact that I laugh unnecessarily sometimes. Because I realized that sometimes I just Smile and be like and then later people would be like be like “Why was this smiling?” But I won't say it out loud because I don't want people to think I'm talking to myself you know

VN: aha

Participant: sometimes I would think a lot, Sometimes I would act aggressively when I'm angry you know those kinds of things. So those are traumatic stresses you think “Why am I like this there people who don't do this to me what happened to me? Why?” You know

VN: And then uh saying that it's a traumatic Exp...It’s a traumatic thing for you to be diagnosed with schizophrenia, have you had any incidents related to hospital and admission for your condition that have been traumatic?

Participant: well honestly there's none that I remember actually I’ve Never. I do remember when I was young, uhm, my mum used to hit us. My dad used to abuse us. So, I think maybe that’s where the whole thing started because that’s why I used to hit sticks you know. Like, I used to play with sticks alone.
VN: what sticks?

Participant: Like I would take sticks and play with them like hit sticks. I would talk to sticks like "hey what up" and go on. Sometimes I think maybe...

VN: so you're saying when you were young you used to play with sticks?

Participant: It was not a normal thing from what I've seen

VN: aha

Participant: Because I've never seen anybody do this thing But I noticed that most of them would play with sticks. You know those made man those plastic those playthings

VN: yes

Participant: I would hit sticks as if I am…

Nurse: (comes in) hi doctor how are you? You are ok now?

VN: Oh okay

Participant: Like, I would take sticks and play with them, like, hit sticks. I would hit sticks as if, like... as if I was a crazy person.

VN: you would hit sticks?

Participant: take a stick and hit it like this

VN: oh. Ok When you were young?

Participant: no no I was...I stopped that about [year]. [year] is when I stopped that. I think I was growing up. Yeah it wasn't normal for me sometimes (*unclear*). I think about it sometimes but then I don't, I don't nobody knows, the people that I want to inform, all know. Even the doctors don't know that

VN: hm

Participant: Because I don't want, because the way I look at schizophrenia there are some things can be considered as reasons why I was diagnosed, you see. But I don't talk about it because every time I talk about it, it'll come to be as if this person was supposed to be diagnosed with schizophrenia. But I don't feel comfortable with being diagnosed with schizophrenia

VN: Ok so are you saying that you tried to avoid things that make you get identified as a schizophrenic? As having schizophrenia

Participant: aha

VN: You tried to avoid such uhm Instances?

Participant: Not that...I've been trying to avoid them but I realized that avoiding them would not help me with anything. So it's best for me to talk about them (*unclear*) it’s even more traumatic. Because when I tell somebody something about my real about my past it tends to be a nightmare

VN: you were saying it feels like a nightmare?

Participant: yeah because I think about what a person is going to think of me after I tell them, you see. Because people as well when you tell them something they won't understand it because you tell them because you are seeking help. They feel that you're telling them something as if you want like you want them to feel shame for you, which is obviously the point. But also if you look at it they take something as if Ok ok ok when you are gone and they are the only ones and they’re whispering I never thought it was this crazy how can he hit sticks blah blah blah

VN: But how old were you at that time?

Participant: I was still playing hit sticks when I was young I was about eight. The last time I remember I was 8, 8 9 10 11 12 13 14 15. I think I stopped, I think I stopped at the age of 13 or 12. Yeah I was still young

VN: Okay so when were you diagnosed with schizophrenia?

Participant: I was diagnosed at the age of 16

VN: So at that time how did you react to the diagnosis?

Participant: You see the only thing is that I found out I was diagnosed with schizophrenia when I was I think I was 18 or 19. Because I was taking the treatment although I never knew what was the treatment for, you know. I only got to know a few years later, also not from the same hospital that I took it from. I got to know when I was admitted in [hospital] and if it wasn't for me looking at the form I would have never known what was the treatment for, you know. So I had the discharge a little bit, they would tell me about the treatments where I'm supposed to fetch the treatment. They referred me to the place where I'll be fetching treatment I looked at it already and I was diagnosed with schizophrenia and epilepsy “huh I didn't know I had schizophrenia”. From there I was still trying to find out what is schizophrenia. People were telling me it was a brain illness I was like “what, so I'm a crazy person”

VN: hm

Participant: And I was so shocked because where I was staying There were kids who are taking treatment here in this hospital before But they were taking the tableton you know tableton?

VN: aha

Participant: not a not a…what's this...not a Risperidone. And most of them don't take care of me, so I'm still concerned that we'll never get out of this treatment, like how? Because there were kids who were taking their tableton but they're out. Even people who were taking tablets for insomnia but it got healed. Like how come... it was a traumatic experience for me

VN: Oh ok so afterwards… uhm so you're saying you discovered this very big thing, and then what happened?

Participant: after discovering

VN: what did you do about it?

Participant: after discovering it, it's just that I wasn't informative. I wasn't informative literate in terms of what to do. But also that but also that I find it hard to express my feelings, or My thoughts in a way that (*unclear*) So for me I wasn't sure what to think. I didn't tell anybody until I came through the hospital and I got taught More about schizophrenia thing no schizophrenia is this kind of Where you've got a bright brain, oh actually I asked the doctor. I started asking the doctor questions like doc I realized, I just found out that I'm schizophrenic do you know why I'm schizophrenic? What the doctor did was he she open the file Checked a few notes and she told me know what there was a time where you Took cannabis, maybe cannabis is what could have made me schizophrenic

VN: aha

Participant: I started thinking oh yeah I can believe that because I did take cannabis. I don't even know if it's possible that cannabis could make you coo coo or something

VN: Okay and that explanation how did that make you feel?

Participant: Well to be honest, it's more of a few things. Because that cannabis I was smoking it wasn't of my will

VN: It wasn't of your?

Participant: my will

VN: oh Ok

Participant: I was forced by some friends, my bro...My cousin's friends. I was with them and they were smoking cannabis. They were like come on try this, try this and also by the fact that I wasn't from the area I only was visiting in that place. It’s just that they had this thing of saying that you're old and you're quiet. Because back then there was a person who was quiet who never used to socialize a lot, you know what I mean. Then they just brought me this whole thing and I still do it it's not like a small thing like I took a spliff you know (*puffs)* I started coughing

VN: aha

Participant: I don't know how that happened that I have schizophrenia because even if you don't smoke it the way you're supposed to smoke it, you don't get addicted to it. I don't know if you smoke it quite a few times that you do but others smoke it many times I've never been addicted to it because I never smoke it properly you know.

VN: Oh ok so how does the whole thing make you feel?

Participant: You mean this whole...this whole...

VN: diagnosis

Participant: It leaves me asking questions. Yeah it leaves me asking questions

VN: aha what kind of questions?

Participant: Like, like should I... questions like what is it that I did actually, to yeah I knew this cannabis part, what did it do why me? Why did I have to be the person to be diagnosed with schizophrenia? Because when I was in school, you know, in school I was an A student

VN: aha

Participant: And I dropped out of, I went smoking...in primary though. Got to High School, became a, not that I became a...not that I became Weak, not that I became stupid rather but I became a learner that...like I became I wouldn't say a useless person but No good person in terms of education. I was not good in terms of Picking up, my mind wasn't that good at picking up knowledge. So I dropped out of school. That's why I'm actually...I'm supposed to be working. If I was like...if I did finish my matric I would be working pretty well. I would be owning my own, I'd be renting a better place now with my own girlfriend and maybe having children as well but Because of those reasons because of being diagnosed with schizophrenia I couldn't manage to get there, because After being diagnosed I started I was on treatment.

And then the treatment was, the treatment was still, the treatment had not adapted to my body so. I was still trying to, I was still trying to get it used to me. I was still trying to get used to it so that's why at school I didn't do well and that's why I dropped out because I thought it was just a

waste of time. I am just coming to school, I am not picking up anything. I would sit there from 8 to 2 but there is nothing that I learnt so I left. And now today I look I've got nothing but I know I can still work out something because my Mind is now better. I'm just saying what I know only, I was like let me ask song questions about why this thing because I don't know why I am like this. I didn't know why did I have to go through a time a phase I mean

VN: And then you get these overwhelming feelings that you've just described but you don't know why? What do you think makes you cope because here you are looking healthy and handsome and doing well for yourself in a way so what makes you cope what what makes you carry on?

Participant: Well I think it's been god

VN: aha

Participant: also there's a friend of mine that just came today His name is Josh. I think its people like him and God. The fact that I, the fact that I can just put my mind to understanding that the fact that I can put my mind into understanding that uhm sometimes just because I've got schizophrenia doesn't mean I'm actually a useless person you know.

VN: Great

Participant: and I picked up that, as I told you that I don't mean to say that I'm useless. Remember I said something like that? But it just makes me think like what happened. Because I do get motivated sometimes, I do get demotivated sometimes so I'm still in a phase where one moment I’m motivated one moment I'm demotivated. I realized that for me to keep being motivated I need to stick around people that are motivated

VN: aha

Participant: But also the problem now is that, is that Most of the time I find it hard to keep those people because when I'm around them I also have to be motivative. So you can't just be with somebody and expect to get from them but never give back. I think for me when I come out of there It's important for me to read more books, Learn more things, watch videos that are Motivative, focus on one thing and I've already got a plan so yeah. I know God...

VN: What is the plan?

Participant: well the plan is Because we do get Grant There is This thing I remember that like out of every intelligent guy...I was asking my mum I tried to get a good deal In terms of some mixes then I'll get my mum to cook for me something, Try to get something there. Write my book in my spare time. Because I'm already writing a book a motivational book over my life It's title is “within me is purpose driver” So within me there’s a purpose that is driving. So I'm trying to write a book which...I have already written a lot of pages It's more like a one page thing because it's poetry, emotion. So I realized that like I need income, like I'll need income to push the book

because The Grant that I have is not much and also Substitute for my family Anyway I'll ask my mum. the plan that Is just a come out of here Try to find a good room, Room from a necessary factory Fresh and work out a way of how to sell them and then Make money and work on the book

VN: Ok perfect that sounds like a very brilliant plan. At least you have a plan and it's very nice.

Participant: yeah

VN: I'm sure you will succeed

Participant: I know

VN: So you're saying you know?

Participant: yeah I know

VN: That's great

Participant: and I believe that I will succeed

VN: ok

Participant: Yeah because they say words tend to be action. So If you believe something...there is a saying that says what the mind of mind can be conceived, and believe it's going to choose you

VN: Ok so you mentioned that being around motivated people motivates you. Anything else?

Participant: Well it's also... it's also talking about my problems That helps me understand that You know... because When I talk to people that's when I feel de-stressed you know

VN: aha

Participant: Like now I'm talking to you and you listening to me. Being listened to Alone makes me feel like you know this is phenomenal. Because Imagine having a problem in talking to yourself you can hear yourself but you can't tell yourself, answer yourself you can't give you a self Solutions. As much as you can hear yourself but you know what you're saying but you don't have a solution so yeah.

VN: Ok so sometimes when people get bad experiences when something bad happens to them Or something difficult Happens to them they go through A difficult phase uhm It changes the way they behave, it changes the way they think And the way they see life. Uhm Have you ever noticed that?

Participant: yes I've noticed it

VN: aha tell me about it

Participant: After being admitted in [hospital name]. Pulling the treatment I had a friend a very close friend I remember it was [date]. I trusted that one day I believed it was for me being admitted there I believe she would be supporting me. So she kind of walked away, was not there so for me I started seeing people I mean like friends as people that are not there for you. Like I started believing that there was nothing like friends

VN: so you changed your belief?

Participant: yes in terms of people

VN: aha you didn't believe that people were friends?

Participant: No I didn't believe they were friends, there was something called there were true friends. They were People who were just pretenders you know

VN: Oh okay is that happened to you?

Participant: Yes

VN: So now that's what you think about everyone?

Participant: no no that's what I thought of everyone not now. I don't think like that now

VN: And now?

Participant: well let me just say

VN: how has the event changed you?

Participant: what events changed me?

VN: Yeah how has been diagnosed changed you? How has it changed the way you see people?

Participant: Well as I say that at that time I had that problem

VN: aha

Participant: But also I believe that this treatment has, this treatment is doing a bit of help in terms of us. Because that was before that was just the beginning. I just started taking treatment so that's how I saw people back then when I just began taking treatment. But as I started taking it I believe that it's healed my mind. And also seeing my spiritual dad also he gave me more motivation to, he's made me think that be the way I was thinking it's because I'm not happy I'm just not okay Right now but at some girl I will be ok. So how I see people now is I see them different. It just depends on the person that you move with, you know.

VN: and in your day-to-day activities has it affected you in any way? Knowing that you are diagnosed as in what you do go about your daily routine wake up in the morning

Participant: hm

VN: Do you think about it every day? With the diagnosis yes. Has it affected the way that you continue with life?

Participant: No, not much. The only problem that I have is when I'm Reading my books I find it hard to catch the stuff that is written. It takes a while before I can Cram the thing that I (*unclear*)

VN: ok and how Do you...what do you think helps you cope with stressful... with the effect that it has had on you?

Participant: uhm...

VN: What makes you cope?

Participant: What helps me cope is Music but its Christian music it's house music stabilize my mind and once my mind is stabilized I can...

VN: So it's stabilizes your mind?

Participant: it stabilizes my mind. It has helped me like calm down once that has happened and I can focus on positive things

VN: So you would want something that is going to calm you down?

Participant: Yes

VN: is that what helps you... is that what you're saying helps you cope, things that calm you down?

Participant: yes

VN: Ok would you get into a program where they will...something that's going to teach you how to breathe calm down would you do it?

Participant: calm down?

VN: yes

Participant: I wouldn't mind as long as there's something that's going to help me that's going to keep me focused on being positive. I wouldn't mind

VN: No perfect. You mentioned something about your parents I didn't hear what exactly you were saying

Participant: You mean my mum or my dad?

VN: yes

Participant: I said my mum passed away when I was eight and my dad when I was 12

VN: okay and how did you what was that like for you?

Participant: When my mum passed away was 8 and I wasn't that mature so I didn't understand much what happened. When my dad passed away was 12 so I kind of saw and understood a bit of things. So when my dad passed away I cried with that although we were not close much

VN: oh did that have any impact in your life in any way?

Participant: maybe it did because right now I don't have a permanent home

VN: You don't have a?

Participant: permanent home

VN: Oh okay

Participant: because my dad passed away

VN: So where do you live?

Participant: well I'm supposed to be like I'm supposed to be seeing a social worker but I've decided that I'm going to cancel that and talk to my Mum because I don't want to go to an institution again. I grew up in those places and I don't want to be held in a box. Hey want to be able to experience myself as a person. The way that I've been given, I'm purposed to the point where I can do things In advance

VN: okay so you have a mum?

Participant: I have an aunt

VN: Oh

Participant: I call her my mum because she's my mum's sister

VN: Oh so she's been sort of your mother figure?

Participant: Yeah but we've had issues in the past I'm trying to...I mean I just want to give it another try to just push doing that

VN: oh Ok so the passing of your dad, you say it was difficult

Participant: yeah

VN: uh how did it change your life?

Participant: My mum passed away when I was eight and my dad when I was 12. My dad passing away, let me say, I never thought much about this ever since he passed away. But what I realized was that the only thing that changed was the fact that I lost my home yeah. So, I became, I could say, homeless, I became a person who never had a permanent home, you know.

VN: how was that like for you?

Participant: Well uh it was uhm It has been very difficult because as a person who was having, as a person who was having a home, you can imagine how a person was having a home you can imagine going around staying with people and they are (*unclear*) because I've got a grant there. Staying with people you know staying with people is like they only like you because you have money Mirror. And once the money is finished they treat you like dirt. But if I had my mum or my Dad or my dad I'll be staying with them even if there was no money I'd be able to sleep at home. So I'd say that it devastated me made me feel like a used item instead of an appreciated person

VN: Used?

Participant: they made me feel like a used item, instead of an appreciated person

VN: oh ok. And then has that impacted the way you see life in any way?

Participant: yeah yeah I would say it has helped me to realize that in life you cannot trust everybody. There are people that you need to trust not because you want to make them happy or Because you appreciate their way of treating, the way they treat you. And there are people you need to understand that when they are brought up Maybe they are just people who just don't care about you

VN: But you've coped through all of that

Participant: Yeah

VN: you've come out on top

Participant: Thanks

VN: because here you are. What do you think made you become that way?

Participant: As I said I believe its god

VN: Wow! No that's great. That's amazing is there anything else that you would like to share with me?

Participant: From, maybe from which side?

VN: No just your experiences. Do you have any questions for me?

Participant: Well I would like to motivate

VN: You would like to?

Participant: motivate

VN: Oh great

Participant: I'd like to say uhm Life is not actually an easy Road. Therefore to everybody who's actually going through a tough time I'd like to say just keep pressing, for there's a saying *there's always a light at the end of the tunnel*. So if things turn to be hard for now don't say it's the end but Say it's going to go. It's here to go for there's a song that says *it's going to be ok*

VN: All right

Transcript for QTS04

Dr. V: So, as I have told you one of the things we’re interested in is How people cope with the traumatic things that happened to them. Can you tell me about the traumatic experiences you've had in your life?

Patient: It's that I was raped. That's what happened to me

Dr. V: That's the traumatic thing that happened to you?

Patient: hm

Dr. V: You were raped, can you elaborate about your incident? What happened?

Patient: I was still in school learning English. I was told to take my books and go to the office

Dr. V: hm

Patient: When I got to the office, the teacher was following me I didn't realize he was following me and then he raped me inside the office

Dr. V: Inside the school?

Patient: yes

Dr. V: oh okay. So, was that a once-off incident or repeated?

Patient: Once

Dr. V: OK how old were you?

Patient: I was 18

Dr. V: Oh ok. In all of this can you tell me about that moment when it had just happened, what did you do?

Patient: He said to me that if I said anything he would kill me (unclear)

Dr. V: How did you feel about that?

Patient: So, I was afraid because he said he would kill me. I went home and I told them. And then at home, they told the police. Then he got arrested.

Dr. V: So, at the moment when the tragedy had just happened how did you feel? What did you feel?

Patient: What can I say, it was like, what can I say it was as if I was not in my senses

Dr. V: okay how sister?

Patient: I got disturbed mentally

Dr. V: how did you know that you were disturbed?

Patient: I knew by coming here to the hospital

Dr. V: So, before the rape incident has your mind ever been disturbed before?

Patient: No

Dr. V: Ok so immediately after do you remember what happened or you don't remember anymore? because of mental disturbance

Patient: Yes, I was disturbed mentally

Dr. V: So, what do you remember, where does your memory end about what happened?

Patient: (silent)

Dr. V: Since you became disturbed are there things you still remember about what happened or you're told by other people

Patient: no, I don't remember

Dr. V: you don't remember anything?

Patient: aha

Dr. V: do you remember when you were still in the hospital or you heard it from other people

Patient: No, the people were not telling me anything

Dr. V: Since you don't remember, do you still recall going back home to tell the story of what transpired?

Patient: yes

Dr. V: oh okay. So, you say this happened and then you went straight home to explain?

Patient: yes

Dr. V: But the mind wasn't okay? So how did you feel, what were you feeling at that time?

Patient: I wasn't feeling well

Dr. V: when you think about what happened to you at that time how do you feel?

Patient: I feel like crying

Dr. V: hm is there anything you feel on your body?

Patient: No

Dr. V: your heart does not beat faster?

Patient: no

Dr. V: Are there times when you think about it? Do you think about it all the time or just you don't think about it unless you're asked?

Patient: it is just that I no longer think about it

Dr. V: So, I mean you are here now, you have coped with it. What things do you think helped you cope and to continue with life after such a tragedy? When you think about it, what helps to do not lose hope?

Patient: it's the social workers

Dr. V: what did the social workers do?

Patient: they told me not to lose hope because I'm still going to continue with life, I can still continue with life

Dr. V: okay so other people, when something bad has happened to them They changed how they do things or they become a different person from who they were Before, for instance like what happened to you. Do you notice that in other people?

Patient: yes

Dr. V: Can you give me an example?

Patient: I don't know what I can say

Dr. V: personally, have never noticed any change? After the incident

Patient: Yes, the things changed in how they were happening

Dr. V: hm

Patient: Because even in school I used to pass but then ever since being raped I didn't pass well so...

Dr. V: So, it affected how you were proceeding in school?

Patient: aha

Dr. V: And then in life in general is there anything else you noticed about yourself?

Patient: No there isn't

Dr. V: the way you were, did your personality change from the person that you were?

Patient: No

Dr. V: okay you didn't become aggressive or reserved than before?

Patient: I was not quiet before

Dr. V: okay So you were a talkative person?

Patient: yes

Dr. V: Okay when you think about it, what do you think helps people in the situation such as yours cope? What do you think should be done?

Patient: hmm I don't know

Dr. V: If you were to meet a person alone in this situation what would you say to encourage them?

Patient: I would tell them to continue with their life

Dr. V: hm

Patient: And not focus on what happened. And continue with life because life goes on

Dr. V: Ok what happened to this teacher?

Patient: they got arrested and got released on bail

Dr. V: how did that affect you?

Patient: It really affected me because I thought he would come and do the same thing

Dr. V: did you say that or it was just in your mind?

Patient: I was just thinking

Dr. V: So, were you scared?

Patient: I was afraid

Dr. V: Oh, ok was he released forever?

Patient: Yes, but he lost his job

Dr. V: okay how did you feel about that whole thing?

Patient: (*silent*)

Dr. V: So, the fact that he got punished wasn't helpful? Did it make you feel any better?

Patient: Yes

Dr. V: oh ok. Uhm so now when you think about your life how do you think it would have been if the incident didn't occur?

Patient: I think that I wouldn't have (unclear)

Dr. V: So, your illness, did you begin being ill after the incident took place?

Patient: Yes

Dr. V: and before it happened, how were you?

Patient: I was fine without any problem

Dr. V: Okay is there anything you would like to tell me regarding the incident?

Patient: no

Dr. V: Ok sister. Thank you very much for your time and for speaking because, in the end, it's hard to speak about certain things on earth, especially traumatic ones. So how has speaking about it made you feel

Patient: (*unclear*)

Dr. V: It's as if something was relieved?

Patient: yes

Dr. V: So besides social workers have you spoken before?

Patient: Maybe with what kind of person?

Dr. V: uh your siblings, counseling psychologists

Patient: Counselor

Dr. V: so, you feel that speaking about it helped you?

Patient: Yes

Dr. V: oh, ok sister

Transcription for QTS05

VN: Yes

Participant: uhm I haven't had a traumatic experience where I've been robbed or you know raped or certain hectic experiences, I haven't had that. But I do remember coming on a normal day from school, and before that I was, the whole entire week, I was getting this anger outbursts. Where I was violent

VN: aha

Participant: and I didn't know what I was doing it was almost as if I was throwing a tantrum or misbehaving. That's what people thought, that was...that's what my parents thought. But when this happened I flung out a drawer with one hand and then I began to speak to myself and tell myself Things that were not happening in reality. It wasn't connected with reality. I was in grade 9 at the time it was term 3 When I got sick, it was term 2 term 3 mid-year. I came home and I flung open the drawer I was angry, I think I had a bad day but It was just a normal thing to come home and have a bad day. It's okay. but things got out of hand and then I had to be admitted in hospital soon.

VN: aha

Participant: Sooner or later I was admitted, I was told to go to the clinic, the unit 10 clinic [municipality] clinic. And I went there and they had to give me travel water to drink and they referred me to [location]. And I stayed in [location] for, from [month] in grade 10 because I passed the exam in grade 9. I'm just recalling all the incidents that happened at that time. I wrote the exam in grade 9 and I passed But I didn't get the course that I wanted So I was heartbroken as well. But I went on to go to [location] And at the time I was in grade 10.

VN: aha

Participant: I missed part of the year being admitted in hospital, Months on end. I was admitted in P3 as well after I got a transfer from [hospital]. I was admitted in P3 and I made friends, I made good friends who had a problem just like me: Bipolar, schizo. They claimed to be, this one girl her name was [name] she said her father hit her. That was her story behind why she was there. There was another girl named [name] she was also my friend. She gave me books she gave me things to do, things to paint. And I enjoyed it but I always cried and missed my parents.

VN: hm

Participant: It took them over five years to find the correct dosage on medication. But by then I found that I couldn't concentrate properly in mainstream School

VN: aha

Participant: So, I went to ABET which is an afternoon class. So, I went there and I learnt in level 4 in [year]. I went to ABET and I wrote the exams and got two distinctions. So that was a wow factor for everybody because I was still not well. I was not well at all

VN: hm

Participant: my mother came the entire year and sat with me in the class. Now it's 20...in [year] I went to level five. I made a deal with the teachers that I study matric for three years because of the age gap. You've got to be 21 and over to write matric in ABET Because it's an adult foundation phase. Just level five. So, I started in [year], I started [year] I sat in the matric year, Matric class for 2 years with different classes and different brains and new people. And I still learnt the same work over the two years this is [year] my last year to study. And to be honest I'm still not well because every day becomes a new challenge for me, it's a different challenge. Everyone has challenges every day, but mine is a different challenge because I have no source to my problem.

VN: aha

Participant: I haven't found a source to my problem but I've been going through therapy. The psychologist has taught me how to weigh the pros and cons. But when I get in this mood I speak about things I say I don't know what to do. I'm tired, the psychologist is telling me something different. I don't want to do what they say because I feel it's not helping.

VN: Ok so what exactly have you found difficult to deal with?

Participant: The difficult thing I found to deal with his waking up every morning and not knowing How I'm going to behave towards the next person if They ask me to do something or if they ask me a question. Because my mood just automatically changes the way I think. it has totally changed the way I think and feel about the people around me and about Society and about Nature and about myself. It's totally taking over my mind that When I think things it becomes a whole negative impact. I even made a file; can I take it out?

VN: yes

Participant: I made a file on mental health illnesses, I just stock up some teens, teens across the country feeling more anxious than ever. This is [name], this is [name]. And about a book this is (unclear) this is the awards function I danced. This is me I danced at the Awards function

VN: oh, it's pretty

Participant: uhm...

VN: so, you're saying it changed everything?

Participant: yeah

VN: what is it? What is the it you are referring to?

Participant: My...the way I think

VN: so, what changed it?

Participant: It's my thoughts that changed, the way I think. It's my thoughts, when I refer to it I mean my thoughts

VN: So are you saying getting, becoming ill...

Participant: Yes

VN: is what has caused your life to be unpredictable?

Participant: Yes, because I started to get these thoughts. It rushes through my mind, it comes with a gush. You know like when you're playing tug of war Every day?

VN: aha

Participant: there is one side pulling to the positive and there's the other side pulling to the negative. So, you're playing tug of war with negative and positive. I mean I'm trying, I'm trying my best to sort it out, but sometimes I just give up and Say it doesn't want to work. It's not... it doesn't want to work

VN: What doesn't want to work, your mind?

Participant: my mind. My mind and my feelings, the fact that I try to be in the correct posture, the correct mind frame, set mind frame and to...my parents take me to spiritual prayers such as the temple, the Brahma, to the guru where I can sit and meditate and have some calmness, some peace within myself. But I find that I've lost my faith in what I do, such as prayer.

VN: aha

Participant: Because I carry on wondering if there's a god that's going to help me Soon. because maybe, maybe they think I have a chance on me

VN: who?

Participant: my parents and the Brahmas and gurus, the priest. They say I have a trance on me

VN: a what?

Participant: a trance

VN: ok. What is a trance?

Participant: a trance is a... It’s something that, you go into a spiritual...like a holy spirit. It's something like that

VN: Oh okay

Participant: but this is in Hinduism. I wouldn't know to...

VN: Oh, so they say you have a trance in you?

Participant: yes

VN: Oh ok

Participant: but I don't seem to want to take it out. I always say I'm feeling moody, annoyed and irritable and upset all the time

VN: aha

Participant: I have such intelligent ways of handling situations certain times but when I go into these thoughts, the thoughts come. It's coming in like a Vision

VN: aha

Participant: a vision such as this scene. There's a scene it's almost as if I'm acting out like a play. You know when you play you see better? It's just like that in my mind

VN: Oh ok. So here you are, how do you cope?

Participant: how do I cope?

VN: yes, because you're looking beautiful, you are carrying on

Participant: I am carrying on but I cope by talking to my parents all the time. I like basically lean on them. But the thing is I want to not lean so much on everybody else my parents and just my aunt's, and uncle's. I want to do it on my own. I want to, I want to have to fight them because negative and positive are in a balance in my life. I need to balance that whole, I don't have everything like a balance routine that's going to help me cope every day. It's quite difficult but I

am coping everyday trying by trying and praying and just remembering what the doctor has taught me

VN: hm

Participant: But I always come to a solution where I say I just don't know what to do. I don't know what to do, I always say that. But it's just that I want to try, I am trying. But it becomes a weird, it becomes something weird to me certain times. I feel as though everyone around me has something to say all the time. They’re going to judge, they're going to...

VN: for example, have you had people judging you?

Participant: Not that I can remember of

VN: But it's something that you feel?

Participant: it's just something that I feel

VN: Okay so how old were you when you had these thoughts?

Participant: I was just 14

VN: you were 14 at the time?

Participant: yeah

VN: ok and how did it change your life?

Participant: It just, I began to try to commit suicide by taking medication that was un not aware of. I began to slit my wrist with a blade. I bought the surgical blade. I began to do these silly things because I remember when I was growing up I always said that committing suicide was not an option for me. Because when you're feeling down you've got to talk to somebody or you've got to deal with things in a mature manner

VN: hmm

Participant: I was always this mature girl. Little girl, as I was growing I was always mature and I never had those things in my mind to commit suicide. I was always the happy child

VN: So, what made you want to commit suicide?

Participant: The fact that sometimes I was always teased in school and sometimes they used to call me stupid, teased rainbow nation

VN: Why?

Participant: because I bent down once and then my underwear showed, it was a stripe underwear. So, this guy started to call me rainbow nation

VN: How old were you at the time of this teasing?

Participant: 13, 14 Around there

VN: so, what happened first was it the teasing or the thoughts?

Participant: it was the teasing, and sometimes they used to call me stupid. And they used to like make fun of me

VN: why?

Participant: because they were jealous. Because I used to dance. I used to do (*unclear*) and classical dance. I used to dance for the school functions at the fair at the child...uh charity fair and [place] fair. And I used to do well at school used to get trophies and gifts and stuff, but they used to be a bit jealous. So yes, that's their way of getting back to me, teasing me all the time.

VN: aha

Participant: It did, it did a little bit. But I eventually got over it, I'm over it now. but something seems not right today. I get angry, I get upset quickly. I just get this different kind of thoughts that I feel that are different towards everybody, towards everyone else's.

VN: What, they are different from what other people's thoughts are?

Participant: Yes, I just fee...I don't know what other people's thoughts are. But It just feels that mine's a bit different. Because right now with medication I have a story to tell you

VN: aha

Participant: It's basically a speech. Okay so good morning doctor

VN: hi

Participant: today I would like to enlighten you about what I understand about mental health. Mental health is first of all dealing with the psychological part of the brain, and its imbalances. For example, just like you and I have good and bad moods It is just something you should not be afraid to open up about. How we feel or be ashamed of mental health conditions. We as humans must live by a moto. My Motto of life is: do not sweep things under the rug, speak about your feelings, do not think critical thoughts. love yourself and find love from others in your daily

surroundings. This Is My Story I would like to share with you. I would like to tell you all about a story tale About a friend of mine whom I knew for many years, since grade R.

This young lady was a very brilliant and intelligent person academically and socially. About who she made friends with suddenly drifted away from her family and her friends. Now what I mean she drifted away I mean she dropped out of school on a regular basis. And was not very friendly but aggressive to all our loved ones around her. Her concerned parents took her for blessing, Around Every temple in the corner Not realizing she was mentally unstable. At last they took her to a psychologist. Who has passed spoke to her, diagnosed her, this dear friend of mine with a mental condition namely schizophrenia. It took the doctor's very long to diagnose her and put her on the correct treatment. She was Months on end in [name] Hospital. This good friend of mine is now stable on her medication and living a normal life in Society. The moral of the story is to not sweep things under the rug. Speak about your feelings, enjoy the new year 2020.

VN: A beautiful story. Thank you so much, how heartwarming. So, tell me about this story is this a story that you have written or a real story that happened?

Participant: this is a real story about me

VN: oh, this is your story? And it has a beautiful ending

Participant: yes

VN: So, you do believe that you have a beautiful ending?

Participant: yes

VN: Ok so tell me how did this, did you feel like that your life changed after the diagnosis?

Participant: Yes, it did

VN: in what way?

Participant: It not be meaning, it just, on certain days I have this challenge where I get angry and aggressive. Where I just want to break everything around me I just want to be alone or I just want to cry. So, in that case it just change my mood. I have these mood swings that just take over me.

VN: Ok

Participant: and it also changed my life in a sense that I cannot be around people. like for Example, in a working environment you have to be hands-on, we've got to be wise, we’ve got to be in control of yourself

VN: aha

Participant: say for example in an office or in a school environment as a teacher, or in a lab is a chemical engineer or anywhere for any work or a doctor in a clinic, in a hospital, in a surgery. Any working environment That's going, it's going to have a stressful impact. Any working environment Is going to have a stress impact on you. Stress is not for everybody. But I can see that I cannot cope with stress properly in a matured manner. I get irritated. I just want to leave it aside, I don't want to do it. If someone asks me to do something I just do it a little bit and then I'm tired and then I want to go jump to the next thing to do. or like it's I do incomplete things certain times. There is no time for me to study because when I sit at the table I'm distracted, I'm totally distracted. Because I... everything along me seems to be Interesting other than the book.

VN: hm

Participant: And I wasn't always like that I was very studious. But unfortunately, with medication and adequate treatment I still don't see such a big difference. Because I still don't have the concentration anymore. And I still am fighting it on my own, it doesn't seem to be helping so much anymore.

VN: When you say anymore was there a point where you feel like it was helping?

Participant: There was a point where I was seeing head of department doctor from this clinic Where I felt it was helping.

VN: aha

Participant: And I was confident enough to child from [name] clinic. But eventually I feel like I Can't Make It Anymore because I don't know what I'm doing I don't know if there's a purpose in my life anymore. Because I don't seem to be focused on things that I need to focus on, so it's difficult

VN: Okay you are very brilliant

Participant: thank you

VN: and intelligent. And at least you are very aware about what's up. So, can you suggest to me because like I said before What do you think should have been done differently? What would you wish should have been done differently? What would you suggest for someone who's had the experience that you've had?

Participant: I would wish for them to be in control over their thinking and over Their emotions. And not let a thought distract you and not let anything distract you. you should Be a humble grateful human being. Like I am humble and grateful it's just that I have problems in me that are

affecting my behavior. I have these negative issues or like problems in my mind that affects my behavior.

VN: But you cope right?

Participant: I do cope

VN: how?

Participant: I cope by writing

VN: what makes you feel better?

Participant: makes me feel better? is writing. What makes me feel better is writing down in this little book here

VN: okay

Participant: Writing down every day notes of gratitude, And prayer, and summary of the secret by Rhonda Brine. And reading and praying and just praising God. It helps that really helps, going through prayer going to the temple it really helps. But at certain times I feel at sometimes I drift away I don't want to do anything. But I push myself to go there. I push myself to go there because If my little sister is to have the same problem that I have It's not that I would be ashamed of it I wouldn't be ashamed of it. I would tell her that she needs to be a strong fighter. She needs to tell the truth and she needs to not hold onto things from the past. My issue is holding onto things from the past right now

VN: what do you hold onto, things like what?

Participant: Such as things that happened in the past that didn't go well in the past. such as my schooling career

VN: aha

Participant: I had a lot of issues when I was in school going Society. So, trouble me, it troubles me that I couldn't you know make it in the mainstream School Like every day waking up at 5 or 6 And being at school by 7:30 or Being downstairs at home by 7:00 So I can be in school by 25 past or 7:30. I don't have that routine anymore. It's hard I don't like it. But I want to make a change if I become a teacher or a beauty therapist or whatever it is I would love to live another life just, just normal and on adequate treatment even if It means being an adequate treatment for the rest of my life. I wouldn't be ashamed I wouldn't mind because treatment is there to help. It's there to guide

VN: aha

Participant: People always told me at school, teachers always told me at school Don't take a lot of medication. That's what irritated me because they didn't understand my condition, my sickness. At that time, I was not diagnosed with schizo but if I go back to talk to my teachers They would definitely understand. because...They were understanding in a way but they were not really hands-on when it came to handling me in a proper way. So that was hard

VN: Oh, so you think you wish that your teachers had known better?

Participant: Yes, I wish that my teachers would have known better

VN: Ok

Participant: because not everybody is aware of mental health. Not everybody is aware of schizo bipolar and other mental health illnesses. They're not aware of it so I would like to, When I'm finished with school I'd love to write a book about mental illnesses. I would like to, I'd love to make people aware of these things

VN: aha

Participant: Because it's not something to be ashamed of at the end of the day. If you have a problem, if you have a problem like speaking to yourself, Or the anxiety or panic attacks you can come to the clinic it will help.

VN: So, come to the clinic is one of the things that you know that makes life a little more bearable to you?

Participant: yes. Coming here and speaking to my doctor's Every month or every time I have a problem at home, it helps. it does help in a way.

VN: So what kind of problems do you experience at home?

Participant: I experience very uhm Different problems such as I hear, I misunderstand, I misinterpret situation, a situation. Like if I misunderstand the situation it's hard for me to understand what's going on because I get confused between the reality and non-reality. What's happening in my mind and what's happening around me with people Are different, are two different things.

VN: Thank you so much for sharing is there anything you would like me to know? You have a beautiful story to tell. I understand that it's painful it's traumatic but I see a lot of positive in it

Participant: yes

VN: You're going to be a teacher or a beauty therapist You are just going to do this

Participant: yes

VN: you're looking forward to your... matric. So, there are a lot of beams

Participant: beams yes

VN: And you write beautifully

Participant: thank you

VN: And you are so organized

Participant: thank you

VN: Because you have all your books here and it's just lovely. So, you're very strong

Participant: yes

VN: You do know that?

Participant: yes

VN: Okay is there anything else that you would like to share with me? Anything that you want to advise me

Participant: uhm...

VN: Because like I said I would like to learn as much as possible. we want the intervention That we're to develop to be as inclusive as possible

Participant: Just be understanding with patients, if you become a doctor that treats **Participant**s. Be very understanding and try to go in depth about their family issues

VN: Sorry I try to go what?

Participant: in depth

VN: ok

Participant: And ask the family questions about their lives, about what happened. just be very understanding and very hands-on. Grasp whatever information you can

VN: hm

Participant: That's all I can say

VN: thank you so much

Participant: Thank you so much

VN: Ok

Transcription for QTS06

VN: Ok so as I have mentioned before that the things we are interested in Is how people cope with difficult situations. So, you have to, so I'm going to ask you to please tell me about the difficulties that you have experienced in your life

Patient: Difficulties I've experienced in my life?

VN: yes

Patient: Like I had a hard time talking to my parents with certain stuff I was going through

VN: aha

Patient: Like drug use. I found it hard to tell them everything I was faced with and what was going on. The things that my friends were saying. You find that I found it hard talking to them about that

VN: aha

Patient: Like some things used to be about my parents. you find that It's about my parents, stuff, negative stuff my friends were saying because my father is a bus driver. They used to tease me about that they'd say "your dad is this and this. He's not such a nice guy like he's not successful Because he's a bus driver"

VN: aha

Patient: So those are the things that traumatized me. they hurt my soul

VN: aha

Patient: So, I found it hard talking to them about those stuff, With my parents. but sometimes I would tell my mother because my mother is the one I'm closer to. So, I used to tell my mother some of the stuff and She'd be like "don't mind them they're just being boys or what" yeah.

VN: So, your friends would say things about you and your parents and you found it hard to speak to your parents?

Patient: yes ma'am

VN: ok

Patient: So, between the two things what did you find most difficult?

VN: Was It talking to your parents, communicating things that are worrying you? Was it that drugs thing that you mentioned or the most difficult thing was the friends Who were being horrible to you?

Patient: Yeah like both I would say. Talking to them and Friends were being horrible at the same time

VN: Ok when you say you had a hard time talking to your parents, how old were you when you realized that you were having these problems? communicating

Patient: I was like 18

VN: aha

Patient: I started facing problems like communicating with them on a deeper level, as I usually talk to them.

VN: aha

Patient: yeah, I was 18 Because that's when I started smoking a lot. And I wasn't, I was hardly at home most of the time. So, I spent less times with them and more time with friends. So, I Couldn't tell them anything that was going on in my life. So, I felt like there is certain stuff I can't share with them

VN: aha

Patient: So that made me like not talk too much about the stuff that I was facing so Yeah

VN: So, you were saying that you were not spending a lot of time at home

Patient: yeah

VN: So, you're not spending time because you were not able to talk to your parents or what came first?

Patient: I was like in a stage where I was ly...it was fun playing with friends. I found it fun being around my friends Instead of being around my family a lot. So most of the time I spent sitting with my friends at my friend's house or at the park playing.

VN: aha

Patient: or wherever at the corner store smoking

VN: So, what were you smoking?

Patient: I was smoking weed

VN: aha

Patient: and cigarettes, yeah. Those are the two things I was smoking basically weed

VN: And you wanted to tell your parents about this?

Patient: I found it hard telling them about it. Eventually they found out about it because I was doing it for a long time and my body started to show

VN: your?

Patient: body started to show the signs that I'm smoking. Like my lips become blacker, I was losing weight, yeah. so those signs started to show so that's how they found out, That I wasn't myself.

VN: And how was that for you?

Patient: it was kind of a rough time, Because I couldn't engage with them on a deeper level that I used to before I started smoking. So, it was kind of hard for me to talk to them about certain stuff that I was feeling or certain stuff that I was going through at that time.

VN: aha

Patient: yeah

VN: Do you know what, you're saying that before you were able to talk to your parents?

Patient: aha

VN: Okay so what changed?

Patient: Like my lifestyle changed

VN: aha

Patient: I started not doing the same stuff. I became a different, it's like I became a different person

VN: What staff were you doing before?

Patient: Before I used to help out in the household. Like I used to help my dad with anything he was doing in the household. Maybe like fixing cupboards or stuff like that

VN: aha

Patient: I used to help him out with doing the yard, I'd help him out. like watering the plants, I'd help him out. So, all of that stopped once I started smoking.

VN: aha

Patient: and I started spending more time with the friends, and less time home

VN: So, you had a breakdown in communication

Patient: Yes ma'am

VN: with your parents because you started spending more time with your friends. What made you want to stop engaging with your parents like you say? Why did that happen, why do you think that happened?

Patient: I felt more connected to my friends at that time than my parents

VN: aha

Patient: so, which just Stopped me engaging with my parents. Because I felt like this stuff I would talk about they wouldn't understand. And the age groups that we were in I just feel like they wouldn’t get it what I was going through

VN: aha

Patient: Yeah, I just felt like they wouldn't get it Because the age group that We are in.

VN: and Was there any change at home, did your parents have any change or are you the one who just decided that... like what happened at that stage?

Patient: there was change because they tried to help me out in my drug use issues

VN: aha

Patient: They tried to send me to rehab multiple times, for me to become better.

VN: aha

Patient: But sometimes I’d refuse to go. So, the matter ended up becoming worse, yeah.

VN: So, what do you mean worse, which matter?

Patient: Like my drug use

VN: aha

Patient: ended up becoming worse because I refused to go to rehab

VN: Ok so you're saying you experienced that difficulty. So, can you explain to me the difficulty was it the drug use? Or was it the breakdown of the relationship with the parents or was at the space that you found yourself in?

Patient: The difficulty was during the break down with the parents

VN: Ok

Patient: and the drug use

VN: aha

Patient: Because I felt like certain things I couldn't do it no more. I couldn't do certain stuff

VN: Like what, what kind of stuff?

Patient: Like communicating with my parents.

VN: hm

Patient: I felt like that stopped. I couldn't communicate with them like I used to communicate with them, previously

VN: Ok you say that your friends have said some stuff, how old were you at the time when the Friends were saying Things to you?

Patient: uhh Like I was 19-20 when they started making fun of my dad

VN: aha. Was that after your communication with your parents had problems?

Patient: yeah, that was after my communication with my parents had problems yeah

VN: which friends are those? Are they the same friends that you were always with, going out with?

Patient: they were different friends, different friends

VN: aha

Patient: because, I made a lot of friends and some friends couldn't accept what my dad used to do

VN: aha

Patient: Yeah so, they used to make fun of it

VN: how did that make you feel?

Nurse: sorry I just need this file. Oh! [name] is going for OT
***VN**: now?*
Nurse: no, it's fine you can carry on with your meeting
***VN**: Can we just carry on? We're almost done*
Nurse: yes, if you don't mind
***Patient**: I don't mind*

VN: Ok so some friends of yours could not accept that your dad was a bus driver?

Patient: Yes ma'am

VN: how did you feel about that?

Patient: I felt hurt

VN: aha

Patient: I felt hurt and like I felt like he's trying and people can't acknowledge that he's working really hard

VN: aha

Patient: Yeah, I felt like ashamed. I also felt ashamed about it for a long period of time

VN: what were you feeling ashamed about?

Patient: that what people are saying about him. like it made me, it made me look at him as if he's not doing enough but at the same time I knew how much he's putting in, how much work he's putting in.

VN: hm

Patient: so, like my friends made me have that perception about him in a way but like I used to love him. I still love him for doing what he did. and being a bus driver, I didn't like judging that much. now like that stigma was always in my mind that certain people were saying certain stuff about him. So, it was something that's always there, I couldn't Shake It Away.

VN: aha

Patient: Yeah but at the same time I respect him for being the man that he is.

VN: So, you were feeling this shame about what the people were saying And yet you loved your dad and you understood why, that he was hard-working?

Patient: Yes man

VN: how did that feel, make you feel or how did you react? How did you react when they said the things that they said?

Patient: I was angry

VN: aha

Patient: Because I remember this other time I was at a party, and This guy just talked about my dad and the work he was doing. So, his dad Passed away. so, he felt like he had rights to talk about my dad and belittle him because of the work he's doing. So, I felt angry I almost fought with him

VN: aha you almost?

Patient: fought with the guy, because I was being protective over my family.

VN: hm. And then uhm So you came to a near fight with this guy?

Patient: yes ma'am

VN: And how did you feel a few days afterwards? After the incident

Patient: I felt...

VN: How did it make you feel that you almost had a fight with someone?

Patient: I felt awful a bit. Cause I'm not a guy who likes to fight and starts fight and stuff like that. But at the same time, I was proud of myself because I stood up for my family

VN: Okay and how did you...so ok You speak about this feeling that you had about this relationship with your family and your parents, so thinking back, looking back how did it make you feel?

Patient: The feeling about, not having a deep connection with him?

VN: yes

Patient: uh It made me feel awful Because I couldn't express myself the way I wish I could at that time. And I feel like it held me back for sometime

VN: aha

Patient: Because like if I was connected with them from the get-go I feel like emotionally I would be far, I'd be ahead.

VN: So, you had this feeling it was holding, it held you back

Patient: yes ma'am

VN: and so, when people go through difficult situations sometimes they change the way they do things or sometimes the way they see life. Have you ever noticed that in other people?

Patient: yes ma'am

VN: aha

Patient: People change Comparing to what they're going through, depending on what they're going through

VN: How did the change of the relationship with your parents change you? And the way you see things?

Patient: it started to make me more...like I started to losing respect

VN: for?

Patient: elders. Like in a way

VN: aha

Patient: but at the same time, I respect the Elders but like I just lost a certain respect for my family members. Because I even started talking different towards them

VN: aha

Patient: I wasn't talking with the much respect, I lost respect

VN: so, when you say you lost respect you mean that you were not talking to them?

Patient: in the appropriate manner

VN: Oh

Patient: that as a child should be talking to their parents

VN: Why do you think you changed the way you were speaking to them?

Patient: It is part of like the old friends' stuff. Because as friends sometimes we look at how another person treats their friends, their family members. Then sometimes you practice the same thing in our household meanwhile it's wrong

VN: So, were your friends doing the same thing?

Patient: Yes, some of them were doing the same thing, because we were kind of living in the same lifestyle. As friends

VN: ok but you, you also told me that's not the way you used to do things before

Patient: Yes ma'am

VN: and now you changed

Patient: I changed

VN: ok and how does all of the experience that you just explained to me, how has it changed the way you go about your life?

Patient: It's changed me a lot because I've learnt to respect elders. And to respect humans and nature no matter how old or how small. But I've learnt to respect everybody

VN: aha

Patient: And to be able to communicate with people on a Decent level on like at a mature level to respect people and communicate with them in respect

VN: Ok so initially you were respectful and then you had a group of friends and You stopped being respectful

Patient: Yes ma'am

VN: and how did you come back now, to realizing that you want to be a different person

Patient: like my parents made me realize that I wasn't being, I wasn't at my best behavior. So, they told me I mustn't practice other people's stuff. They're living in their family. The stuff that they were doing in their family I shouldn't practice in our household. I should practice what we practice at our household, so I become the son that they want me to become

VN: was that, did your parents tell you that one day and then you changed or was it a process?

Patient: It was a process

VN: but what eventually made you realize that what your parents were saying was true?

Patient: like there's small stuff, like respect and talking to elders. Like it made me realize that I should respect everybody and every age group and talk to them in a manner which is respectful. So, I will get the same respect back, that I deserve

VN: aha

Patient: So yeah it was through a process, that took a while for me to get the hang of. Being right

VN: So sometimes, so how did you get to be admitted? Sometimes when people get an illness they have to be forced into admission. Were you forced into admission at any point?

Patient: Yeah sometimes I felt like I was forced

VN: aha

Patient: But like at the same time I realized that I needed the help. So, it was kind of like I was raised in time when I was trying to deny it

VN: So that was not a traumatic experience for you?

Patient: no Like I felt some type of ways though. I was going through some processes like I started not acting the same. I started hearing voices (*unclear*) And I felt like people are trying to communicate with me through social media, through TV. I felt like people are trying to communicate with me

VN: aha

Patient: Like I was basically hearing voices which are not there.

VN: Was that the thing that changed or was it just...what made you realize that you need to be admitted?

Patient: What made me realize? But like My parents realized that I needed to be admitted. Because I saw it as a norm. I was just scrolling through my social media platforms. I'm looking at this guy's (*unclear*) like it's a norm. Meanwhile I wasn't my best.

VN: OK so what do you think helps people cope when they go through stressful events like yours?

Patient: Like I would say medication helped me. My medication helps me a lot

VN: aha

Patient: Because it calms me down. And as soon as I took my medication I stopped hearing voices

VN: aha

Patient: Yeah, I stopped hearing voices

VN: So, you are saying that what helped you cope

Patient: Yes

VN: a big part of it was medication?

Patient: Yes ma'am

VN: OK anything else?

Patient: And being around people who are positive with life. People who are always preaching the word of god, they also helped me realize that anything is possible. That there is good in this world. And stuff that I believe in like when I believe certain stuff it can actually come true if I actually believe and pray towards

VN: where did you find positive people? who were these people

Patient: I found some of them here in hospital

VN: are they staff members, or are they patients?

Patient: mixed both staff members and patients

VN: Ok

Patient: yes ma'am

VN: And where else were you finding this support and positive people?

Patient: At home with the family

VN: Is there anything you wish could have been done to save you from this situation? That you found yourself in

Patient: Not necessarily something I would think about but like yeah

VN: So, you can't think of anything that could have been done to prevent This from happening to you?

Patient: no

VN: is there any advice you would give to someone else to prevent it from happening to them?

Patient: Like if you're given medication, stick to the medication that you're given. So, you won't relax and start doing the same stuff again

VN: hm have you had a few relapses?

Patient: Yes ma'am

VN: So, what was the driving factor behind those?

Patient: The relapse?

VN: aha

Patient: Oh, like the pills were prescribed under my father's name that I was taking previously. So, I felt like they weren't prescribed for me. So, I just threw them away and stopped taking them

VN: How were the pills prescribed for your…?

Patient: they were written in my father's name

VN: why?

Patient: Because I didn't have medical aid

VN: Oh ok. Is there anything else that you would like to share with me? Any difficult thing or suggestion

Patient: hmm Nothing I can think of. you covered almost everything

VN: any advice, like I said I am talking to you because we also need advice on what to do, how to include in our interventions that we are creating

Patient: my advice was like if a patient has been issued with medication, the patient should see throughout the medication that they take it correctly

VN: aha

Patient: And not relapse. because once you start your medication that's where you start to get sick again

VN: Okay what is it that you suggest prevents patients from stopping?

Patient: like?

VN: you are saying patients must not stop taking their medication, so they don't relapse

Patient: Yes ma'am

VN: So, what do you think keeps patients taking medication?

Patient: Like the health like their health being

VN: So, what helps them to keep taking it and not stop?

Patient: Like feeling healthy and feeling positive and seeing the purpose in life. that helps the **Patient**s to keep on going taking their medication

VN: okay so why do patients feel healthy and then take their medication?

Patient: Because medication helps with the mind

VN: aha

Patient: Like it makes us able to function well

VN: aha

Patient: Yes, ma'am it makes us able to realize stuff in an ordinary way. As an ordinary person would realize it

VN: Okay and do you have anything else you want to add, that you'd want us to know? Ok

Patient: (unclear)

VN: ok

Transcription for interview QTS07

Participant: I've been here for too long not being discharged. I'm old enough it's not as if I'm going to be Getting out of hand of an elder. I should be discharged I've been here for too long

VN: aha

Participant: I've been here for too long and what's more painful is that my clothes are not here. They’re elsewhere, where? in P1

VN: aha

Participant: Yes my things are not here they are in P1. It's not right that I'm situated in one place and my things elsewhere

VN: Ok so you've said that you've been traumatized because you were locked inside the house?

Participant: yes I was locked inside the house

VN: Who locked you?

Participant: I don't know who locked the house

VN: Who did you live with inside the house?

Participant: I was living with 6 girls from home, sisters

VN: Are they older or younger than you?

Participant: they are older but not that much

VN: So they just left and locked?

Participant: They locked the house and I’d come back and it's locked. I'd always come back and it's already locked

VN: They locked you inside or outside the house?

Participant: They locked me outside that's why I'm even here. I was staying outside

VN: hm

Participant: I haven't gone to the house. I have never gone. I last went to the neighbors when I had just come back to see them. I'd just come to see them, from the other side [location] and [location]. I stayed at the [location] because that's where I was working. And then I came back to see them and then I came to this side.

VN: hm

Participant: The house was locked again. I'm going to come to this side to look for jobs this side there's a garage here by [location], across. That's where I stayed as a person going to look for employment there and I came across people who were understanding. So I stayed there for a while, working, and was able to have some food. Yeah, my sister what I need you to help me with is by helping me to go back. Or there's no going back?

VN: you want to go back to where your sisters were locking you out?

Participant: yes. Just going back there and getting a pass out so I can leave. Because no one else is going to discharge me you see? Because I was admitted here by colleagues

VN: Who brought you here?

Participant: the people from my job. At the garage here

VN: hm

Participant: The mister from there, the boss. He said I must get in the car when I was coming, he said I must get in the car and I asked him where I'm going and he said I must get in the car. I thought it was people who were going to drop me along the way somewhere somewhere. And then I saw them bringing me here

VN: hm what did, what did...

Participant: But now I'm disturbed. I get disturbed because I don't know when I'm going to leave as I'm here

VN: What traumatizes you the most, is it being brought here and staying here or is it being locked outside of the house?

Participant: it's this one that upsets me, the one of staying here. It is the same because...Both of them are the same maybe it is better if I go back to breaking down the door at least.

VN: So when you're discharged from here you're going to break down a door where you live?

Participant: I'm saying it's better Breaking the door and doing that so I can get inside Or staying just a little and calling the neighbors If [name] is there. If I find her I'll ask her, I will ask for the key. If not then I will try to negotiate with the neighbors and see what I can do

VN: hm

Participant: Or I can call dad and he would come over to this side. That would be sitting outside as well you see?

VN: aha

Participant: yes

VN: Why do you think they lock you out, why do they lock you outside?

Participant: The last time I checked that house was blocking it was blocking. The toilet was full

VN: full of what?

Participant: the toilet was full of crap from the house, the poo.

VN: So why did they lock you out?

Participant: I think that's why they locked me out

VN: oh okay. So when you came and it was locked what did you do? When you arrived and its locked?

Participant: I don't know where to sleep. Because When I go to a friend's place they just tell me to go home knowing well that there's a sewage problem. They tell me to go back to the house when I get to the house it's locked. And then I wouldn't know where to sleep in the morning. Maybe around 7-8 in the evening. Then I would start moving to the bottom, to the bottom house. I stay in [location] do you know [location]?

VN: No I don't know [location]. How far is [location]?

Participant: here in [location]

VN: Oh okay I know [location].

Participant: Yes the double houses. Then I would go down to the container and stay there until there is light outside. Then I would wake up, as I'm saying, as I'm saying I don't have anything to say, it is hard you see

VN: What hardship are you feeling?

Participant: It's hard as I'm saying. Even as I'm staying here it's hard because now I'm here but I don't know when I'm going to leave. I don't know who's going to look after me you see. You can assist me by discharging me from here and be able to go back to that house

VN: the one they lock you out of?

Participant: Yes

VN: but how are you going to get in if you get there and it's locked?

Participant: I'll look around for the child from home who do they call her [name]. I'll look for her if she's there I'll ask about the key. And she will open up if they have returned the key. Because if I keep staying here nobody is going to fetch me because I was put in by people from work. I was not put here by People from home, as I'm here with this illness

VN: do people from home know that you are here?

Participant: huh?

VN: Do people from home know that you are here?

Participant: I haven't spoken to them, on the phone.

VN: you spoke to them?

Participant: I don't know, I'm not sure. I don't know

VN: They don't know you're here?

Participant: no I haven't spoken to them.

VN: You haven't spoken to them?

Participant: yes

VN: oh okay. Do you know their phone numbers?

Participant: No I don't. And I have become too mentally disturbed you see

VN: What got you too disturbed?

Participant: it's thinking about these things

VN: aha. So before they locked you out how were things at home?

Participant: I think the house was still blocked. The toilet sewage was still blocked. That when you sat on the toilet seat the flushing water would not come out. If you tried to flush the water it just rose up and never moved down

VN: Ok

Participant: that's why they locked, that's how I viewed it. Then they locked for a long time. That is what led me to come here, to the hospital

VN: so you came on your own?

Participant: Yes I ended up going away because when you sleep outside often you end up going away. You see

VN: So your trauma from being locked out made you see life in what way? How did it change your life?

Participant: I change my life really well because sometimes I'm not being naughty you see. I was not stealing you see, I wasn't doing anything. I just kept on trying to change it you see. Illness takes anybody who is by the door and throws them elsewhere

VN: So what did you do were you smoking?

Participant: yes cigarette, I smoke cigarettes

VN: Which cigarettes?

Participant: just cigarettes normal cigarettes.

VN: Not the green cigarette?

Participant: huh? I could no longer afford the green cigarette. I don't have money anymore to get it. Even for food

VN: oh but you've used it previously?

Participant: even to get the cigarette just cigarettes. I used that cigarette when I had money, not always

VN: okay. So right now what do you want to happen?

Participant: I want you to take me there to the house. To check if it's open because now

VN: if it's not open what are you going to do?

Participant: Then we'll just go to the neighbors and my sister's friends [name] [name]. Then we'll ask where the parents are. And then there are other girl children from home and we can ask them about the key. Because the reason I'm here today is that it's been a while sleeping outside, as I have arrived here. My spirit is going to end up leaving me

VN: aha

Participant: because I'm not settled here. I'm not settled

VN: Oh okay

Participant: I'm not settled so you could help me in that way

VN: So whenever you're rested you always think about that?

Participant: yes it's upsetting my mind because now the boys who put me inside the car were not supposed to lock me up and bring me here

VN: hm

Participant: It would have been better if they locked me up because I did something because they are not people who should have taken me to the hospital without being ill. They should've asked where I was going. They didn't ask me, I thought

VN: was it colleagues or random people you bumped into on the street or was it the police?

Participant: police, it was police, they came to the yard and made conversation. Yes the law keepers

VN: aha

Participant: because I also didn't know what they were talking about. They were talking and I just came to clean

VN: talking on which yard?

Participant: the same yard

VN: which one? The one you stay in?

Participant: yes I went there to clean. I was cleaning on the yard. I was sweeping and cleaning

VN: then they took you?

Participant: yes they came at that time while the conversation was going. And then the old man, the boss called me, some old man and said I must get in. I asked where I was going then they said no lets go. Here's money, they took out R20 and said here's R20 lets go. Then I asked where I was going, you see

VN: aha

Participant: they said no get in lets go, you see. Then I thought maybe I'm going to fetch something, now you see

VN: aha

Participant: and that's how I rode. When I had ridden that's how I stayed, till this day you see. That's I how I stayed till today, now I want to go back home. What they're doing here I don't get it, what they work with.

VN: what do they do here?

Participant: I mean the way they work, I don't see it

VN: what are they doing?

Participant: searching me and asking how I am today, if I don't have anything, any problems or if I don't have any problem. I have not been okay, I wish there was a social worker that can take me home, to check whether it's open or not

VN: they said they were going to get you a social worker?

Participant: no

VN: oh

Participant: I want you to help me get back home

VN: okay I'll speak to your doctor and tell them what your problem is, about what's not treating you well

Participant: it's going home

VN: okay

Participant: because I'm kept here illegally

VN: hm

Participant: they shouldn't have brought me here

Transcription for QTS08

VN: we'll start now so uhm as I have said to you what we want to find out is hearing from people what is upsetting them in their lives. So can you tell me what things have I upset you and traumatized you in your life?

Participant: Uhm it's being left by my girlfriend

VN: aha

Participant: Because I don't have any money. That really upset me and I began being ill and started going to hospitals. So the help that I need is just finding her so that my things will be better.

VN: hm Ok so you have also spoken about your mother

Participant: Yeah

VN: what happened to your mother?

Participant: My mother passed away in [year]

VN: aha

Participant: She passed away because of stress. She was very stressed

VN: aha

Participant: That's how she died

VN: Ok what else do you think of? That you can currently remember

Participant: What kind?

VN: that is upsetting you is there any that comes to mind at the moment?

Participant: No there isn't

VN: Okay so between these two things which one was more traumatic to you?

Participant: It's the one of being left by the girlfriend

VN: Can you tell me everything about being left by your girlfriend? What was happening what happened? What took place?

Participant: She just said on the phone that we must break up

VN: aha

Participant: Because I can't stand

VN: aha

Participant: it just became that

VN: hm she was your girlfriend for how long? Just tell me about her everything about her

Participant: I met her in [year]

VN: aha

Participant: When she was visiting her mother because she is adopted.

VN: Who is adopted?

Participant: this girl. She is adopted by white people. So her mother lives in [township]. So she was coming to see her mother in [township]. That's where I started seeing her. That's where we began exchanging numbers, and we contacted each other and it was all good. After some time... when I went to her school to look for her, they showed her to me and they called her parents the white ones. They called them and then they called her too. And they showed her to me.

VN: Who? Who was going to show her to you?

Participant: The ones from her school, her teachers. They showed me her and then she left. And that was that

VN: hm. So were you in love with this girl?

Participant: aha

VN: And then you went to her school?

Participant: I went to her school

VN: and then her parents were called?

Participant: Her parents yes

VN: Her adoptive parents, the white ones?

Participant: aha

VN: And you were also called?

Participant: yeah

VN: And then she was called?

Participant: They also called her

VN: oh and what was going on there? What was discussed?

Participant: they asked if she knows me and she admitted she knew me

VN: aha

Participant: And then she was told to go back to class and then they told me it's alright now

VN: aha

Participant: And that's how I last saw her

VN: was that the last time you saw her?

Participant: that was the last, Yeah

VN: ok and then what happened?

Participant: after that then I began

VN: began what?

Participant: being ill

VN: so what upset you about this girlfriend story?

Participant: that she left me. She left me when I still loved her

VN: So you were in love for how long?

Participant: from [year] to [year]

VN: Did you see each other and call each other?

Participant: Yes

VN: Ok and when did she call to tell you that you can't be her boyfriend?

Participant: When did she last say that?

VN: when did she say it, was it after the meeting?

Participant: no No

VN: when did she say it?

Participant: she said it before that.

VN: Okay so you went to her school after she told you you're no longer her boyfriend?

Participant: aha

VN: Oh ok. Why did you go to her school?

Participant: I wanted to see her and speak to her. Because I don't know her home. She doesn't want to let me know where she stays. She's scared of her mother and the others

VN: hm. Ok so after you were called and told you should leave, how did you feel?

Participant: I didn't feel good but I was happy to see her. That she was fine

VN: And then what happened?

Participant: After that then they said I was crazy

VN: Who said you were crazy?

Participant: People in the community saying I'm acting in an abnormal way

VN: Who?

Participant: people in the community

VN: In which community?

Participant: in [place]

VN: Why do they say you were mentally ill?

Participant: I also don't know. They just said it's as if I am crazy they don't understand me anymore

VN: And how did you feel about that?

Participant: That thing also upset me

VN: hm. So this girlfriend thing, when she told you you were no longer a couple how did you feel? How did you feel?

Participant: ey I felt... I felt another way that I cannot describe. Because when I tried begging her she just blocked me on WhatsApp. So that thing really upset me and then it ended up looking like I was really mad. Because I even went straight to her mother because I felt like that was the only option I had.

VN: So what did you do?

Participant: to go to her mother, the one who lives in [location]. And I asked her to Ask for her number. And then her mother said she will try but she'd have to speak to her first. And then that's how it remained. After that, I started visiting hospitals

VN: So you started going to hospitals after you were dumped by this girl?

Participant: yes

VN: Your girlfriend?

Participant: yes

VN: So who else knew that you had a relationship?

Participant: My friends from [location]

VN: Did her friends know?

Participant: ...

VN: The girl's friends. Did they know about you?

Participant: They knew me over the phone

VN: Did you know them?

Participant: I only know one of them

VN: Ok so after sometime how did you react to what happened to you? Your girlfriend breaking up with you?

Participant: What?

VN: after sometime how did you feel? Were you worried?

Participant: Yes

VN: so it was as if you really got disturbed?

Participant: Yes I got disturbed

VN: How did you know that you were disturbed? Were you doing it to yourself, what made you really see that you are disturbed?

Participant: I was running away from home I wanted to...

VN: You wanted? You were running, you were running from home?

Participant: yes

VN: aha where were you running to?

Participant: I have run to the road

VN: aha

Participant: I went from the road to back home. When I went back home, when I went home they welcomed me back. But I didn't want to stay there I just kept running away.

VN: what made you run..?

Participant: I didn't want their food I thought they were bewitching me. I wanted to start my own business. I kept wanting to start my business and wanted to get money to do that on the road. Then I tried then they caught me and brought me to hospital

VN: Who caught you?

Participant: father

VN: your father?

Participant: yes

VN: Ok so when you say you ran to the road what do you mean? Were you just running up the road or…?

Participant: I went to live on the streets

VN: And what did you do living on the streets?

Participant: I was a Street Kid

VN: pardon?

Participant: I was a Street Kid

VN: Oh you were a Street Kid?

Participant: yes

VN: what made you become a Street kid?

Participant: It's because I thought at home they were bewitching me. I thought they didn't want me

VN: aha

Participant: yeah so after that, I realized that no It's better if I go and source some money to start my business, and be able to have money. Because what I realize is that she found, this girl, found another one who's got money. That's what I think

VN: hmmm

Participant: So it's I was at a disadvantage because I didn't have money and I'm coming from a Township. But this girl loves me, it's just that she's scared of her parents that she could bring someone like me.

VN: What kind of person is a person like you?

Participant: Somebody from a Township. And I don't live this life that...I don't have money. So I think she was scared of all that

VN: So when you think about it you think that this girl loved you?

Participant: Yes

VN: but you didn't have money?

Participant: yes

VN: so how did that make you feel, how do you feel?

Participant: I feel like Starting up a business that's going to make me money. So that I'll have her back

VN: So how were you able to cope with the incident that took place? What did you do because you're still here even now? So what things do you think helped you to go on with life until this point?

Participant: It's by them taking me to the hospital.

VN: pardon?

Participant: It's by bringing me to the hospital. It helped a lot

VN: How was it helpful?

Participant: My mind is slowly coming back. I was also hopeless. I started doing things in a way I didn't know

VN: uhm How? You started doing things in a way you didn't know how? What were you doing that you think you were not doing right?

Participant: The one of running away from home. Because I was scared they were bewitching me

VN: hm

Participant: And living on the streets. I never thought I would live on the streets, yeah.

VN: Ok so before you broke up with your girlfriend, how were you as a person? What kind of person were you?

Participant: I was someone who loved rugby. I played rugby at [location]. I really enjoyed the rugby. So that was it

VN: so you were somebody who enjoyed the rugby?

Participant: yes

VN: when, before you broke up with your girlfriend?

Participant: yes

VN: And then when you had broken up?

Participant: Then I began one thing... I saw it best to quit rugby and start the business.

VN: hm. So did your behavior change?

Participant: hmm...

VN: The way you do things, did it change after your girlfriend left you?

Participant: Yes it changed.

VN: It changed?

Participant: yes

VN: ok how?

Participant: After she left me?

VN: aha

Participant: hmm it just changed. By quitting rugby, and running away from home that's it

VN: So is there anything you think could have prevented this thing? Right now you are here right?

Participant: yes

VN: You were mentally disturbed

Participant: yes

VN: So what do you wish should have happened? So that you don't get sick

Participant: It's by reuniting me with her and helping me speak to her

VN: So if she feels the relationship should end what would you have done for yourself so that you'll be okay in life?

Participant: I didn't get that

VN: I'm saying if she wants the relationship to end, if you have a girl you love, if you have a girl who loves you and then you didn't want her anymore Is it ok for her to be with you when she doesn't want you? When you don't want her?

Participant: No

VN: It's not okay?

Participant: yes

VN: The girl didn't want to be with you anymore right?

Participant: aha

VN: But you still wanted to be with her?

Participant: yes

VN: would it be ok then, to continue and have a relationship when she no longer wants to?

Participant: The problem is that I know that it's not because she didn't want to. It's not that she doesn't want me it's because she…

VN: why?

Participant: hmm She doesn't want me to find her easily

VN: When you say she doesn't want you to find her easily what do you mean?

Participant: Like she doesn't want to, she doesn't want to be an easy person like that, she wants me to work hard so I could find her

VN: oh ok. So when somebody finds themselves in your situation with a girl they love, but doesn't want to be found easily, what would be your advice? How would you advise that person what would they have to do if they were in a situation like yours?

Participant: It's by being resilient. They must be resilient

VN: aha

Participant: And continue trying until they win

VN: Okay so you still have hope that you’re going to win with this girl?

Participant: Yes

VN: when? When you get discharged?

Participant: Yes when I'm discharged from here

VN: endure, how are you going to be endure? What are you going do to show your endurance?

Participant: Then even when it's hard I won't give up

VN: So what happened that made you end up in hospital? Did you give up or what happened?

Participant: What?

VN: What happened that led you here to hospitalization?

Participant: dad brought me because they were saying they were saying I'm crazy

VN: So somebody who had the same incident as you, what would they have to do so that they won't appear as crazy? What should they do?

Participant: hmm Even if they see that they're crazy they shouldn't care about them

VN: The person should not care about them?

Participant: yes the ones who call him crazy

VN: What should they do?

Participant: he shouldn't care about them and continue doing his own thing

VN: Ok that's what you think would be helpful to them?

Participant: yes

VN: Ok so, in the end, you have survived, you are here. what helped you become right? What do you think helped you because you're fine now? Isn't it you still here?

Participant: yes

VN: you're fine and you're carrying on? What helped you continue after this traumatic incident of having your loving girlfriend…what enabled you to continue with life?

Participant: What made what?

VN: What helped you carry on with life?

Participant: Since she left me?

VN: even though your girlfriend left you, you're still here Going strong

Participant: yes

VN: so what gave you the strength to continue?

Participant: It's because I didn't give up because she left me and I want to take my life. I didn't do that

VN: What made you not give up? What gave you the strength to Know that you will not give up?

Participant: It's because I know she loves me

VN: Okay so you can continue because you know she loves you?

Participant: Yes

VN: ok is there anything else you'd like to tell me or advice for people in the same situation as yours?

Participant: hmm all I can say is that they should be resilient. Life is… even though you can tell that life is getting tough you should hang on

VN: what gives somebody strength to continue being resilient? What do you think it is?

Participant: it's being hopeful

VN: hmmm hope for what?

Participant: for the thing that they want

VN: That they would get it?

Participant: yes

VN: oh okay I understand. Is there anything you'd like to tell me?

Participant: there isn't

VN: That you would like to talk about?

VN: Tell me about the difficulties that you experienced in your life in general.

Participant: yes uhm first of all, uh I lost my mother at...at my youngest age I was doing uh grade two

VN: aha

Participant: I was in Primary. And he was sick mentally he believed that somebody was witch crafting her and so that led into my grandma, mother of my mum, uh to take me away. There were family conflicts and stuff, to take me away to [location]. And then we stayed there but I couldn't cope staying there because you know there were too...

VN: How were they?

Participant: there were too...how can I explain this? They were too strict yeah so

VN: So whose family was that?

Participant: My grandmother's

VN: Oh ok

Participant: so it was so difficult that, when my mother passed I went to school by walking. I would walk to school straight up. And every time I did something wrong I knew I would get a beating, a strong beating you know.

VN: hm

Participant: So, when you're still young that affects you because you end up not understanding...you end up feeling like everybody is trying to attack you and then that just builds up, as you grow old it builds up, because you don't deal with it.

VN: Okay so you mentioned that your mother passed away

Participant: yes

VN: Your mother had a mental illness?

Participant: yes

VN: And you were abused, you be were beaten up

Participant: Yes

VN: as a child

Participant: yes

VN: Ok of the three you have mentioned, which is...which one do you think is the worst? That you feel like you can't...

Participant: ey it was the beating because in beating I even confessed that ey I want to know my father's side. No one has ever provided me with my father's whereabouts

VN: hm

Participant: That made me long for my father, you know, because when you get a strong beating you become stressed. You know, you become worried you become afraid, that when am I going to get the next beating. Because they, they didn't have time to relax with me, talk to me as a child so I would get a strong beating

VN: Ok can you give me an example of the worst beating that you received. What happened, the atmosphere, what led to the beating, how were you beaten and why were you beaten?

Participant: you know I…

VN: where were you at the time?

Participant: I was sent to buy bread in the shops and I took some of the money (*laughs*) I bought sweets after that I realised that the bread is not at the shop and I went back home so (*laughs*) at home they asked me "where is the bread", you know there's no bread and the money is short now. So they asked me "how long have you been doing this, how long have you been robbing us our money" you know. And then I get, I got a strong beating after that and there was a time when they buy...they bought me a pair of shoes, *toughees*, and they expected they expected it to last for the whole year. So by [month] it was broken down and my teacher at school, the primary school offered to buy me a pair of shoes. And they said "no why do you buy, why do you bought him a pair of shoes, he should learn a lesson by walking with his feet uh in the in school so that next year he may know that wasting his shoes means that he will" you know...It was something like that so I felt horrible staying there. That lead me to even saying that I want to come back to [location]. Then I came back to [location] and then everything was in line but still those traumatic experiences keep on coming back coming back as I grow up it kept coming back.

VN: Okay so you say you were beat up for using bread money?

Participant: yes

VN: What did they use to beat you up? What were these beatings?

Participant: They used to stick of uhm...the Tree of peaches

VN: Peachtree

Participant: yeah

VN: oh ok

Participant: they would beat me with it

VN: and how was that? How did it make you feel, were you injured?

Participant: No I wasn't injured but I had bruises, I had bruises in my arm. I would go to school with bruises. And the way they were they would influence my teachers to beat me again, you know, because sometimes I didn't think of going back home. I would tell my friend and try to avoid going home, tell my friend and we would play and play and play. When it's time to go back home ey it was going to be tough because I know that they would expect me, on a regular basis they would expect me to sit under a tree shade just sit there for the whole day. As a child I would like to play some activities and stuff, so I would sit there just for the whole day

VN: So you were not allowed to play?

Participant: yeah I was allowed just to sit

VN: And how old were you when you were living there?

Participant: I was going to be, I was 12 years because I was going to, I came to do grade 7 here by [location]

VN: How long were you in that place? How long did you live in [location]?

Participant: From grade 1 up to grade 6

VN: Oh it was a long time?

Participant: yes

VN: okay. And how often were these beatings?

Participant: Eish twice a month. I would get beatings

VN: aha

Participant: Twice a month I would get a beating. And sometimes I confessed that I want to go to my father's side and I got a beating for that. I got a beating from my aunt and I got a beating from my grandmother. And even now I try because I understand that some people they are so... they're so into... (*unclear*) they are so they are so in the past. So they have traditional measures that they raise us with. So my grandmother when I say uh I want to meet her even now she would say, she would say I would be in danger and stuff

VN: if you say you want to meet your father?

Participant: If I say I want to meet her just to talk

VN: Your father?

Participant: no my grandmother

VN: Oh!

Participant: I no longer talk to her

VN: ohh

Participant: Even now

VN: ohh. She says who's in danger?

Participant: Me. she believes in witchcraft and stuff so

VN: Oh okay. And so the beatings, before you got beat, how did you feel when you knew you were going to get a hiding? How did you feel because you knew it was coming?

Participant: I was afraid

VN: aha

Participant: I was afraid most of the time

VN: How did you react?

Participant: You know I was almost afraid because when I just came when I just came to [location] they were always saying that I panic when I'm doing something, so I was usually this somebody who was usually panicking.

VN: aha

Participant: Even on normal days, "do this" and I'm normally like you know I'm always panicking. I would touch many things at once, you know. So I was like that and it took me time to get over it but still. At some point, when I'm in a… when I'm in panic attacks, it comes back. But sometimes it's those traumatic experiences… when they come back, they overwhelm me

VN: okay. So you get panic attacks?

Participant: yeah sometimes

VN: like what? Like when? Tell me about the panic attacks (*tissue*)

Participant: Like when somebody is who's older than me maybe when they tried to direct me on something I would always panic because I was used to when I did wrong, I was used to getting beaten up

VN: So you always expect that now?

Participant: yes

VN: hmmm. So how do you think the beating has, I know you've touched on it a few times, so if you were to answer that question how do you think the beatings made you become the way you are? How do you think they made you become?

Participant: because in the beginning of my sickness I thought everybody was out to harm me you know. So getting most of the beating psychologically made me feel like most people want to harm me. I don't know how it happens psychologically but sometimes I'd feel like that when my sickness began I felt like oh everybody is out to harm me somebody is going to cause me to suffer. Only to find out that the people are not going to harm me, it's all in my head.

VN: Oh ok. And then uh so how did you cope because you're studying you're doing good with your life you are in tertiary…

Participant: It's medication and then I took some few books in psychology, like the books that talk about positivity, how to overcome things, I have those books. I read those books and some of those books recommend that you take medication, take your medication uh that's why I read those books. And here in hospital I also, I also I spoke to a psychologist but I never spoke about the issue. But we spoke about medication and stuff, so she told me she reassured me that I must take medication, the situation will be better. And I'm hoping that if I take my medication I'm going to be alright

VN: Ok so you're saying you didn't talk about the issue with the psychologist?

Participant: yeah

VN: Which issue are you referring to?

Participant: That I have uh that I have been abused in my past

VN: Oh you never told the psychologist that when you were growing up you got a lot of beatings

Participant: yeah

VN: Okay what did you talk about with the psychologist?

Participant: I talk about medication, about my sickness

VN: aha

Participant: That when did it start and...

VN: ok. So now you said earlier that you sometimes think about it, you think about the beatings.

Participant: Yes

VN: can you describe to me how that feels? What do you mean when you say you think about it?

Participant: You know when I think about it You know when I think about it… it's causing me a memory of hate.. Those memories of hate hung in me because sometimes I thought about it and my sickness got back and...

VN: your sickness?

Participant: my sickness began to overpower me and then at home they say no it's because you don't take medication regularly. But sometimes it's those traumatic experiences, when they come back they overwhelm me

VN: So even if you're taking the medication

Participant: yes

VN: Sometimes when you think about it and you're overwhelmed then you start to get ill?

Participant: yes

VN: What symptoms of your illness do you get when?

Participant: You know, I think it's being afraid, being afraid of nothing but I feel inside that I'm afraid. I just don’t want to go anywhere because I'm afraid. You know, it feels like I can stay alone, do a lot of things alone, and social withdrawal and stuff so

VN: How often do you get that?

Participant: ey I try to avoid that now. But I used to get that. Now I am I'm back to normal, people's person and stuff

VN: Ok so sometimes when something bad happens to people, it just changes them and they become different.

Participant: yes

VN: have you noticed that about yourself? Do you think it is made you a different person?

Participant: I'm not as bold you know, I'm not as bold as I should be. Because even at work in [month] I'm working at [location], [location]. In [month] I had so many challenges customers shouting at me and I felt this overwhelming past coming back to me. You know I felt like quitting I even told the manager no no no I just want to quit. And the manager spoke to my guardian and calmed the situation down, and then after that I realised that I was being overcome by the past you know. When somebody shouts at me I feel that

VN: Okay and how do you react?

Participant: I just become dumb and I feel like going away and hiding, I feel like going away when I experience a lot of shouting

VN: aha, so you feel like it makes you withdraw?

Participant: Yeah

VN: And it has affected you at work?

Participant: yes

VN: in the way in which you respond to...

Participant: to the customers

VN: hmm. And then on the day-to-day basis has it affected how you do things?

Participant: no

VN: it has not affected the way you can do things? And so at work you ended up staying?

Participant: Yeah I ended up staying

VN: What gave you the strength to carry on?

Participant: uh I want to have a bright future you know

VN: aha

Participant: I want...I don't want what happened to me to happen to my children. So I'm trying by all means to learn everything I can learn. Because my sickness must be caused by psychological abuse or emotional abuse or sometimes as I said my mother was also sick. It can be something genetically, if it's genetic if it's genetically. I'm trying by all means to learn as much as I can learn, so that I can be able to help somebody who's in the position that I'm in

VN: hmm. So what are the things that are important to you that you think uhm can be done to prevent people from the consequences that you had?

Participant: can you explain please, can you explain please? And to get professional help like going to see...to get...when things get out of hand you can see a psychologist or a Psychiatrist speak about your issue and get other people's point of view about your issue. It helps you (*unclear*)

VN: Okay. And then when you said keep busy what do you mean?

Participant: like I'm always working out gyming I'm always busy so when I'm busy I don't get time to think about, and when I come back from the gym I'm always tired I don't have time to think about this. These depressing moments I don't have time to think about them. So that sustains me for now. But when I stop gyming and stop doing these activities, you know I stay alone a lot. And these memories come back and haunt me

VN: Ok so when you have these memories is it something like a nightmare or flashbacks how do these memories come? Can you just give me a picture of how it happens?

Participant: It's like something that plays on television but it's in your mind. You have those flashbacks when you were a kid being hit by… hit by your grandmother. Getting beaten. You have those memories, and also that the memory that your mother died. All these things come back together and cause my mood to be… to be bad

VN: okay by bad do you mean when your mood is bad? It can be many different things because some people when they're in a bad mood they're just angry. So for you what do you mean when that it makes your mood bad?

Participant: I become sad

VN: ok

Participant: it would take time to respond when somebody calls me you know. Maybe somebody is calling me [name] it would take few minutes to respond because I'm thinking and this thought is staying in my mind

VN: So it distracts you

Participant: yeah

VN: from your present situation

Participant: yes

VN: Oh you hear, you are aware of what's happening around you but you don't respond?

Participant: Yes

VN: oh okay. So if you were to suggest something to help people in similar (*unclear*) what would you say? What is your recommendation?

Participant: (*silent*)

VN: If people would say...because for instance like I said we're trying to come up with an intervention, something to help people who have schizophrenia and who also have had traumatic experiences. What kind of things do you think need to happen? For us in (*unclear*) what advice do you want to give me to help other people based on what you have experienced?

Participant: I think if we can if we can, just for a motivation if we can have, you know because when you are sick you have this thing when you come to a Psychiatric hospital you stay together with people who are, who are psychotic from bed you know. And when you stay with them your mental your mental your psychology becomes like ey I'm just like these people I'm not going to be anything in life you know. So, what I think could help us is to get those, those who cope better with our illnesses and form like sort of a support group and have them sharing their experiences with us how did they take the medication, how the medication has helped them. And how do they maintain a healthy lifestyle diets and stuff

VN: hmm

Participant: Yeah if we can have that I think most people will benefit from it and start to change their life. Because what I think is it's what we believe in because when we stay together these people it takes a lot of time because

VN: So when you say you stay together you mean in the waiting room or being admitted?

Participant: in the waiting room and in admission. Because I've noticed it's been 2 years now since I no longer go to admission. And my doctor advised me because when you get admitted your your psychiatric illness, when it meets influence, people who are psychotic it gets stronger, you know. When you withdraw yourself from them trying to take your medication and stuff and staying with people who are normal your psychology and your influence becomes like them. You start behaving like them it's taken me three years, from [year] I was doing my N3 in [year] I was supposed to do my N4

VN: aha

Participant: So at the time I was afraid that my sickness might come back at school so I withdrew I withdrew studying. And it took me two years to go back to school because I didn't have the confidence to go to school because I thought I was going to fail. I thought I was just going to be Lunatic at school. So if you can get a medication from people who because I've seen some working from [company] and stuff

VN: You have?

Participant: I've seen some people who are sickness, on the phones at [company] big companies so definitely that means that if they can take medication and become alright they can do whatever goal that they need to do in life.

VN: Ok tell me do, I know that you were young, about your mother's illness what exactly was she having? What was she experiencing, your mom now?

Participant: ey she thought that everybody is, you know my mum from her past I also think she got traumatic experiences because she, she would grow up all his life believing that her mother was, the sister of her mother...So when he got old he was told that no this is not your mother your mother is this one so after a while he got sick in the head. But I was young and I didn't...

VN: So who did she think was her mother?

Participant: My guardian my guardian the sister of her mother

VN: Oh she thought her aunt was her mother?

Participant: yes

VN: And she was told later on that this was actually her mother?

Participant: yes

VN: oh and you think that is what affected her?

Participant: yes

VN: And your grandmother? What exactly was her story because you also seem to think that she has some problem?

Participant: ey I don't know. I don't know about my grandmother

VN: hmm. What do you think about her mental health?

Participant: (*big sigh*) I think she's old but you know people from the past uh people from the past uh...how can I put it? The olden people used to go by respect. They greatly believed that... if somebody is doing something wrong they get beaten. You know get a real beating until you stop whatever they want you to stop, they're like those people

VN: ok so you don't think there was a problem with her except for the fact that it was the culture

Participant: yes

VN: of that time?

Participant: yeah

VN: Oh okay. Is there anything else that you would like to share with me?

Participant: I think I've shared everything

VN: Oh ok thank you very much for your time, we'll talk a little bit afterwards. Sorry.

QTS10

VN: what traumatic things have you been faced with in your life?
Participant: I was sad because the father of my child left me
VN: aha
Participant: and then I caught this disease
VN: ok so how did it take place?
Participant: it mentally abused me. My heart was really broken
VN: can you tell me everything about this…
Participant: it's just that I no longer know sister
VN: you don't know the story?
Participant: no
VN: of what happened?
Participant: no. my mother knows
VN: okay what was happening to you at that time?
Participant: I can't remember. It was long ago
VN: which year?
Participant: ey I don't remember
VN: hm so what did the child's father do?
Participant: he just left me
VN: how many children did you have?
Participant: I had 1 child
VN: okay was the child already born?
Participant: it was born
VN: and what did he leave you with? With HIV?
Participant: when I went to get tested they said I've got HIV. They said I was newly infected
VN: aha
Participant: and I said thank you for finding out that was the case. Then I sought assistance and they said they I'll start with medication
VN: and then what happened?
Participant: It's just that I don't remember doctor
VN: so did you go home and tell the child's father?
Participant: it's just that we haven't seen each other in these... We haven't seen one another
VN: so he didn't know? He left without knowing that you got tested?
Participant: no
VN: so he didn't know a thing about you getting tested? Was the child already delivered?
Participant: the child was born
VN: so when did you get tested? Before or after the child was born?
Participant: I tested when it was born
VN: oh okay. Had you been tested while pregnant?
Participant: yes
VN: what were the results at that time?
Participant: they were negative
VN: oh and then when the baby was born?

Participant: and then, then I was told I had HIV
VN: okay. Why do say it's what got you upset?
Participant: yes
VN: what happened that traumatized you?
Participant: it's just that I can't remember doctor, it's been so long
VN: okay and then illness for the treatment you're taking here, when did it begin?
Participant: I don't remember because even the card I have is new
VN: oh okay. So how do you feel about the matter that your child's father just left you like that?
Participant: I already took him out of my heart
VN: okay so that doesn't bother you anymore when you think about it?
Participant: no
VN: did it change the way you do things?
Participant: It comes and goes
VN: when it comes how do you feel?
Participant: my body feels tired
VN: aha, you feel physically tired?
Participant: yes
VN: when you think about that?
Participant: yes
VN: okay. So what do you do to cope with it? To continue with living your life?
Participant: oh I just forget
VN: what do you do?
Participant: I forget.
VN: do you make yourself forget, or are you able to not think about it on your own?
Participant: yes
VN: how do you do it?
Participant: I just dissociate and forget about him
VN: okay and then be able to continue with life as usual?
Participant: yes
VN: okay. So how old is the baby now?
Participant: they're doing grade 12 this year
VN: how old are they?
Participant: 17
VN: 17?
Participant: yes
VN: oh okay. And then…you say you can't remember what happened at that time?
Participant: no
VN: your mother knows?
Participant: yes
VN: okay when the baby's father left you…when you first began taking treatment from here did it begin before or after the child's father left you?
Participant: yes
VN: how long after he left you?
Participant: I don't know anymore

VN: okay. I understand ma. When you think about it you feel sad?
Participant: yes
VN: okay. Is there anything else you'd like to tell me?
Participant: …
VN: okay thank you very much ma

Transcription for QTS11

VN:…about what was said
Participant: oh okay
VN: so as I have said to you brother can you just explain the things that have traumatized you in your upbringing.
Participant: you see, you see in life sister I was disturbed from school, when I was on the 10th grade. What traumatized me is not being mentally healthy. That's what I can say.
VN: hmm
Participant: the way I saw it. Time went by and I was helped I don't know whether it was [year], I'm not sure because I left school in [year]
VN: you left school in [year]?
Participant: yes sis
VN: okay.
Participant: even then I was already unwell. You can see by the ID I created, you could tell this person was not well.
VN: hmm
Participant: my father and mother tried, my mother was also still alive, they tried again and I became fine again
VN: what did they do?
Participant: I went to [hospital name] [place]. But where I got treated, to keep it short we don't have to go far, because even in hospital I realised escaping was a challenge, I got treated in *[name of village]*, a [name] village. Another man there named *[name]* gave me traditional herbs because my condition required traditional intervention, and in that manner I was helped.
VN: okay
Participant: in the past year, I don't know what they saw because I was aware by all means, a boy from home came here, to [city], saying we must go to that man in [month]. And where did we go? We went home. At home I've got my own house now sister, I slept at my place. When I first got to my place I get there it's full of people, around 11. And I ask "what happened" they say "my wife is not well". With her being unwell I ask "what's wrong", they say "she's not okay mentally". When we were sleeping and people were gone, she says the reason she's not okay is because they said I was fooling around in [location], I'm going to get hit by cars. Meanwhile I'm coming from [city] from making a delivery. I came back on [day], on [day, date, month]. I signed papers, delivered and did everything and came back and parked the truck properly and we all signed off as people leaving work and was told to come on Monday. This thing took me because it said it knew how my life was. The thing that upset me even then was the family issue. I have a lot my sister I don't want lie
VN: hmm
Participant: my sisters, since the past year, they don't call me they don't say anything. When I started seeing I'm unwell I used to call them every time and say siblings please try and talk to me. I'm doing something and talk to your brother and say you want *XXXname*. We haven't talked from [year]. They even brought mom to [location]. I don't even know a pill doctor
VN: you don't even know what?

Participant: a pill. If I had to help someone, that's how they took me. In the morning sister, I'm explaining how I got home. I got discharged and went home, I wanted to hear this thing of being told I'm mad, where? By my dad. I got home and sat and tea was made and done, everything was done. Thereafter they said we should get inside the car, and I got in the car sister. And it was obvious we were headed somewhere. We went, I didn't know where we were going and we left my house. My wife was not being told anything, nothing was said. And we parked in [town] to some traditional healer there sister. I blew a pee right there sister. I was swollen in my privates

VN: so you were taken to a traditional healer?

Participant: yes sister, a black traditional healer

VN: and you arrived there and began being ill?

Participant: no sister. The thing that became problematic sister, which really disturbed me was when I tried, I asked them nicely and said whatever they were seeing I was asking them to release me and meet up with my children and tell me I'm coming back today. I can't use your tonics, because they did say my healing was going to be for 3 days.

VN: who, which ones?

Participant: the ones from [location] I was taken to, that were chosen by the family. Then it was evident they didn't agree.

VN: they didn't agree?

Participant: yes and I was telling them even my wife doesn't know as I was brought by my siblings. That was not accepted, what was happened was I was taken and put on chains until my tummy would swell. All that was delayed. What I don't know is how this report got to work because what I gather is this was stress plus depression

VN: you began having stress and depression after all this?

Participant: no after their process

VN: this thing okay. So you stayed for how many days there?

Participant: I think about 7 days

VN: didn't they say it would be 3 days?

Participant: they said it would be 3 days. I don't know how it became 7 days. And when they took me to my wife they said to my wife she shouldn't say to the doctor, since I'm scared of the doctor that they had me chained. I'm the one who gave permission to my wife and said take the child and go to your dad, to look for the police so you can come and fetch me from here. Otherwise I should have died last year

VN: okay. So your family took you away without your permission, without your wife's permission and took you to a traditional healer?

Participant: exactly sister

VN: who then chained you?

Participant: yes sister

VN: why did they take you there?

Participant: they said I'm rude.

VN: okay

Participant: I'm alert sister. I can tell you what was happening, as I'm telling I was coming from [location] after a delivery. And went to park a truck, I didn't crash any truck. The report that was discussed in the company, I don't know it.

VN: who made the report?
Participant: what I don't know is the boy from home, it's clear that he insinuated the report.
VN: what did they say at work?
Participant: I don't know what he said. Because it was said there should be brain scans and all. Saying I could possibly have a tumor. Because on the [date] a letter was written to go back to the doctor.
VN: by your work?
Participant: by work, yes. By [location]
VN: when you left there, where did you for 7 days?
Participant: I went to [hospital].
VN: hospital?
Participant: yes sister. Then after that I called them at home and told them I'm ready to work, they said no choose between [hospital] and [another hospital], the hospital I was attending. And then I [this hospital name] because when I was coming from Blink blats I take a straight taxi that leaves me close to here and then I chose it. I followed the [hospital] procedure, until the [date] date when I went back to work. Then it was clear I was still not welcome at work.
VN: what did they say you've got, at [this hospital], what's the problem?
Participant: you see sister I didn't even ask because it was like…
VN: which department attended to you?
Participant: its…I came just above here sister.
VN: to psychiatry?
Participant: yes
VN: oh so you went from your home to [hospital], then came here to Psychiatry?
Participant: yes sister and opened up a file here.
VN: okay
Participant: my wife was waiting for a date for schools to open in [month], I don't know the date on which schools opened. Then my wife was going back to school, and taking the child back to school. That's how they left me. They left me and I followed the procedure.
VN: you were not admitted here?
Participant: no I wasn't admitted. I wasn't
VN: so what did they say here? What were you given?
Participant: the pills I was using (unclear).
VN: okay
Participant: the pills I was using. Saying they're trying to decrease dosage I don't know whether it was too much injection from Holy cross that I had received, I don't know. But it must be something there, but the file has it I think it explains. But that's what they were saying.
VN: okay. So you view this experience as traumatic to your being?
Participant: no sister, you see I'm no longer shocked by illness. I'm being attacked by a dark person.
VN: how?
Participant: no sister when you're a grown up you get beaten up, and when things happen to you don't be surprised. I'm not surprised by something like this sister, I can say there was a lot of jealousy. Even within the company they were saying the truck vehicle I was using was not suitable for me, it was suitable for them, those who are driving it today.

VN: okay so when you say you grew up being beaten up, do you mean with a cane?
Participant: no
VN: how do you mean it?
Participant: I mean when you grow up poor, it doesn't mean you should be unwell
VN: so you're saying you grew up poor?
Participant: I'm talking about that from school they wanted to disturb me from my journey
VN: who?
Participant: I'm talking about black people sister
VN: oh
Participant: yes
VN: when you say black people are you referring to people who know you or people who…can you just clarify for me I want to be on the clear
Participant: you want clarity I hear you sister.
VN: yes
Participant: there are people who do witchcraft sister, to clarify sister
VN: okay. So there are people who practice witchcraft?
Participant: yes sister
VN: so they're the ones who did this?
Participant: they were trying their luck sister. This was a second time at an attempt
VN: at what? Making you sick?
Participant: aha
VN: oh okay. These people practicing witchcraft, are they alive or people who know you? Who are these people?
Participant: form the rural homes sister
VN: oh okay. You're saying these people were always there in your upbringing?
Participant: they were there sister. There are others showing up today that I was not aware of. My situation is tough sister, the family is involved truly speaking.
VN: so can you start from the very beginning? Isn't it you've talked about it towards the end where it ended up affecting your job and a lot of things? Where did this whole thing begin, if you still remember? What happened?
Participant: you see sister at this stage we're on now, it shouldn't have come to this stage
VN: hm
Participant: I should've been long working. But because of the dates stated by the doctor I'm still admitted. Because the doctor I met up with said I must wait until this date and not touch drugs. After the [date] date an [ethnicity] came after work to this hospital and said, I don't know what they said then it was clear I had to continue with treatment. I had to continue with treatment and I had no problem, I continued with treatment. But the stated date will come no matter where I am. Its painful sister having a home and not work
VN: so the painful thing is that you're not working?
Participant: it's very painful sister I don't want to lie, its crushing me. You see sister today I don't even have a bag.
VN: hm
Participant: can you tell me how you'd feel knowing your child doesn't have a schoolbag?

VN: it's painful, very painful. So the issue of jealous people, the witches from your members, when did it begin?
Participant: you see sister it began with me finding my wife, that's where it became clear. When I had a wife, when was it? From [year], it was nice finding a wife. In [year] it was nice, I don't know from [year] my mom was still alive.
VN: so your mother was still alive in [year]?
Participant: yes
VN: [number] years after you had a wife?
Participant: yes. [year], [year] … I don't know when it began. I think [year] is when it began. It became clear
VN: how did you notice that there's something?
Participant: it's the present family members, sister. The wife reports, but the sister could intervene, she'd call and say this is how things are.
VN: when the wife called, can you give an example of what she complain about.
Participant: you find that sometimes they say she doesn't listen, she goes out and what not. When I heard it from the uncles and asked them what was going in the homestead, I'd find that none of that is happening. Like this other time she asked to go home, I don't know whether there was a funeral at her home, my mom wasn't well, she asked to go home for burial right? On the funeral day my mom fell off the bed and my wife wasn't there at that time. When the story changed, she didn't stay for one day, and an aunt from Shepstone came and it was clear that my wife couldn't give my mom attention.
VN: so you're saying in [year] they started being angry at your wife?
Participant: the relationship was not good
VN: so in [year] they spoke badly about your wife, of untrue things?
Participant: aha
VN: where were you in [year]?
Participant: I was here in [location]
VN: oh you were working?
Participant: yes
VN: so they'd call you and speak badly of your wife?
Participant: no these things would come from the sides, until it was heard from my sister's house. I was told by my cousin. People would go around to tell the person who I get along with, my cousin. My cousin said to me "cousin why are you staying home? When you're this troubled" I said "how cousin?" He said "I've had enough cousin, you must make your own effort".
VN: oh when you got married you were staying at your home?
Participant: at home
VN: with your mother, wife and father?
Participant: yes
VN: oh okay. So the people on the side are they family members or villagers?
Participant: they're family, my own family. Unfortunately mother left them, it's like we didn't have a mother because we had our hopes on her. Till this day my father hasn't come to my house.
VN: hm so you're saying your mother was still alive, when did she pass away?
Participant: when did she die, in [year]? Yeah

VN: okay. So when you're thinking on your own about what… you've spoken about even while you were growing up, so these things began when you were grown and working or while you were young in primary?
Participant: no sister. I was beaten down when I was already in high school
VN: yes what happened?
Participant: as I've told you that I was beaten down still young, I was in high school you understand? I was unwell it was said to be Lifufunyane by people
VN: yes what was happening, what did you have?
Participant: what used to happen was you'd find that I am with a traditional healer, I'm unwell but I'd run away. And I'd want to go back home and at home they'd bring me back there.
VN: what gave them the impression that you needed traditional healing, you're unwell? What symptoms did they see?
Participant: symptoms, I don't know whether it was speaking or what because I'm always talking and joking. I can say it was something like that.
VN: so they usually took you to the traditional healer?
Participant: yes
VN: while you were in high school?
Participant: yes I think just when I left high school, I went straight to the traditional healer, and from there I was working and fine. Thereafter I did my license and then I found jobs and started working. Until the past year when it became clear I was ill.
VN: oh
Participant: meanwhile I could see with all eyes
VN: wait when you were in high school, what do you mean you could see with all eyes?
Participant: I'm speaking of the past year. I admit in the past years, but as for last year, when I was even taken off work, I don't agree with them. Even the Indian who had come here can show, *XXX name*, where he'd seen me crash his truck in the past year. Meanwhile he had given me such a nice truck
VN: oh what is your boss saying at work?
Participant: my boss refuses to take me back to work
VN: what is his reason?
Participant: he said he wants a second letter as evidence that I'm alright
VN: hmm
Participant: because he said I didn't mention the size of trucks I drive. When you drive a code 14 truck what comes to your mind sister?
VN: a big truck
Participant: yes. It means I drive heavy duty, that's how I can put it. Their issue sister, if I tell you the truth, they gave away the truck, so when I went back to work, fine, they didn't have a truck to give me. It's difficult now to give me an old truck, they were not truthful
VN: oh so the doctor gave you approval to go back to work?
Participant: yes, on [date]
VN: and at work what did they say? No position for you?
Participant: no they said they require a second letter
VN: oh. Where was the first letter coming from?
Participant: from here, [hospital]

VN: stating you can return to work?
Participant: yes
VN: and then the second one, what did it state?
Participant: I'm waiting for the second one with results from the brain scan. Here are the brain scan papers
VN: oh okay. So all that you've mentioned your wife, employment, which would you say is more traumatic?
Participant: you see sister I don't want to lie, if I can't go back to work I won't have anything.
VN: oh. So is the issue that the illness has separated you from work? Or what they're saying is separating from work?
Participant: you see sister I don't care about a lot of things. A person can speak anything about me, you understand, but if I have a means of living I won't have a problem.
VN: hm
Participant: and if I can drive a bantam or truck I can take my money and look for employment elsewhere. I don't have a problem with that
VN: hmm
Participant: because I don't have a problem looking for employment. Because the time I'm waiting, the hospital is wasting my time too much.
VN: what?
Participant: the hospital
VN: aha
Participant: it has wasted too much of my time.
VN: so you feel like the hospital has messed up, with regards to your job?
Participant: I can include it too, the date they wrote. When my boss came here, it changed the date
VN: so how does everything you have mentioned make you feel?
Participant: you see sister, I'm expecting one thing which is for the Indian to say "here is the key, drive my truck" that's all.
VN: since that is far from happening, how do you feel?
Participant: I don't have anything, I just gym. I don't have anything to do, I gym. I wake up in the morning and gym and during the day, just to pass time because time is running out for me. Looking at people go to work in the morning, and come back, a life I'm not used to.
VN: oh you've never remained without a job?
Participant: this thing has never happened. I started in this company in [year] [date], working for that company. But today I'm collecting crumbs, it shows that I'm nothing. My company has showed me sister
VN: so when you think about what has happened, has it changed the way you see life?
Participant: you see sister, what this thing has done for me, it has shown me that in life you don't have to be dependent on one thing, or it has opened my eyes. If I can find another opportunity now sister, I'll do a lot
VN: okay it didn't change your behavior?
Participant: no sister
VN: and the way you feel about yourself?
Participant: no sister it hasn't changed anything, I don't want to lie

VN: okay and the way you do things, has it changed?
Participant: no it hasn't changed sister
VN: okay. So every day you're still continuing with your life the way you were before?
Participant: as I used to be sister I don't want to lie
VN: oh okay. So another thing I want to as you now is finally you're okay, you're here
Participant: I'm alright I don't feel anything
VN: what gives you the strength to continue? What gave you that courage to wake up in the morning and carry on with your life?
Participant: it's the stress of looking at people
VN: which people?
Participant: those who go to work. What are you talking about?
VN: no what I'm saying is that in the end you were not hopeless. You didn't tshafa
Participant: hm
VN: you still carry on. What keeps you up daily, and enables you to go on with life?
Participant: you know sister sometimes you can see that you have Ixhala. I'm not worried about a job
VN: hm
Participant: a job is what I'm not worried about. Even if the word is 'this one is not ready to drive' I don't have a problem. Even if I sign to go back to work, I don't have a problem.
VN: hm
Participant: I can even call you tomorrow and be like 'sister what I was talking about there is not accurate'
VN: so what makes you cope, to survive this stress? Because there are big things you find yourself unable to provide for your family, that you could previously. So what gives you the strength? If you were to encourage someone else what would you say, or advise they do?
Participant: nothing much sister, but to take things that are not building and put them aside and look forward. Because if the mess behind, if you realise it in life, and stop focusing ahead you won't be able to move forward in life.
VN: oh so the thing that enables you to carry on with life is that you were focusing ahead?
Participant: ahead sister
VN: you don't look at the traumatic thing that happened?
Participant: no sister you won't be able to go on with life sister if you're like that.
VN: okay.
Participant: you wouldn't be able to carry on with life.
VN: so you'd give that advice to someone in your situation?
Participant: I'd give them that advice
VN: so your boss thinks there's a problem? And your family thinks there's a problem?
Participant: you see my family sister, I'm going to speak of my family I don't want to lie. They're very dirty back home
VN: hm
Participant: even if you think from last year sister, when [month] came it was even hard to come inside my house. I had built and finished a house by myself. It's just hard to come and look inside the house, you don't even to take a car, you just walk. Not even 10 minutes pass and you're already at my house

VN: so this thing of theirs not coming to your house, why do you think that is?
Participant: jealousy nothing else. It comes from the people you least expect. And then when I go there, I'm expected to go with them and not look after my own things, and lookout for the family. When I'd just begun building the house, and bought bricks they said (unclear) said this old man, I built 5 of them.
VN: oh so the relationships got ruined because you became independent?
Participant: that's how sister.
VN: okay so what I want to ask is that why did your boss also think you're not fit to go back to work, to drive?
Participant: he also thought that
VN: why did he think so?
Participant: I don't know sister. I could say it's the report they get, which I don't know what it said
VN: so you think your family lied about you to your workplace?
Participant: truly sister
VN: oh not that the boss has noticed some things? Or made comments?
Participant: he didn't make any comments because the report came from another lady. When I last left work they said "come on Monday, have a long weekend and come on Monday".
VN: so the boss didn't see anything?
Participant: he didn't notice anything
VN: oh okay. So you have placed all this on the family?
Participant: no sister as I'm not working today it's because of them. I have placed the burden on myself to a point where a boy from home is paying my rent. But paying rent for me in [place] is nothing because my people at home are hungry, I've got a wife and children.
VN: the doctors are giving you treatment for what?
Participant: I've explained it at the beginning that I don't know what it's for. But I was just following company protocol
VN: oh so if the company said follow up with the doctor you're going to do what they want?
Participant: yes
VN: so you'll get your job back?
Participant: yes. And to know my job is safe in case they accuse me I can take out papers and show them I was going to the doctors since.
VN: okay. So as you're home doesn't it happen that you think of this…and feel heartbroken when you think about your situation?
Participant: you see its painful sister for example when you see trucks parked on the screen like this
VN: like what?
Participant: I live at [location] (unclear) N2 is just across. When they load, the load just across me.
VN: so you constantly see the trucks?
Participant: yes it comes that I'm waiting for employment. But it doesn't upset me, I just keep on waiting for a date
VN: oh okay
Participant: it doesn't upset me

VN: don't you ever think about it when you're just resting?
Participant: no sister. I don't want to stress myself about something that's not going to help me sister
VN: oh okay. You make sure that you don't stress?
Participant: stress about something that's not going to help me? No. because when I'd just begun here I could tell I've got stress. I took my medical aid and went to pioneer
VN: you did what?
Participant: I took my medical aid and went to [bank name] bank. And I said I couldn't sleep, they gave me pills.
VN: when was that?
Participant: the past year
VN: from where?
Participant: it was in [month] and I was already using this hospital
VN: okay you'd already started your treatment here?
Participant: yes. I took the medication, the sleeping pill, and the one you take 1 hour before you sleep. And I was able to sleep. Before they got finished I stopped them because I didn't want to sleep only by taking pills. I stopped them and then slept thoroughly
VN: oh okay. Is there anything else you'd like to share with me?
Participant: no sister. I'd be messing around
VN: oh you've explained everything?
Participant: I'd be playing with you sister
VN: okay.

Transcript QTS12

VN: okay I'll just put it here right?
Participant: yes
VN: as I have already said, the things I want us to talk about today are about what had occurred in your life that traumatized you. What traumatic experiences have you been through?
Participant: as I am saying it's the smell
VN: okay can you speak up a little bit so we can hear each other.
Participant: it's the odour
VN: okay, it's a smell from what
Participant: it's just a smell from my body. I bath, brush my teeth but I just have a smell
VN: okay who has ever told you you've got odour?
Participant: I am just aware of it. In the past I used to smell it maybe when I'm asleep and covered with blanket. I used to smell it too perhaps when I was asleep and covered with a blanket.
VN: aha
Participant: I used to smell it
VN: okay
Participant: and as time went by I ended up not smelling it but I can tell it lasted because when I come people shut their noses.
VN: so you feel like you've got body odour?
Participant: yes
VN: okay it's coming from which place?
Participant: I also don't know.
VN: uh has anyone ever told you you've got a smell?
Participant: no nobody
VN: when you tell people you've got a smell what do they say?
Participant: they also get surprised when I talk to them.
VN: what do they do?
Participant: they are surprised, shocked
VN: oh when you say they are shocked you mean they don't pick it up?
Participant: they can't smell it
VN: but you can smell it?
Participant: I just think maybe it's a way of comforting me
VN: you think everybody is trying to comfort you?
Participant: yes
VN: okay when did this odour issue begin?
Participant: in [year]
VN: how old were you at that time?
Participant: I was 15

VN: okay. So how could you tell?
Participant: I could just see people block…or when I bumped into them blocking their noses
VN: okay, so you thought you had odour? Why does this bother you so much?
Participant: it's because when you meet up with other people you must also look like someone who takes care of himself.
VN: hm
Participant: yes. Because I do bath and brush my teeth but I can just tell that even when I'm speaking, sometimes I think it's in my mouth, but I get surprised because I brush my teeth.
VN: okay. And at home do they also tell you about this smell?
Participant: no they've never. I have spoken about it, I told my mother when she was still present. And she said she can't sense it.
VN: hm
Participant: and I also told those at home, the sisters
VN: okay
Participant: yes. I don't remember how they responded
VN: so you say your mother was still alive?
Participant: yeah
VN: what happened to your mother?
Participant: she passed away
VN: oh in which year?
Participant: in [year]
VN: oh okay. And when did the smell issue begin?
Participant: in [year]
VN: okay. What was wrong with your mother?
Participant: hm?
VN: what did she have, what happened to her?
Participant: she got sick and passed away
VN: was she ill for a long time?
Participant: no I think for about 2 weeks
VN: how did you feel about your mom's passing?
Participant: it was upsetting but I tried to accept it
VN: oh okay. So since you sense this smell do you hear voices or have other things?
Participant: I no longer hear the voices
VN: oh okay. Other people say that being sick, or hearing voices, or getting admitted ending up restrained, or just hearing voices upsets them
Participant: yes
VN: has that ever happened to you?
Participant: hearing voices?
VN: being upset about it, yes. About the voices
Participant: oh being upset about if there's someone speaking on the side?

VN: hm
Participant: no that's a normal thing, it happens when you're a human being to be upset. It happens, but it's a thing to pass. You have to move from it
VN: oh okay, so this smell bothers you. Is there anything else that has upset you?
Participant: it's just the same smell that bothers me because I seem as if I'm not like other people
VN: how?
Participant: because I'm different, like people look at me as if I don't love myself.
VN: people look at you as if you don't love yourself?
Participant: I think people will see me as someone who doesn't love myself if I'm going to come up smelling.
VN: hm
Participant: yes
VN: oh okay. So this smell issue, do you think about it all the time? Is it something that's always on your mind?
Participant: yes when I'm walking. Because it happens when we're sitting together with people you start seeing people block their noses. You see?
VN: hm
Participant: even when you open your mouth to speak, then you see them shutting their noses.
VN: aha how has it made you, this smell issue? Has it changed the way you do things?
Participant: it has made me feel pain because I even dropped out of school
VN: so you left school because of the smell?
Participant: yes
VN: what happened?
Participant: because I could tell that I was shy of going to school.
VN: so the odour changed your behaviour because you stopped attending school?
Participant: I stopped going to school
VN: what else has this smell issue changed in your life?
Participant: the bad impact was on school. Where I left school and fought. That had a huge impact because I left for 5 years, and only went back in [year]. When I gave in because I realised there was no difference, staying at home and not staying at home was the same. And returned, and let any person say whatever they wanted
VN: oh so in [year] you decided to go back to school?
Participant: yeah
VN: whether the smell is there
Participant: yes
VN: and the people will decide on their own?
Participant: yes
VN: oh okay. What else has the smell issue changed in your life?
Participant: it had an impact on that because now I was behind other learners.

VN: oh okay. When you first began noticing this smell, what was happening in your life? Is there anything else that was going on, or happening?
Participant: I was sick. It began when I had been sick
VN: you were sick with what?
Participant: I was just sick, even swelling on my private part. I was normally sick at that time.
VN: aha
Participant: yes
VN: so the smell began when you'd just been sick
Participant: yes I had been sick
VN: okay. What did the doctors say you had?
Participant: I was just consulting traditional healers often
VN: aha
Participant: because they suspected it was a traditional matter
VN: what made them see it as traditional, what was happening?
Participant: at that time I was getting swollen on my private parts. I don't remember clearly in [year], it's a while back. But I was sick, that's what I remember.
VN: oh okay
Participant: even taking a pee at that time was difficult.
VN: aha
Participant: yes. After a short while I found myself with the smell.
VN: oh from
Participant: from that time
VN: for the smell have you gone to a traditional healer? A witchdoctor
Participant: A witchdoctor?
VN: aha
Participant: I used to go to doctors too, way back. And report to them
VN: and what would the doctors say?
Participant: also the witchdoctors, I would tell them. There are many of them I would inform.
VN: and what would they say?
Participant: the traditional healer said "no you'll be fine" you see. But I didn't observe any change
VN: and the doctors? What did the doctors say?
Participant: it's just that this was a continuous issue
VN: so you said you'd also consult doctors for the smell issue, what would they say when they spoke to you?
Participant: I'm starting to forget but I did have a problem
VN: oh okay. When you think about the smell problem how did it make you feel?
Participant: painful. As I've said that I have this…I just feel down, you see. I become shy
VN: aha
Participant: yeah

VN: so what things are you no longer doing in your life, because of the smell?
Participant: it's just that. Mainly the school was interrupted because even when I went back, when I was studying matric they deregistered me.
VN: who deregistered you?
Participant: I was deregistered by the teacher
VN: why did the teacher deregister you?
Participant: he deregistered me, he said I don't submit my assignments on one of the subjects, business studies. And said I should choose another one to deregister from.
VN: so he said you don't submit?
Participant: the assignments
VN: aha. Did you submit the assignments?
Participant: I'd already submitted that assignment.
VN: you only submitted one?
Participant: I'd already submitted the assignment at that time
VN: he said you shouldn't submit?
Participant: he said you are not going to write, it's already [month]
VN: he said you submitted it late?
Participant: he said "it's too late, you won't write" so I ended not writing. That's what led me to not passing grade 12. They said they will…what did they say? There's this thing they come for, they said I can write it the following year
VN: and then what happened?
Participant: the following year it became difficult because I wasn't able to go back and sit on the desk, for just the 2 subjects. Because you'd find that the bus arrives in the morning takes you at 7, so you'll leave at 7am only to find that the subject is from 1-2pm for instance it's a last period.
VN: hmm
Participant: yeah
VN: so you ended up not going to school?
Participant: not going. I went to write in [month], when I got there they said maths was already written. I no longer went, I was no longer involved. When I wrote BS, the teacher no longer stood in front of me so that did disturb my life. You see?
VN: hm
Participant: that also disturbed it, it really upset me. It upset me because even now from time to time it comes to mind that I didn't pass matric but I also didn't fail it. Because the other subjects I got a chance to write I passed all of them. So I'm saying if I got the chance to wrote those I didn't write, that the teacher deregistered me from I probably would've passed.
VN: so when you say it interrupted your life, how do you mean?
Participant: it interrupted me because I would've made moves. I would've made ideas or plans if I had passed.
VN: plans such as what?

Participant: of moving forward
VN: and do what perhaps?
Participant: I loved social work
VN: okay. What happened when you didn't write the following year?
Participant: when I came they said maths was already written
VN: and the year following that what took place?
Participant: the following year I didn't follow up on that.
VN: oh okay so this issue…
Participant: because the statement came out, because I wrote one subject from those I did not write. The statement came just like the old one, just as the one from last year. With the exact same results like the one from the existing statement.
VN: what about the subject you wrote, what did they say about it?
Participant: the results for it were not available
VN: the results for what you wrote were not available?
Participant: yes
VN: oh so you quit?
Participant: then I quit
VN: okay when you think about what happened to you, how often do you think about it, do you think about a lot?
Participant: I think about it from time to time because those I was studying with have gone far. You see
VN: aha and how do you feel when you think?
Participant: that upsets me. If it were by me, it's just that I don't have money, I'd go to Saint James to fix my matric. But I still don't have money. The chance I had to do it for free, disappeared.
VN: oh okay. So you ended up like that?
Participant: that's how it ended
VN: hmm. So you feel like the issue of not writing and having odour affected your life?
Participant: it affected my life. Yes it did because now I realise the years have gone by you see.
VN: aha
Participant: I even think of doing security and driving taxis. Because I can see now that I won't be able to continue studying
VN: so on a daily basis, has it changed the way you do things? The fact that you didn't finish your 12th grade
Participant: yeah from time to time my mind comes to think about that, you see. But then it ends there, I try to swallow although it's bitter
VN: aha. So you say you swallow even though its bitter, what helps you to swallow?
Participant: I just accept because there's nothing I could do. There's no other option I can try to take

VN: and then what do you do? What wakes you up in the morning, which enables you to continue with life?
Participant: I just wake up. I think its God's power that I can wake up
VN: oh its god's power. If a person finds themselves in your situation, what would you say to encourage or advise them?
Participant: ey maybe what helps me is that I am God's child, I believe. I am encouraged by God's word. Yeah, because when all these things happen I look to God and pray. I pray and look to God to fulfil my wishes at that time.
VN: okay I understand you. Okay so moving forward now, what are you going to do?
Participant: I was thinking of two things. Doing my driver's licence, and doing security. Those are the two things I saw as a shortcut to getting me to succeed and making money that's to support my livelihood. That's what I was thinking of because now I can see that these problems, are problems I won't be able to sort out.
VN: is there anything else you want to tell me, that you'd like to talk about? Perhaps that happened to you?
Participant: no there isn't.
VN: oh okay. Thank you so much for your time.

Transcription for QTS13

VN: as I have already mentioned that our job is to help people to cope with difficult situations. What are the traumatic experiences that have happened to you, in your life? What happened to you?
Participant: the job was no longer there, so I don't continue with security.
VN: oh okay. Aha
Participant: if I didn't stop it, and carried on I think the income got raised a bit.
VN: okay. Can we please speak up a bit, so it'll be easy to hear here on the recorder. So what is the issue, the job?
Participant: yes. I left and the income behind got raised.
VN: aha
Participant: now I don't know how I am going to go back. To go back it, I still have to try.
VN: okay. Are you currently employed?
Participant: there's an informal job I got, on the road
VN: okay. But you're saying the problem is that you quit your job?
Participant: yes
VN: why did you leave it?
Participant: because there was an opportunity for the security learnership. Now I couldn't do both of it
VN: oh okay. Is there anything else that took place in your life that upset you?
Participant: no
VN: even in your upbringing?
Participant: it was just that I wasn't working
VN: okay. So what distressed you was just the job issue only?
Participant: I have forgotten other things.
VN: pardon?
Participant: I'm saying I can't recall other things.
VN: oh okay. Do you need some time to think? Or we should discuss the job issue?
Participant: we can talk about also that while I was studying I had pneumonia and didn't treat it, and I could've been helped at that time. And it wasn't easy to carry on I don't know what happened.
VN: you're saying you had pneumonia and didn't treat it?
Participant: yes
VN: and then what happened?
Participant: that's how it ended, when the problem was found.
VN: aha
Participant: and I didn't carry on. Now I keep thinking if I continued when it was found what I had.
VN: so they said you've got pneumonia?
Participant: yes. They said there was something on my chest when I did the x-ray.
VN: aha. When was that?
Participant: it was…many years have passed
VN: what did they say it was?

Participant: they said I must go the hospital and I didn't go. And it was troubling before I…it was troubling me, what I had
VN: aha. So then what happened when you didn't go to the hospital?
Participant: now I keep thinking that if I went to the hospital I would've been helped.
VN: so you still have pneumonia?
Participant: it's slowly coming to an end.
VN: pardon?
Participant: it is slowly coming to an end
VN: but it is still felt?
Participant: a bit.
VN: what do you feel?
Participant: I feel like the chest becomes tight.
VN: aha. Okay is there anything else you remember, that caused you to be upset?
Participant: it's just that I don't have matric
VN: so what happened with your matric?
Participant: it's just that I didn't get it and now it causes job opportunities to be less.
VN: okay. What caused you to not have matric?
Participant: I failed
VN: what grade did you fail?
Participant: grade 11
VN: oh okay. And then what happened, you didn't continue?
Participant: no I didn't
VN: why didn't you continue?
Participant: I didn't want to keep on failing. That I was going to fail again, and I was old at school. And didn't want to learn with youngsters
VN: how old were you?
Participant: I don't know how old I think I was…17
VN: you were old at the age of 17?
Participant: a little older than 17, around 19 perhaps
VN: oh okay. So you've told me about three things now. The issue of not taking care of your chest, and quitting your job and they give a raise.
Participant: yes
VN: and not completing grade 12. Which was most traumatic between these 3? Which was more traumatic?
Participant: that I didn't finish…it's the same but (unclear). Because also not following up with the hospital for treatment.
VN: which one are you saying is most traumatic?
Participant: that I didn't finish school.
VN: is that you didn't finish school?
Participant: yes
VN: oh okay. Why does it upset you so much that you didn't complete school?
Participant: because jobs are not easy to find.
VN: when you left school how did you feel?
Participant: when I left?

VN: aha
Participant: I didn't think I would have a problem when I have quit.
VN: when you think about this matter of leaving, how does it make you feel?
Participant: I feel as if I should've continued when I see others pass…working nicely
VN: how do you feel when you think about this?
Participant: it saddened me, that only if I had finished. If I'd only continued studying
VN: so does it happen that you think about this a lot?
Participant: yes
VN: and then when you think about it, do you think it has changed the way you do things now?
Participant: the way I think?
VN: yes
Participant: I try to hold on to whatever comes
VN: hold on to what?
Participant: jobs. Sometimes it gets better, sometimes not
VN: so you try to hold onto a job when you find one?
Participant: yes
VN: oh okay. So this issue of not finishing school and leaving a job, how has it changed your life?
Participant: uh it's just the stress of not finding a job, but I think of it and it passes. I think it of it and it passes
VN: oh okay. So are you still the same person you were in school? Or it hasn't changed the way you think of things?
Participant: no it hasn't changed. It comes to mind that if I'd only continued to study, even though I have grown, I could've tried to finish.
VN: okay. So the way you see things and life, is still the same as the time you were young and in school? As when you'd made that decision to quit?
Participant: no
VN: what has changed?
Participant: now I think when I can't find a proper job, I remember that I left school. I don't have matric. It's not the same as when I was young, when I was young it was better, I wasn't thinking. While I was still studying I didn't think much.
VN: so you're saying when you were young, you think of this matter in-depth, but now you are?
Participant: yes
VN: when you say you don't think of it frequently, how often do you think of it? Is it daily, weekly?
Participant: sometimes I forget it because… I think of it sometimes, and forget it and ignore it.
VN: oh okay. You think of it and forget it sometimes?
Participant: yes
VN: so in the end you're here, what keeps you going with life, when you have this problem? What pushes you to continue with your life?
Participant: it's the jobs I find
VN: oh okay. So you find jobs?
Participant: yes when I find a job it becomes better
VN: and when you don't have a job? Do you go for a long time without finding a job?

Participant: yes
VN: and what do you do? How do you carry on with life?
Participant: staying at home helps me, otherwise…
VN: oh family members help?
Participant: yes
VN: they help you?
Participant: yes they do grocery. They work and buy food for the home.
VN: okay. So being with family members here is what helps you?
Participant: yes
VN: okay. Is there anything else you'd like to tell me?
Participant: hmm we're done
VN: okay. Thank you so much for your time sister.

QTS14

Participant:
VN: can you tell me the difficult situations or things that traumatised you in your life. Can I put it here by you?
Participant: yes
VN: so, what things have happened to you and left you traumatised?
Participant: uh it's that I quickly finished, I stop going to school at a young age. I couldn't continue, because my peers are doing things, and succeeding in life and I couldn't. I also quickly began working, and I was working hard jobs, that were not paying much. I was also living alone and sometimes I'd run out of food, and at home my sister was not working. And that would force me to sleep without eating for several days. Yeah
VN: okay. Why did you have to leave school young?
Participant: it's because my mom passed away when I was 12 years old.
VN: okay
Participant: and I don't know my father. Then I stayed with my sister, she took me as her child.
VN: so, your sister, when your mom passed away how old was she?
Participant: she was 19
VN: oh okay.
Participant: yes, but she was already married. She got married early when she was 18
VN: okay can you tell me about this whole situation. About what was happening, what it was like.
Participant: it was hard because the family was living far in Bergville. And they're the family closest to me and my sister. But she became much closer to me because she took care of me and did everything. She clothed me, cooked and got food for me because I was living with her. Although she fought with her man because she'd been married twice. She married her first man when mom was still alive. Then mom passed away, we stayed with him and he used to hit her all the time. One night I woke up he'd placed a knife on her head threatening to stab her. It was storming and raining outside at night, she was calling my name and I woke up in the night when I tried to help her, she ran out, my sister. She ran to the neighbours. That passed, they forgave each other, and they fought again when we moved, when we found a government house, and we were out of [location] and in [location]. They also fought there, I woke up he placed a hammer on her head, he was hitting her with a hammer the one with things like this...
VN: he hit her with what?
Participant: with a hammer.
VN: a hammer?
Participant: yes
VN: hmm. What kind of thing is a *Sando?*
Participant: it's a hammer
VN: oh okay.
Participant: yes. He was hitting her with a hammer on the head. So, even then the neighbours stepped in. all that really traumatised me because I couldn't even cope in school because I was hearing my sister's screaming voice. As if now it's something that is still happening, meanwhile it already happened. He also received support from his mother because he knew we didn't have

parents. The husband's mother would sometimes favour him, send him, he would tell on my sister to the husband's mother, and she would take the husband's side. Because she knew we didn't have anybody it was me, my sister and the child only.
VN: hm
Participant: yes. That really traumatised me, but I don't forget because I was living with friends at school and I was able to talk and pass through it. But when I went home, I'd be fearful that this thing could happen again.
VN: so, was this thing happening all the time or?
Participant: it used to happen maybe twice a month. Twice every month, this other day he beat her up because he thought she was pregnant with someone else's baby meanwhile it was his. She gave birth to the baby and it looked exactly like him, when he thought it was someone else's and she was having an affair on him meanwhile that wasn't the case.
VN: how did he know the baby was his?
Participant: he thought it wasn't his baby. At the end it was his baby, it looked exactly like him.
VN: hmm
Participant: yes
VN: so how old were you at that time?
Participant: I was still at standard 7. I was probably around the ages of 17 and 18 somewhere there. I was 18
VN: you said when you were in grade you were 18?
Participant: yeah. That's when I quit school.
VN: okay so when he began this habit of hitting you, you were in what grade?
Participant: grade 8
VN: oh grade 8?
Participant: yes
VN: perhaps how old were you? 16?
Participant: yes, I was probably 16
VN: oh okay. So, it is something that used to happen, you say you used to think about it a lot?
Participant: yes. At school I couldn't cope well because my sister used to scream painfully. I was sleeping in the kitchen and they used to sleep in the bedroom. It was the kitchen, dining room, one bedroom and the toilet. It was a government house
VN: hm
Participant: yes, and I used to sleep in the kitchen, in the dining room floor. And they used to sleep on the bed with the baby, they still had one baby at that time. They fought and fought and kept fighting at night all the time.
VN: when you first heard of this thing, how did you react? What did you do?
Participant: I was shocked because I knew them as people who were loving to each other all the time and when I'd wake up in the middle of the night, they're fighting, insulting each other, even with a hammer on the head. That is what scared me the most because I thought he wanted to kill her. Or he'd kill her one day.
VN: so, were they both fighting, or your sister was trying to protect herself? Or was she also fighting?
Participant: she was being beaten; my sister was the one getting beaten.
VN: oh, he used to beat her?

Participant: yes, he'd beat her up
VN: and then what would you do because you were a child, you were small? What did you do in that situation?
Participant: I couldn't have said anything because we were also staying at his house you see.
VN: hm
Participant: we were staying at his house, this man, my sister's husband.
VN: so how did you feel about that?
Participant: I felt very traumatised too because I love my sister, it is not nice hearing her cry every night. Getting beaten up by someone she loves even.
VN: hm
Participant: yeah. It really traumatised me
VN: and then when it has passed, between getting beaten and was there anything else, or how did you feel?
Participant: I still was afraid that it would happen again
VN: okay. You say it usually took place about 2 times a month?
Participant: yes.
VN: so, between the 2 times
Participant: My sister also took pills and almost killed herself.
VN: hmm
Participant: yes. And she woke up in hospital
VN: really?
Participant: because of him, yes sister.
VN: hmm. So, between the time your sister got beaten, you remained afraid you say?
Participant: yes
VN: and what did you do between the times?
Participant: they wouldn't argue
VN: and how did you feel when they didn't argue?
Participant: I became comfortable when they don't fight but not completely because I was afraid that they would do it again. Because this is my sister, she is the only person I have in my life. I don't have anyone else in my life
VN: when you say you were afraid, how did it manifest; was your heart beating faster, can you describe to me how…?
Participant: I was just afraid. I was just afraid
VN: when you got scared, were you shaking, heart beating faster or couldn't breathe? Like what happened?
Participant: the heart would beat faster.
VN: aha. What else used to happen?
Participant: I had a lot of thoughts in my head that it could happen again. As they're happy like this, I wished for it to last forever. Because this thing of beating each other up damages me.
VN: was your heart beating faster?
Participant: it was beating rapidly.
VN: when you were thinking about it, or only when it was happening?
Participant: when it was happening or when I was thing thinking about it sister
VN: okay and breathing, was it painful to breathe?

Participant: no, it wasn't painful
VN: was it hard to breathe?
Participant: yes, it was breathable
VN: it wasn't hard to breathe but the heart was beating rapidly
Participant: yes
VN: that's all
Participant: yes because of fear.
VN: oh okay. So, the experience you had with your sister, you say you sister was married when your mother was still alive?
Participant: she got married when she was alive, when she had passed.
VN: the first time?
Participant: yes
VN: oh, not when the mother was still alive?
Participant: when she was still alive, they were dating they weren't married, but they'd planned to get married.
VN: when did you begin living with her?
Participant: since she got married, since mom died
VN: oh, they were living together and then they got married?
Participant: yes
VN: oh, you're saying they got married the first time?
Participant: yes
VN: and then what happened after that? Your sister became your mother, right?
Participant: yes
VN: and then what happened?
Participant: and then they lived a life, after that it forced me to move from them to rebuild my home. To rebuild the shack before it was a support house. I got there and the rains were pouring in. I didn't have sail, or iron to properly cover the roof. Even then I was suffering because sometimes I wouldn't get food. And she would send money, and sometimes wouldn't. because she doesn't work, work came and went.
VN: oh, so you left your sister?
Participant: yes
VN: who said you must leave her? What happened that caused you to leave?
Participant: uh I don't know. I think her man complained. Because she just said I should go back and live at home now. I think her man had complained.
VN: how did you feel when your sister chased you out?
Participant: I became sad because I was still young and wasn't used to living by myself. But I went home and lived by myself, and thereafter I was taken in by my aunt after some years.
VN: you were taken in by your aunt?
Participant: yes
VN: okay what your relationship with your aunt?
Participant: our aunt is mom's sister
VN: oh okay.
Participant: yes. I lived with her at [place] and I studied there, she also had her alcoholic husband. He bothered her, and always shouted at me for lies, and lied about me. When things

were said, he'd say he told me meanwhile he didn't. And sometimes sent me to the tavern for beers in the middle of the night, when he'd been to the bar the whole night. He'd send me for beer yeah
VN: how old were you at that time?
Participant: I think I was doing 8
VN: you were doing 8?
Participant: yes
VN: oh. When you lived with your sister
Participant: I lived with them twice
VN: in the same year?
Participant: no, I lived with them
VN: you lived with your sister first?
Participant: yes
VN: what grade were you doing, standard 8 or grade 8?
Participant: I was in grade 8
VN: when you were with your sister?
Participant: yes
VN: okay. You lived with your sister for how many years?
Participant: probably one year
VN: oh! With your sister?
Participant: yes
VN: and then you lived with your aunt?
Participant: with my aunt
VN: okay and what class, what grade were you doing?
Participant: I was doing grade 9
VN: okay you were doing grade 9 with your sister?
Participant: yes, when I was living with my aunt's I was doing grade 9. And lived and lived with her, and her man used to lie about me. That man didn't like me, he was an old man. . He'd send me for beers midnight when he'd been at the bar the whole day. When he got to the house, he'd send me for beers and shout at me even when there was no need.
VN: what was he shouting about, perhaps?
Participant: for instance, if my aunt left a message that something should be done, and he wouldn't tell me. When my aunt asks me why I didn't do that certain thing, he'd say he told me meanwhile he hadn't.
VN: something like what?
Participant: for example, they were building at home, they were making a garage because there was a staircase. So, tractors would come every day to fix, to detach and she said I must look out for a tractor and give the person money for their work when they're done working, and he wouldn't leave the money and he'd go and drink.
VN: so, your aunt would give him the money?
Participant: yes, yes.
VN: and he would leave the money?

Participant: he wouldn't leave the money behind; he'd go and drink with it. And my aunt would come back and ask me what I did with the money, why I didn't give the person their money for their work.
VN: and what would he say?
Participant: he'd say he gave it to me, he told me.
VN: and what did you do because you didn't have the money, how was the worker paid?
Participant: I used to get shouted at
VN: hmm
Participant: and it'd be as if I didn't give the money, I spent it, meanwhile I didn't use it. I'd be shouted, I lived under that pressure all the time, he'd lie on me. And place mistakes on me that weren't mine.
VN: hmm
Participant: yes.
VN: okay. So, when you were living at your aunt's and living with this man who wasn't good to you
Participant: yes
VN: when he spoke lies about you how did you feel?
Participant: I felt like I wasn't welcome there
VN: as what?
Participant: as if I wasn't welcome to live there. Because even the non-existent mistakes were used to blame me.
VN: was your aunt aware of that?
Participant: my aunt was always absent because she worked.
VN: and her husband?
Participant: her husband was not working. He only lived to drink alcohol that's all
VN: oh okay. And what happened after that?
Participant: after that I stayed and stayed and ended up being forced to leave, they also kicked me out.
VN: who kicked you out?
Participant: my aunt.
VN: what did the aunt say she was kicking you out for?
Participant: I think she was being annoyed by her man, this old man who was prompting her
VN: and where did you go?
Participant: I went to stay at [location] by myself for a week in the shack where I'd been living with my mother
VN: oh. You went back to live by yourself?
Participant: yes. Then I looked for a school there and I couldn't get in because they required a school fee of 1000. Then I couldn't get it because I first lived at my aunt's.
VN: you did grade 9 at your aunt's?
Participant: I was done with grade 9 at the end of the year. I went to [location] to do grade 10.
VN: and your aunt?
Participant: my aunt didn't have a problem with me, we lived nicely together.
VN: you lived with your aunt at your mom's shack?

Participant: no what can I say, I used to live at home, but I would eat there. Because she was close to mother
VN: so how was she your aunt?
Participant: she's an aunt because she's born of the Ndaba's. she has a child born from uncles at the Ndaba's
VN: oh, who is at the Ndaba's, your mom?
Participant: my mom is a Ndaba. Yes
VN: and your surname is also?
Participant: I use my mother's surname.
VN: oh okay. So how was it there?
Participant: it was nice, I lived with her children and there were no problems. But then my mom's government house was ready. Then then required me to stay in it, the one I live in now and renting out.
VN: so, you live in it now?
Participant: yes, I was living in it now before I became ill.
VN: so, from the things you have described which one do you think is most traumatic?
Participant: that I couldn't finish school.
VN: that's the most traumatic?
Participant: yes
VN: when you think about the issue of not finishing school what comes to you, internally?
Participant: I see myself as a failure, as a loser because I didn't finish school. Because maybe I also could've been far in life. I could've done many things in my life, smart things that could make my dreams come true. As a human being
VN: so sometimes when people experience traumatic things, it changes the way they do things or their behaviour or whatever.
Participant: yes
VN: how has this thing changed you? What things do you look at in your life and think that if this traumatic thing hadn't happened, your life wouldn't have turned to the way it is?
Participant: yes its…
VN: what things do you look at and say you wouldn't have been this person; you would've turned out to be someone else?
Participant: it is not completing school and my sister fighting with her man.
VN: so how have those things changed you? What have you become now that you wouldn't have been if… or something you wouldn't have done if that didn't happen?
Participant: I would've continued with school, until I reached tertiary or something like that.
VN: and do what when you reach tertiary?
Participant: then I'd continue to study for a good job, because I wished to be a lawyer.
VN: so, when you come across people who are fighting each other how do you feel?
Participant: I don't get close to them; I warn them and tell them that's not right. Because I am also not someone who like hitting. I'm not someone who's violent
VN: what do you do maybe when you witness a violent situation?
Participant: I move away from them or break it up.
VN: what do you do?
Participant: I break it off

VN: oh okay. Doesn't it remind you of what happened to your sister?
Participant: it does remind me sometimes. That's why I don't like it to happen in front of me again.
VN: so, what do you do?
Participant: I break it off if it happens and tell them to stop what they're doing.
Dr.V: so the things you do, when you wake up in the morning and the way you do things, do you think your sister's thing has affected you if you look at it?
Participant: hmm what can I say, it did affect me because the studies at school I wasn't able to cope with them. Because on the days when they were fighting, I ended up staying with the neighbours, sometimes I'd sleep at the neighbours and couldn't study properly
VN: aha
Participant: and at school I couldn't concentrate because I heard her voice because I'd wake up in the night and she's calling me painfully. Because from her voice you could tell she needed help.
VN: does that still happen now?
Participant: no that doesn't happen anymore, it's an old thing.
VN: oh okay.
Participant: yes
VN: and then uh but here you're. you're still alive and you didn't give up hope
Participant: yes
VN: what has enabled you to carry on with life?
Participant: the fact that hope doesn't kill. And that I still believe my things are going to become right. What I wish for is still going to be fulfilled, it just needs me to stand up for what I want to do.
VN: what do you plan to do, that you feel like you must stand up for?
Participant: I'm thinking of going back to school, and maybe do the night shift. Finish up school using night shift, so I could fulfil my dream of becoming a lawyer.
VN: that's good
Participant: yes
VN: and then… so, when other people get admitted in hospital they're brought by force against their will. They get forced
Participant: yeah
VN: did that happen to you?
Participant: no
VN: who brought you here?
Participant: my sister
VN: okay so you had an agreement with your sister?
Participant: no, I was coming from being admitted in [hospital].
VN: admitted for what?
Participant: I was smashing windows at home and doing all that is not right, here in [location]. So, I was taken by the neighbours, I think they called the police and police came and put me inside the van and took me to [hospital]. I stayed there for 2 weeks.
VN: okay. So, how was that experience for you of being taken by police?
Participant: uh

VN: they didn't tie you up at [hospital]?
Participant: no. they just locked me up in a room for one day because they said I was fighting. Yes, they said I was fighting so they locked me up in a room for 1 day.
VN: do you still remember the experience of breaking windows?
Participant: no, I don't even remember what…I still remember it, I do. But what had gotten into me I don't know.
VN: okay. So, in terms of your admission you didn't have bad experiences? You didn't have anything bad, traumatic?
Participant: when I got out, I didn't do anything that brought me to this hospital, because when I got discharged from [hospital]. Because I was discharged on [day], on [day] I came here to [location] and got there and found my cousin I haven't seen in a while. I greeted and he greeted back nicely then I sat down for some cool air outside and he made me speak to my aunt on the phone. He got my aunt on the phone and my aunt asked me how I am, and I asked 'to live here', and she said 'there's no problem if I won't trouble children', the I said 'I won't bother the children'. Then she said 'okay' and shut the phone, after that a girl I didn't know came, I had never seen her, she had grocery and she put it inside. She went out to buy bread and put it inside the house. Thereafter an ambulance came and grabbed me by my arm and put me inside
VN: hm. So, you were supposed to go back to [hospital], and you didn't?
Participant: yes
VN: but nothing bad happened in your illness and return to hospital and admission?
Participant: nothing happened to me. I was disturbed in my mind before coming here
VN: oh. What do you think disturbed your mind?
Participant: I think its being ill, being psycho acure, acure psych. Because this is not the first time, I had been admitted in [hospital], in [location].
VN: what do you think causes you to become ill?
Participant: I think it's the drugs I'm taking
VN: oh okay.
Participant: yes
VN: is there anything you'd like to share with me about another traumatic experience you've had that you haven't spoken of?
Participant: no there isn't any other sister.
VN: okay. So, in your opinion, the people who are in your situation, how can they avoid getting to the position you are in? if you could speak to the community and give advice, what should they do to avoid being in your situation of being sad?
Participant: they must surround themselves with people who make them happy, people who always love them. And speak and belch everything that is inside, because sometimes I was destroyed by secrets because I didn't have anyone to talk to.
VN: hmm
Participant: yes. And speak everything that is inside without fear and be able to find help in that way. Even if they don't receive help but can be able to burp once they've spoken to someone.
VN: okay. Who were you supposed to speak to, but you didn't?
Participant: I'd say friends because they were the people I had beside me at that time when all this was happening. At school
VN: but you didn't?

Participant: yes
VN: oh okay. So, you suggest that when people have issues, they must talk about them?
Participant: yes, and not enclose the chest.
VN: hmm and then seeing that you're already here, what do you want us to do as health care workers? If the president was to come and ask for your advice as to what to do, what would say, what advice would you give doctors to help people in your situation?
Participant: by listening to them when they speak and treating them well and not shouting at them. Because another thing that damages me is a person shouting for no reason you see?
VN: hmm
Participant: that makes me scared
VN: why does it make you afraid?
Participant: I think they want to fight me or something like that.
VN: okay
Participant: when someone shouts for no reason when I'm talking to them calmly. And they start shouting. It's something that happens, we're not the same, other have anger you see? Its something that happens.
VN: do you think it reminds you of…
Participant: yes
VN: who does it remind you of?
Participant: the memories where my sister is fighting with her husband.
VN: when someone is shouting at you?
Participant: when someone is shouting at me
VN: oh, so you wish when someone talks to you they must do so calmly?
Participant: yes
VN: and listen?
Participant: yes
VN: oh okay. Your advice is very good, I understand it. Okay is there else you'd like to share with me?
Participant: no not for now.
VN: okay thank you so much brother
Participant: yes

Transcription for QTS15

VN: I'm going to ask you to speak freely. As I've said our study is about helping people to cope with their traumatic or upsetting experiences. Can you tell me what has upset you in your life?
Participant: it is the issue of being bullied and when you go back home to report, it'll be as if they get you but they're not paying attention. Especially at home, when you say, 'someone is speaking like this' they say, 'oh please' and all that you see. I don't know how to describe it; I am laughing but hey. Instead of a parent coming with a torch, and maybe I'll come crying while trying to fight off an elder and I'm angry and they're picking on me and I tell them I don't like it, you get laughed at by everyone.
VN: hm
Participant: and in a painful way, not laughing normally, the loud laugh and they even come up to the face laughing. When you go home to report that person because they're older, instead of him being fetched from his home and told never to do it again, I'm the one who will get a beating. And asked why I can't fight for myself, I must go and never come back to this house to tell on someone. So that's how I was raised and those are the things that upset me. But then I would sleep, or just end up crying
VN: hm how old were you at that time when it began?
Participant: I hadn't reached 10 yet.
VN: what grade were you doing?
Participant: what can I say, what grade is standard 1? Grade 3
VN: standard 1 oh okay. Standard 1 is fine
Participant: yes grade 3. because I began realizing that I speak the way I do, the way I am from grade 2. If you notice these people take it as a joke. But if I also heard someone else, I did feel like laughing
VN: so, you're saying the thing that caused you to be bullied is the way you talk?
Participant: yes, it's the way
VN: how do you speak?
Participant: I have tongues, they call it stuttering.
VN: oh okay
Participant: I call it tongues because if you say its stuttering, it hurts.
VN: that's a bad term?
Participant: yeah so, the way I reacted was to cry. Maybe when I was in school and couldn't cry, I feel angry and catch myself having already hit that person. But at home I failed to do that because there were parents and they are ones saying I mustn't come to report. And when I hit everyone, they also the ones saying 'why are you hitting other kids' and then they hit me. Even in the house I am born with 3 sisters, we've grown, I'm the second from both parents, I'm born with girls. They were rude to me though
VN: rude about what?
Participant: about the way I was talking. When I reported I wouldn't be attended to and I'd get hurt. When I took my own decision that since this is how they're acting the way I'm going to use is by
VN: by hitting them?

Participant: by hitting them because I got angry to a point where I raise my hand. When I'm angry, talking…the voice doesn't come out, I fail to talk and raising the hand helps. So, I would get beaten, sometimes not with the belt but with a whip.
VN: as a young child you were hit with a whip?
Participant: when I was young. If wasn't a whip, then a leather rope called *Isitshwebhu*
VN: hm
Participant: sometimes I wouldn't do anything, and they would tell lies on me, because they know I would fail to defend myself. The time I try to respond they would say ahead lies on top of lies they were telling. When I try to talk, and I'd get tongue-tied and couldn't speak. And when I get angry, I'd be as if the one who is lying is telling the truth. And when I cried because they're lying, they'd say because 'you're crying the other one must be telling the truth, and you must be lying'. So, I'd get a beating for things I didn't do, which I wouldn't like to speak about.
VN: hm
Participant: so that's the way I can say I got bad treatment as a child. But now, when I'm angry I become angry I end at being quiet and walk away. And now I smoke this cigarette, when I'm angry I smoke it and feel calm.
VN: the cigarette or weed?
Participant: cigarette, tobacco
VN: just tobacco?
Participant: yes
VN: and then you've never used other drugs?
Participant: I've smoked weed and then drugs that remained habitual are cigarettes and alcohol
VN: oh, you've used those things. Did you use them when something was making you angry or just in general?
Participant: another thing I've used when I got angry is alcohol only, but even then, not all the time. When I drink it I just do not because I'm angry, but just to quench thirst.
VN: okay. when you look at yourself now as an adult, do you think the experience you had affected you?
Participant: I don't know, I wouldn't know because it began when I was still young. It still happens even now, when I am about to fall asleep, I would hear noisy things as if it's a lot of people screaming 'Yeeeaahh' and drum like things 'dudududu' before 15 years old. As if there were also cars, then I'd wake up. And then I don't know when I'd fall asleep then I'd wake up in the morning.
VN: aha. So, when did the hearing of voices begin when you're about to sleep? How old were you?
Participant: it began when I was very young. They're the ones scaring me and getting me admitted in hospital for the 3rd time now being admitted.
VN: so, you were bullied at the age of 10, and remembered you were 15 when you couldn't sleep without hearing the voices?
Participant: this thing of being bullied began while I was studying in the lowest grade but when I was in standard 5 or from grade 6, its standard 4 right?
VN: aha

Participant: I was beginning to fight for myself, and I was using this anger of thinking so that if anyone was disturbing me…because it wasn't unclear fighting with my sister, because it is my older sister, then me and the other 2 sisters. So, I learnt to fight for myself while I was in primary, it made me have a short temper. When I got to high school, I realized that having a short temper is not useful, because people use it against you. And knowing I have a short temper and they can do something, and I'll get angry, I can end up stabbing someone.
VN: oh, so it has made people to continue bullying you because you have a short temper now? But a short temper began because people were laughing at you when you spoke?
Participant: it begins by thinking its best to beat up somebody who's going to irritate me. And make it clear not to irritate me because I don't tease anyone. If we're making jokes, I don't like making jokes, you can do it on your own and not include me. Because I know you're going to make a joke about something I don't like. And I'm sure you're going to make a joke about something I don't like. I was saying that just as they were beginning, and I'd say wait before you make a joke make it about something that I can laugh about too, not something that's going to make me angry. That's how
VN: hmm okay. If I'm hearing you correctly, you're saying this has made short tempered, this thing of being upset? It made you change.
Participant: it began when I was small, it made me have a short temper. I don't know how I can describe it. But the thing of voices when sleeping began before knowing there are bullies at school. Or before I had a short temper. When I'm sleepy and trying to sleep it'll be like as if there's something that is screaming yeeaahh
VN: but they were not saying the things that these school children were saying?
Participant: no
VN: it wasn't laughing at you, were you hearing laughing voices?
Participant: no just screaming voices I didn't know where they came from. Noise, like really loud noise mixed up with other things
VN: had you begun using cigarette at that time?
Participant: no. I started using cigarette at high school doing grade [number] in [year]. Together with weed, I started it in high school about to finish. I started alcohol too in grade [number].
VN: okay is there something that made you begin? What causes that?
Participant: hey I don't know. With alcohol I can just say I got angry. I already had friends who smoked but I was able to hang with them from…people began in primary. I was able to hang out with them smoking, I'd pass a blow, as well as alcohol, I'd look at it while they're drinking in front of me. While I was still in high school, I just want to make you laugh a bit. When I was in high school, I began being naughty, another one involves a woman I loved. So, when I was angry, I said 'give me a cigarette and it felt nice. The next day again'
VN: you were angered by the girlfriend
Participant: the girlfriend yeah.
VN: she angered you?
Participant: yeah that's how I ended up getting into smoking cigarette and weed. From weed it was beer. What got left behind from those habits was cigarette and alcohol.
VN: okay. Have you ever thought or dreamt see this experience of being upset about this issue of being bullied? Or people who were mocking or laughing

Participant: no. but I can say that if we ended up not, what can I say, maybe I'd think that if maybe someone did something I'd be hurt. It used to come to mind that so-and-so said something or did something, it didn't pass quickly from me. It remained, it stuck
VN: was it because that person did it, or it was a small thing that became big to you?
Participant: it's a small thing. To me it…how can I explain this thing?
VN: when someone does something to you it doesn't pass. What do you mean?
Participant: it depends on what they did. Somethings pass, and others don't. like before, we're talking about before, right?
VN: hm
Participant: the other things I would let go, others I wouldn't. if you teased me using my flaws we argue. If we stopped at arguing and fighting it has passed, I don't care. If we didn't fight and I keep telling you I don't like it, if someone keeps doing that and we didn't fight, I would hit them. If they did it today, and tomorrow same story as long as we don't fight, or I find myself having beaten them
VN: okay what kind of things do people do to you? What kinds of things don't pass from you?
Participant: like teasing me, that is what was upsetting me.
VN: can you give me an example of teasing, like how?
Participant: like teasing me because of my flaw, when I'm quiet minding my business. E.g. 'hey, you stutter' you see all that
VN: oh okay. So, you couldn't pass from that?
Participant: yeah
VN: so, it wouldn't pass?
Participant: the time when they say that, I get angry same time and can't respond, and I'd continue walking away. they would carry on maybe and call me names and all that. When it has subsided and the people he was making jokes in front of are gone, I'd tell him that 'I don't like what you were doing, please don't do it again, behave'. If they apologize, alright we move from it. If they repeat it, I tell them 'you and I are not getting along anymore. Just know that'.
VN: and then you don't hit them?
Participant: no
VN: oh okay.
Participant: as I'm saying when I got to high school, I was no longer doing what I did in primary. I was thinking before I… as I am saying that I would tell the person that what they were doing, if the 2 of us were crossing paths I'd tell him 'listen I didn't like what you did. Please don't do it again, it'll make us fight one day'. If they carry on and I've got them like this, I don't know those who know me say my face changes, I don't know how my eyes become. If that doesn't happen it means I haven't taken out the anger. But if it does take place then it passes.
VN: okay so, as an adult are you someone who gets angry quickly?
Participant: I don't get angry easily, I won't say I get angry quickly right now. But there is this thing of temper and anger but then I just keep quiet and look. I choose to be quiet. If I don't respond that's when I don't get angry. Let's say I have an argument with someone the more they go…I choose to keep quiet
VN: the more they make you angry?
Participant: yes. Then I keep quiet and listen and listen, when they're quiet then I reply. because if I can respond to them at that time, I won't be able to speak I'll become tongue-tied.

The more they raise the voice is the more it feels like my voice box is tied. As well as, when I raise it and when I become angry.
VN: and when your voice is restricted, what do you do?
Participant: I don't know what happens to my head. It's like there are sounds, some veins pop and I can't even speak properly
VN: hm
Participant: when it begins its like… yes, all these things are happening, when I get angry Infront of people, its better if I get angry and it's just the 2 of us, I can get up and leave you. But when I get angry and there are people looking at us then I just hear these voices as if there's one saying 'look at this one he's making a fool out of you' 'this one is…' old things come back that I've been laughed at before, and one person made a joke about me.
VN: oh, so when you become angry when there are people you hear the voices that are not there?
Participant: yes, I could say that.
VN: do you think you would've had these things if you didn't have this experience of being bullied?
Participant: I wouldn't know, I don't even want to lie about that. Because the voices and this thing like cars still happens to me at night. Sometimes not all the time. I don't know what causes that, or sometimes I would hear heavy footsteps and shouting 'yeeaahh'
VN: hmm it doesn't happen when something has upset you?
Participant: no. It happened when I was still very young before, I came.
VN: so, at work, do you work or study?
Participant: I was working, informal job.
VN: when you were working and while you were studying it didn't affect you? That people have a problem with how you speak
Participant: at work I don't speak a lot. I don't talk at work; I just do everything what I'm supposed to do. Maybe when someone is speaking with me, I just reply to what they're saying, I speak less and then I keep quiet.
VN: why do you speak less?
Participant: someone else may only find out after 2 months or 3 weeks working with me, that I stutter. Or if they catch me in a wrongdoing because we slipup because we work in a [workplace]. I'd pull out a chip and eat because I'm hungry, the I'd look around and put it inside the mouth. And when my boss finds me, he catches me out when I respond, he might even say 'you're even stuttering because you're lying' and I'd say, 'it's just the way I speak'. And he'd say 'no you're lying, you're beginning to stutter'
VN: hmm okay. So, it hasn't affected the way you work?
Participant: no, even when they try, they fail because at work I become someone else. I just laugh; when someone is trying to irritate you, the one being irritated if you laugh. If you laugh and act foolish, they become angry.
VN: hmm that's how you handle it?
Participant: when I'm at work.
VN: and then when you're out, home?
Participant: when I'm out I just ignore. What would a stranger say to me? It's just greeting and greeting back and walking my way.

VN: and the people you know, the family members and friends?
Participant: there's not even a single family member at home who doesn't know I stutter.
VN: hmm
Participant: the people who know me also know I stutter. They don't say anything. But if I had to move and stay at for example [location] someone who may hear me speak could ask 'you stutter?' and I'd respond 'yes' then they'd know I stutter.
VN: hmm
Participant: I experienced the jokes a lot when I was in primary, and a bit when I was doing high school grades.
VN: and it ended?
Participant: yes, it came to an end.
VN: okay. You've spoken about being beaten up by police the first time you were admitted to hospital
Participant: admitted to this one.
VN: oh, to this one?
Participant: to this one
VN: what kind of experience was that?
Participant: I remember being in a police van, but I don't remember being naked. The only thing I remember is that I was naked. I don't know where I got the pants because the police don't say anything, I was told by the nurses. I woke up and I was dressed in clothes from here. Also, they didn't tell me, they told my mother.
VN: hm
Participant: and then my mother told me hey 'they say you were brought by police here, naked'. Because when I was in the police van it was as if it was driven by my own father.
VN: so, this whole experience was not traumatic, you were not traumatized by being brought to the hospital by police?
Participant: there's that thinking of who saw me. I stayed for about I think a month. I spent New Year's and Christmas here. At that time, I got upset that I had ever walked naked.
VN: hm okay.
Participant: that I went from [location] on foot, and came to town, unaware I was walking. Just walking and talking
VN: so, when you think about that, how does it make you feel, how do you feel?
Participant: what other way would I feel? I do feel sad. hey, I can't explain certain things.
VN: so, what saddens you is hearing what you got up to, while you couldn't sense it?
Participant: that is painful. Imagine hearing from someone else that you'd been…you just think a lot that maybe I pissed them off or whatever. They do speak, but they don't say 'you're mad'. 'you were seeing things, hearing things'
VN: do you remember hearing things?
Participant: I can remember because the last I came here, I remember. But what those people were saying I can't tell you. It was as if I'm out to be attacked, to get beaten. It felt like something was touching me, I don't know what causes it. This perplexes me. Because in the morning out of the blue I just become nervous and I don't know where it comes form, and I just find myself looking around. It's like I did something, like I did something and now I'm wanted.
VN: hmm and what kind of feeling is it. How does it leave you?

Participant: I just have fright and my body starts shaking, I shiver and sweat. I know I'm laughing because I don't have it now but if I had it now, we wouldn't be talking nicely so. I would be wanting to get out run away.
VN: do you see things are think about them or it's just a feeling you have?
Participant: I don't want to lie and say its things I think about or see, I don't know because when it happens it is happening at that time. And the more I try to resist the more it feels like the people are getting closer, I even run away. It's useful if mother is there and I tell her 'mom I don't know what's happening. It's like I'm hearing voices. Can you take me to the hospital?'
VN: aha
Participant: when I come to the hospital I come voluntarily because I become afraid, I also don't know what makes me afraid.
VN: so, do you use the medication you get from the hospital?
Participant: yes. When did I begin, this week? I use it. There's one pack left, it comes as a carton, and inside there are 3 packs. It's like sachets, there are 3 pills inside or 12.
VN: so, you use those?
Participant: I take them at night, they said I should drink them at night only.
VN: okay. So, you're saying you shiver as if you're afraid?
Participant: yes
VN: and you sweat?
Participant: yes. My eyes become red as if I was in a fire smoke
VN: are you able to breathe?
Participant: yes, I can breathe
VN: okay. Your breath is not affected?
Participant: no, no
VN: okay but you become afraid?
Participant: I just become nervous. My heart beats fast, it's like I see something on me and the voices. And I blink frequently. I remember other things, and others I don't know because if this begins before I go to bed then I won't sleep. When I try to sleep then it gets louder.
VN: okay, but you're surviving you're coping with life, you're fine and continuing with life. What helps you carry on with life after everything you have told me about?
Participant: I want to continue and live nicely you see.
VN: what gives you…that wakes you up in the morning to carry on and not give up?
Participant: I still want to have a lot of things. I still want to have my own house
VN: hm
Participant: yeah, I still want to have my own house, and businesses. Those are the things that wake me up, to be able to carry on
VN: okay. What assists you to be able to continue still?
Participant: I listen to music and if I don't speak to my baby and baby mama. It is better if I speak to my baby mama in the morning 'how are you, you're well? And is the baby okay?'
VN: are you still together with the baby mama?
Participant: pardon?
VN: are you still in love with the baby mama?
Participant: yes, we're in love
VN: oh okay. So, you wake and begin the day by speaking to her?

Participant: yes sometimes, and sometimes no
VN: hmm
Participant: but another thing that makes me fine is by listening to music
VN: okay. Does the medication have a role in making you well?
Participant: yes, I can say that because the last time I... I don't know what injection they used on me. Because the last time I was here was on a Saturday and I left at about 2 when they'd already injected me. I don't know how many there are. They put a drip, injection here, and at the butt, injection at the drip insertion. Thereafter I was told I'll receive pills. Then I keep taking those pills. But I missed out 2 times
VN: hmm
Participant: yeah, I can't sleep at night. This thing I was talking about comes when I become sleepy, then I remember I didn't take medication. When was it, the day before yesterday? Today is Monday, right?
VN: hm
Participant: it was on Thursday when I didn't take them, the thing came again before falling asleep fulltime. That thing happens maybe 3 times before I get restful sleep, then I wake up in the morning.
VN: okay. If you were to give advice to someone with a similar experience, what would you say to advise them?
Participant: if you hear it go to the hospital same time because you're going to do irrational things and be looked on by people, straight up.
VN: okay. In your opinion how can the people in your condition be assisted? What kind of help do they need?
Participant: I don't know. I don't want to lie because I don't even know if these pills are the ones assisting me or what. I don't know if this thing can begin right now, because it hasn't begun this year.
VN: on which year did it actually begin?
Participant: I could say in [year]. I was working at [location] in another [workplace]. It was in [month] going to [month], I think it was the whole of [month] until I was out in [month]. That year ended, I think it was [year], it caught me again towards [month]. They gave me medication and they got finished, I wasn't told to come and fetch other pills.
VN: hmm so, they just gave you and discharged you? Which hospital were you in?
Participant: I first went to [location], secondly, I came here that's when I was brought by police. The first time I woke up in bed and it looked like the ones that were holding me were trying to attack me, telling them to leave me alone.
VN: so how did you feel about the experience of getting tied? Of being tied to the bed
Participant: I felt like, you know when you wake up tied down, you realize that you've been trying to loosen yourself its even red now. Like this on the bed. I woke up tired I don't know how I can describe it. I fail to explain some of these things.
VN: I just want you to say how you felt, what you were thinking about this thing of being tied.
Participant: hey I don't know
VN: how do you feel internally?
Participant: I don't know how I can explain this to you. I woke up tied down, I even wanted to poop

VN: aha
Participant: I started becoming nervous, unclear there's someone next to me. When I woke up, I said 'sorry please let me go to the bathroom' I just wanted to get out and run away, but there was security. I woke up and just wanted a way to unclear I can tell you that, only to find that they said my mind got lost
VN: hmm so you were seen by security? What did they do, did they release you?
Participant: they let me go and showed me where the toilet was.
VN: so, between [year] and now, you've been hearing these voices? You kept hearing them, the voices?
Participant: yes
VN: was there ever a time where you didn't hear anything, where you were fine?
Participant: I can't say it is something I hear today and… I don't know why it happens because when I really hear them, you see by me showing up at the hospital
VN: aha
Participant: because it's something that makes me get up and move around the yard.
VN: okay thank you very much. Is there anything else you'd like to tell me, that you'd be upset by?
Participant: I don't remember any.
VN: oh okay. Thank you very much, your story will help a lot of people.

Transcription for QTS16

VN: so, as I've told you we're speaking to people and we want to know how they cope with the difficult situations they have come across. So, can you tell me about what you have told me before, that are difficult that you've come across. What happened to you?

Participant: since I'm sick now or?

VN: in your entire life.

Participant: actually, I went, my mother and father left when I was young school was hard. And I couldn't concentrate and reason I couldn't pass with A's is because I didn't have the school equipment. When I went to school, I wouldn't have shoes and I couldn't register, and I was placing a burden on my grandmother. Because I was getting disturbed in school, I couldn't study properly because when I wanted money to buy, when I was in school, I never used to buy. And that is what disturbed me, being sick at home all the time, and she wouldn't have money. I also didn't go for school trips.

VN: the what?

Participant: school trips.

VN: school trips?

Participant: yes, I didn't go for school trips.

VN: oh, you were not able to go for school trips?

Participant: yes. I was burdening my granny who I am with today

VN: so, you were brought up by the grandmother you've come with today?

Participant: yes

VN: okay. So, you said your mother and father did what, they left?

Participant: yes, they left. father passed away in [year] [month]

VN: your father did what?

Participant: passed away in [year]

VN: oh, your father passed away in [year]?

Participant: yes

VN: how old were you at that time?

Participant: I was 7

VN: and then your mother?

Participant: my mother died in [year]

VN: [year]?

Participant: aha

VN: oh, so you grew up without parents?

Participant: yes, I grew up without them, I lived with grandmother and uncle.

VN: so, you lived with your grandmother and uncle?

Participant: yes

VN: oh okay. So, they didn't have money to

Participant: my uncle didn't work. There are workers who had his job but when I passed, I went to work with him. To find some job so I'll have money to carry at school.

VN: how old were you when you worked with your uncle?

Participant: uh I was, I began when I was 17.

VN: aha

Participant: Yeah, I began when I was 17 until my uncle lived in his house

VN: who was not living at home?
Participant: my uncle. He has a house in Folweni
VN: oh, he has his own house?
Participant: yes
VN: and he lives there?
Participant: yes
VN: okay what was most traumatic to you? What was upsetting your heart?
Participant: that I was not like other kids. Even now I couldn't get my own clothes, they were cut out, granny's clothes.
VN: hmm who cut them out for you? Who handed them down to you?
Participant: my uncle cut them out for me, and my friends
VN: oh okay.
Participant: yeah, I would wear my friend's clothes too.
VN: so how did that make you feel?
Participant: like I was abnormal. Even my younger brother surpasses me, he is more of a man than I am. He had his mother looking after him, but me every move my grandmother assists, and I was burdening her too much.
VN: who were you burdening?
Participant: the grandmother
VN: oh, the grandmother?
Participant: yes
VN: what do you mean burden?
Participant: when I burden her. It's that I bit my tongue
VN: pardon
Participant: when I burden her
VN: aha
Participant: the problem is that I bit my tongue. And when I came here, to the hospital something happened. I saw things that other people could not, ghosts.
VN: what did you see ghosts?
Participant: ghosts yes. I saw ghosts. To a point where even water was changing
VN: the water did what?
Participant: it changed
VN: it did what?
Participant: it changed and looked like blood
VN: it used to change?
Participant: it changed yes.
VN: oh. So, when you opened the tap it looked like blood?
Participant: no when I was pouring them in the bucket, it looked like blood.
VN: like it was blood?
Participant: aha
VN: oh okay.
Participant: and it was like I was living with a snake
VN: aha
Participant: like I was living with a snake inside the house, that I didn't know.

VN: aha
Participant: then I began being ill. And my illness began by when I was home, I'd see a person riding a baboon. That's when I began being ill, I don't know whether he saw that I saw him. The top jaw on the left and one below they went to the right, and right ones to the left. At that moment the tongue felt like it wanted to come out.
VN: aha. Oh, before you were brought to the hospital.
Participant: before I was brought here
VN: oh, that was happening?
Participant: yes
VN: that the teeth were separating, other going to the left and to the right and tongue coming out?
Participant: yes, the tongue coming out, as if somebody is pulling it
VN: oh, after you saw this person?
Participant: yes, who was riding a baboon.
VN: it happened after that?
Participant: yes
VN: okay. So, what was upsetting from the things you have mentioned, that you've explained?
Participant: its that the life was at stand still, its still and other children are learning. Others are working and I can't tell someone when I must do something, I do it by myself. When I left school, I did [occupation], when was it last year?
VN: hm
Participant: aha I did security last year and I did so that if there are jobs because I can't go and study at unclear to pay for my transport.
VN: so, the grandmother wouldn't have been able to everything for you?
Participant: yes
VN: oh okay. So, what has kept your life at standstill? Why do you think you don't have anyone to pay for you?
Participant: yeah it is that I don't have anyone with power to help me.
VN: okay between the issue that your parents are gone and that you don't have things, you don't anyone who can pay for you when others do? You grew up with nothing?
Participant: yes
VN: uh which one upsets you the most when it comes to mind?
Participant: uh I think of my grandma that she might even leave this earth without me having done anything for her. That's what is always on my mind.
VN: oh
Participant: because she's old now. The years are going, and my life is at a standstill. I just had this thing that I don't know what I can do for her.
VN: you wish to do something for her? The grandmother
Participant: yes
VN: when you think about this, how do you feel in your body? Do you become sad, does the heart beat faster or what happens?
Participant: I'm not sure but it makes me weak to a point I can't even walk
VN: oh, you become weak until you can't walk
Participant: no, even to sleep I can't sleep. I just want to sit.

VN: so, you're unable to walk or sleep you just sit?
Participant: I just sit only
VN: oh, do you feel your spirit is down?
Participant: yes
VN: do you ever cry, or tears come off on their own? Does that ever happen?
Participant: it happens but I try to stop so she doesn't see me because if she does, she's going to become sicker with her arthritis.
VN: oh so no matter how you feel you shouldn't show the grandmother?
Participant: yeah
VN: you shouldn't show the grandma?
Participant: yeah, yeah
VN: how do you feel?
Participant: I tell her some things but others I don't tell her everything because I don't want to upset her.
VN: what do you tell her?
Participant: what I couldn't hide as I was sick, is that I was seeing ghosts, I was seeing people transform. Unclear
VN: a person changing how?
Participant: their face changing
VN: the person was changing their face?
Participant: aha
VN: okay the face was changing from what to what?
Participant: for example, you doctor, change to be my sister.
VN: oh, so when you see me you see your sister?
Participant: aha
VN: okay how many of you are home?
Participant: at home there is 6 of us
VN: aha
Participant: there is XX mom's older child, XX, me and then granny and then their 2 children.
VN: oh there's 6 of you in the house?
Participant: there's 6 of us
VN: oh okay. oh, so when you became ill, the person was changing faces to be one of the people you live with?
Participant: yes
VN: so, what else did you see?
Participant: that I used to see?
VN: aha
Participant: I was seeing food
VN: aha
Participant: even the food I was eating was changing sometimes. (*Laughs*) seriously
VN: to become what?
Participant: like I was eating animals. As if I was eating snails, and poop you see?
VN: hmm
Participant: yes, it was changing

VN: what do you think causes that?
Participant: by the illness, which I don't know.
VN: what caused the illness?
Participant: that caused illness? I had a dream as if someone was raping me because when that happened at night, I woke up and shouted for everyone. The underwear I was wearing I couldn't dress it up.
VN: has it ever happened that you got raped as a kid?
Participant: no, it has never happened.
VN: you've never seen anyone getting raped?
Participant: no
VN: you've never had an experience of being raped?
Participant: no that's not the case
VN: pardon?
Participant: that's not the case.
VN: oh okay. so, when you think about this thing you feel weak, and unable to move and you feel down, do you become depressed?
Participant: yeah
VN: you feel like that for how long?
Participant: less than 10 minutes
VN: less than 10 minutes?
Participant: aha
VN: and then what happens after that?
Participant: after that I just use the TV to ignore it, I just watch TV.
VN: are you able to breathe?
Participant: to breathe? I know how to breathe
VN: don't you have that pain in your chest or body?
Participant: no there isn't. I'd be lying
VN: okay. So, this thing that happened to you of not having parents and having only a grandmother, how do you think it has changed the way you are.
Participant: it's by going back to school to study.
VN: so, if this was not happening, how would you have turned out?
Participant: now?
VN: yes
Participant: maybe I would've found a job and be working. Because it's the priority thing because education because education requires money meanwhile at work I'm going to get employed and know at the end of the month I can do something else. And find a better job to live better.
VN: okay. So, the way you behave, how has it changed because of the experience you had?
Participant: my illness has taught me respect. It preserved me because I was roaming a lot. It kept me inside the house
VN: so, you were changed by becoming sick?
Participant: yes
VN: you became reserved after getting sick?
Participant: yes

VN: oh okay.
Participant: yeah, I was able to stay inside the house with my siblings
VN: so, before you got ill, how were you?
Participant: oh, I became a wild
VN: you became a wild?
Participant: I became a wild. I'd wake up every morning like someone who's working, go to friends and we'd watch the laptop, watch a movie and make fat cakes and eat and do everything there until I went home to go and sleep. So, I wasn't staying at home
VN: you weren't living at home anymore?
Participant: yes.
VN: what made you wild when you got with friends. What did you do with friends?
Participant: we'd smoke weed the entire day
VN: oh, you'd smoke weed the entire day?
Participant: aha
VN: oh okay. What made you do that in your opinion? What made you become like that?
Participant: I wanted to soothe my heart; I've never failed in my life; I was frustrated by failing.
VN: so, you're saying you did it to soothe your heart?
Participant: yes
VN: you want to soothe your heart from what?
Participant: using the drugs. I failed the previous year. I had never felt the experience of failing. Yeah, the people I was studying with are carrying on with their lives, they're studying well and they're getting paid from their education. I'm getting pushed back and I'm sick unclear.
VN: so, when did this begin, the wild behavior? When did it begin?
Participant: it began last year it was [month] [month]unclear
VN: so, you're saying that becoming sick made you better?
Participant: yes
VN: oh okay. What do you think made you ill?
Participant: the thing that made me sick was this cigarette
VN: okay. So not having things and not having parents, how has it changed you?
Participant: it changed me a lot because if my mother was still present maybe she would've long tried for me to study. Also, maybe someone could've helped my granny because no one helps her
VN: what about your illness?
Participant: I'm saying maybe if mother or father was still here someone could've helped granny.
VN: hmm. Okay.
Participant: yes
VN: but you're still here, you're fine what has enabled you to wake up in the morning, what has enabled you to cope?
Participant: (*whispers*) food
VN: pardon?
Participant: it is food
VN: food?
Participant: I think so I don't know.

VN: what helps you carry on with life?
Participant: it is seeing my siblings happy.
VN: sorry?
Participant: it is seeing siblings happy
VN: when you see your siblings happy?
Participant: yes
VN: how many siblings are there?
Participant: I have 2 nephews and 2 sisters. My brother stays in unclear and not with me
VN: so, who becomes happy?
Participant: both are happy because they have children.
VN: they're happy because they have children?
Participant: aha
VN: so, when you look at them you become motivated to continue with life?
Participant: I'd also love a child, but I don't have one
VN: I didn't get that?
Participant: I love children
VN: oh, so want to have a child too?
Participant: yes, that's the wish I have.
VN: sorry
Participant: that's the wish
VN: so, what are you going to do? Do you have a girlfriend?
Participant: yes, there is
VN: so, you want to have a baby?
Participant: yes, I need it. Like
VN: what is your child going to do about support? Isn't it you're also supported by the grandmother?
Participant: the granny
VN: so, who is going to support your baby?
Participant: that's the problem. it's just a wish, and not something I want to do immediately.
VN: oh okay. So, when you think that in future you might have children that's what keeps you going?
Participant: yes
VN: oh okay. So, is there anything else that you wish to tell me that has traumatized you?
Participant: I'd be lying doctor there isn't.
VN: pardon?
Participant: there isn't
VN: there isn't anything else?
Participant: yes
VN: okay. Thank you so much for your time.

Transcription for QTS17

VN: as I've said our research is about how people deal with difficult situations they come across in their lives. So, can you tell me what are the difficult or traumatic things you've come across in your life?
Participant: okay. The real situations, I don't want to lie I'm from unmarried parents. They're not together but I ended up living on my dad's side. The painful thing that came to me was that while I was staying at mother's I lived nicely. I was back at the farms with my grandmother, although mother was drinker, she wasn't there all the time, but my grandmother treated me well.
VN: mother used to drink?
Participant: yes. She drank alcohol, way back she used to go and be nowhere to be found, granny used to take care of me, so I used to enjoy life. So, while it was still like that my dad's side came, they sent my aunt, aunt XXX. She stole me when I had been herding, I was 5 years. She stole me and left without seeing my grandmother. I was also still young; I was excited to ride a car. When I got here, my grandmother and grandfather were present. It wasn't that bad when grandmother and grandfather were still present, maybe because they sent for me to be fetched.
When they died, these aunts began abusing me. I couldn't live nicely, as it is, I'm coming from home and they're fighting me but I'm a church goer, they don't like going to church. I'm a joker, and they tell people that I'm mad. That's what they advertise, and now the people are thinking I'm mad, but I've got God's gift. Since I was staying with grandmother, she opened my eyes and took me to church. When I got to [city] I was brought to incense. After being told to use incense I could tell this was not for me because I didn't grow up with this. The thing I was doing, gran's thing was working for me. Now they're forcing me with this, telling me that ancestors are right. I'm telling them I don't have a problem with ancestry but when there's going to be a prayer and slaughtering of cows and goats, I don't have a problem. But I don't know, the way I was born and raised doesn't allow me to accept what you're doing. And they get really angry because they use potions something I've never used while I was staying with grandmother. So, when I was growing up with them, I didn't notice a lot of things, but I did them because I lived with them now, I can't come this way.
You see, so I studied, and they wouldn't even buy me uniform and I was begging people. I think there are about 6 or 8 aunts, one of them passed away. And my dad was alive, but he got injured. But he also supports them, let me just uncover it they're bad people. Because all the time they're always shouting at me. I'd understand if they were shouting at me and I was still young. But now they shout at me even at this grown age, which shows they don't like me. They wish to chase me, but they don't know how, and they're scared I'll tell on them at the police. Something tells me I should go to the police, but I always return, I am always crying, daily. And when they isolate me even people on the sides can tell I'm not supported in that part, and then they do whatever. People abuse me I'm always getting beaten up and you'd find that I'm always ducking people but through all that I like people and I try to show them what kind of person I am. I've done something else to try and show that I'm in trouble. My heart is, I have got painful blood in my heart. I make art from the streets, and people think I make art because I'm trying to have money and they give me money. But I'm trying to show that there's something about me, that's not what you think, I'm different. Although it's like that, they abuse other things and they ruin it,

you find that the police call me on air and say I'm making dirt, but it looks nice and then I am someone who is demotivated.
But I haven't given up. It's a big matter that in the family that one of them works under the law. So, they're the one who make me, who hands over, who sends police to come, and they come and beat me up so that I can't do a thing. But you find that I think about coming back but out of strength because he's in charge of the court in [town]. They inform him all the time and lie on me, and he also believes because he's got an evil heart. I proved that because when I had visited him, he said he won't look at this old person, I could see it's not about food it's his heart and he's showing me that he doesn't want me. He probably thought this would trigger me, but I saw something else, that he doesn't want me. But I was fighting to get my own shelter, although I don't have a thing, but I'll try.
VN: okay. So, when grandmother and grandfather passed away, how old were you?
Participant: uh grandmother and grandfather from mom's side?
VN: father's
Participant: oh, from father's side. They died around the 90s
VN: how old were you at that time?
Participant: I was nearly 11
VN: so, they stole you from your mom's?
Participant: they stole me from my grandmother on mom's side, my mother's mother.
VN: did they treat you well? The grandparents
Participant: The grandparents, I didn't have a problem with them.
VN: so, the bad treatment began when they passed away when you were 11?
Participant: yeah, when granny passed on. That's when they began coming out. It was there but I guess I didn't take note of it.
VN: who are they; your father's sisters?
Participant: yes, my father's sisters, and he was teamed up with them
VN: and your father's brothers?
Participant: yes
VN: and what did your father do?
Participant: he was also teamed up with them. I sometimes think I wasn't born from them, I also don't know, because you're told when you're already born that this is your father.
VN: so, you have got a feeling that you're not born there from the way they treat you?
Participant: because of the way they treat me, because…
VN: oh, since you were 11 you were feeling like this?
Participant: ever since, I was feeling like this. Because it's a lot, I was the only one getting beaten because they had many children but only, I got a beating. If I'd gone to play only, I would get beaten. Only I would be left to bath with cold water.
VN: hmm
Participant: even the electricity wouldn't be there when I was present. If they were cooking once I get there, they stop cooking because I am present.
VN: so, they didn't eat?
Participant: they do eat. So, there are outside buildings. So that's how they tricked me that since they stay in the outside building I used to live in the outside building, then I was kicked out from there. And I was somebody who was poor, I left and realised it was bad and came back.

So, some were ill, those who abused me the most. They're got HIV now and they're treating it, it's treatable. So, while it was still like that, they went back to that old thing and they didn't even apologise for that. Because even back then, I tell them but they're stubborn and they know nobody is going to help me. So, there is no one I am going to ask from.
VN: so, when you think about this abuse at home, how do you feel when you think about this whole thing?
Participant: I become grateful to God because I should've been be someone who hangs himself. What would I be doing? I would go to the forest or wherever there isn't anyone, and cry. Cry until I become tired and forget what happened. At all times I go and work, as it is, I left my bag I can't work for nothing. I even ask for food from other people because I tried asking for stoves and they would take them away and I'd just tell by seeing them not there anymore, one of the aunts. So that's that I don't know how I can be assisted.
VN: okay in this situation, what makes you sadder when you think about it?
Participant: what makes me more upset is because I hadn't done anything. Maybe it would've been better if I was someone who… I don't drink or smoke because of my parents. I realised how they became, so I hate this. Maybe it would've been better if I was troublesome or smoked weed and come back whenever, the way they make me seem as if I'm someone who does that, but they split and then I protect myself. And it's like I'm also fighting, you see, and the way I'm shouting, I've also learnt speaking loud. You see, just talking, shouting and become stubborn because I'm trying to block evil spirits, they have evil spirits.
And as I go to church, they don't like it. This other one from my aunt who passed away, he recently insulted me and said you're drunk; he wrote write that letter. And I kept that letter and asked who wrote it and found it was him. He lives in another house, they also abused him, and he left home. But he realises me staying here and he adds on, he fights with them to help them. And I tell him father even if you see me doing something wrong, I'm not saying I never do anything wrong but for example let's say I left it like that without cleaning. Because I leave it in a mess because they steal my money after receiving it. Because they search for it, then I leave it a mess. Then it's as if I don't clean, but I'm trying to prevent the thieves. The aunt that fights with me often, its her and her child, they steal. And they have taught their children that whenever they do something, to challenge me, and not do things for themselves. And have this mindset of oppressing me, they've also learnt to show me things and compete against me whether someone is buying a car or whatever. So that also upsets me because I can see, as I've searched, I search, that's what I can say. I search first, yeah.
VN: so, when you think about this do you dream about it, or think of it during the day and have a picture of it when you're seated?
Participant: uh…
VN: is it something you think about all the time?
Participant: to be honest it happens, since he's written that "you're drunk". I'm somebody who's been created with deepness, so that hurts me in my mind.
VN: then what do you think when you get hurt in the brain?
Participant: I mean in the mind they hurt me, because I'm thinking now.
VN: so, you think about it all the time?
Participant: all the time, as you know whenever someone makes you angry, you think about them. It happens

VN: do you ever try to not think about it, and want your mind to not think about it?
Participant: no, I forget because I go and listen to people, I love people because I become friends with people because I can see they are the same as me. So, what I'm discovering is, the thing I'm doing when it happens the family fights because there is a lack of understanding. So, I don't know if you could give me a chance one day to talk about families, talk about people who steal because this doesn't help me anymore. I must help build a normal life because nobody will speak what I wish them to talk about.
VN: aha.
Participant: but for the whole world to hear, because I have experienced that some of it is because it is not taught in school or whatever. And make important something that hurts, something that is right they say its wrong. When you take time to think about it in life, wrong things, even the wrong police they say they're right, and the right thing they say it's wrong
VN: okay what do you want to tell the community?
Participant: I didn't get that ma?
VN: what do you want to tell the community? To the people, what do you want to say?
Participant: uh if I were to… I wouldn't talk about it like we are now but if I was given a chance to talk about it I would do it and let it be heard by everyone. And that thing doesn't need one day, for a whole month, but when you're listening, I'm telling you, you wouldn't move from listening it. You won't feel sleepy too, because it would be something else, not because it came from me but…
VN: okay the problem is that if people don't know what you're going to say they won't give you a chance to talk.
Participant: as I'm saying, please listen to this carefully. When I speak about this thing, I don't want to talk about it like I'm saying. I know that When I'm given a chance to talk about this, then I will speak of it there because its something important, you don't just hear it and it ends there, the thing I'll speak about at that time.
VN: okay so this thing of having parents who are careless and drunkards and the death of grandparents and fencing of the place you were staying in how has it changed your life? When you think about it
Participant: hmm I'm going to explain something to you that is overlooked, when someone on earth is created, they're created not knowing what's going to happen. You're just placed in your mother's and father's stomach, when you arrive on earth there's no menu to tell you what you came for, what's going to happen. And then you find other people who also came in a situation and everybody is operating, it depends on where you are. So, the other things they, they…what can I say they happen, the tough things because most of the time you find that you're negative, others are fighting for life.
VN: okay so how has it made you?
Participant: how do you mean?
VN: so, all the that happened, happened. So how has it changed your character?
Participant: so, as I've said boss, be careful, I'm deep. As I've said that when I was born in the world, I'm talking about me but I'm making you open your mind to I'm being with you. So, when you're born you don't know what what's going to happen. And as we move on from now, we don't know what's going to happen. so, I might say I'm going to do it this way but then you find that when you go from here it doesn't become like that and it becomes something else. But what

I'm saying is that, its obvious being upset. As I am saying I'm also asking for a chance for me to…what I wish is to…
VN: hm when you think about being upset, does your heart beats fast and you're unable to breathe?
Participant: there is a condition that attacks me, I don't know what it is
VN: what happens?
Participant: maybe when the flu times come, I run out of breath in school, it runs out. As if I have breathing problems and I even put
VN: is that how it happened in school?
Participant: no, it has never happened. It began when I was old, when I was even done with school. So, as I am saying it's possible that as they're fighting with me, they're bewitching me. But there are those doing evil things most of the time. But I didn't know because nobody shows
VN: have you ever been told you've got asthma?
Participant: no when I get there, I'm told I don't have it, I'm fine.
VN: so that used to happen. Does it still happen even now?
Participant: it gets me of guard and its dangerous. Because when I get there, when you get there you might find the parents have been for a long time waiting for an ambulance. and the pain goes away, but it's still there. But then the pain I've felt because of being unable to breathe ey I don't want to lie, it's like you're dead, it's like you're being beaten up dead the way it's so painful. So, they know me from [hospital], and they joke around, I'm also talkative and it looks fine but then I know its very hard.
VN: okay. So, in all the difficulty you've gone through what keeps you waking up every morning to carry on with life, to cope?
Participant: oh okay, what keeps waking me up in the morning to carry on…uh I told myself that maybe it's how I was created. Please don't forget the things I have said.
VN: I won't forget
Participant: The gift I have is powerful. It's a gift but when I go up to people to tell them, they get angry. And then the person has a problem, because I speak the truth. If you know of the government, I'm more than government's words, but perhaps I will not surpass those of Jesus. Because when I check with him, it's something closer but I must be given a chance. I'm asking from anyone that can.
VN: hmmm
Participant: be given a chance, to listen to what I want to say. Because there are a lot of things. And maybe those things too will unveil a lot of other things. Other small stuff.
VN: okay. Is there anything else you'd like to tell me? That is hard that happened to you, related to our talk today?
Participant: yes. It's just that there have been times when I... when I'd been beat up by police, beat up by security. And people, ekuhlaleni. You find that I'd look for help from the police station, about family matters but somewhere else in [place] when I visit the lawyer guy and they'd beat me up and spray me. Uh a lot of securities in Stanger beat me up, people… But then others loved me, you find that they loved me, and they find that I have a gift. And they add to the fire and making their own quote. You find that I'm being beaten up by someone who knows information. At first you don't do anything, because you don't know, and then when you

know about me you forget that you didn't know about me. What makes you come in your situation is because you know about me, I have also told you everything.
What I do also has an influence, it makes a person angry. I don't know what causes that. So, then I get beaten up, and get sold by my uncle wakes me up at 5 in morning for work. When I wake up and meet up with people along the way then they fight with me. Because when I look for that job in that way, someone on the side will speak. let's say now here's a nurse, I'm speaking to you the nurse here's a problem I'm hungry. And then someone on the side will interfere and say, "here comes this person, starting with you nurse". Then I calm myself and beg 'please don't be angry' because we don't live the same lifestyle, they eat and they're full. So, they're in fact blocking me. So, I have hardship, then they will become an enemy obviously, without me…
VN: so, you just always get beaten up randomly for no reason?
Participant: yes. That's the main problem. But the people provoke me and then it's like I'm going to go with them to perform, they like performing like Tira and become recognised. And you find that they come up to me jokingly and swearing me. Maybe they're passing with a taxi and says whatever 'it's just a mad person'. So being called a mad man something I don't like above everything.
VN: aha
Participant: and then there was somebody else that just fights you
VN: hmm
Participant: I'm hungry doctor
VN: oh, okay let's finish up the interview first. Thank you very much for your time.

Transcript for QTS18

VN: in your life. I’ll just place it here so that I can hear you.
Participant: okay. I’ve had the experience that when I was a child I got injured here, by the window and it left a scar. Then I got burnt even on the body, burnt by water. Because I was living with my parents, that I could say were negligent on me
VN: they were what?
Participant: negligent
VN: so, you were living with both your parents?
Participant: yes
VN: so, you think they were negligent?
Participant: yes, then I got burnt. Thereafter the parents passed away
VN: aha
Participant: when the parents passed away, we lived with granny. And when living with grandma we had to live with aunt, and aunty didn’t treat us well, she treated us badly.
VN: she treated you how?
Participant: with force, with a tough hand.
VN: oh okay.
Participant: we were abused, we were woken up in the morning around 5. And told to prepare water and make firewood and collect water and get to school late. And we couldn’t go to school sometimes without eating, and we would walk and not take transport like other kids and it was far where we went to learn, from home.
VN: so okay how old were you when you got injured with the window
Participant: I think I was 4 years old
VN: okay and then the burn
Participant: also, I got burnt at the age of 4
VN: what took place first?
Participant: first it was the burning. And then I got injured here from the window
VN: so, the burn where did you get burnt?
Participant: here on the chest and here (unclear)
VN: so, on the chest, arm, and stomach?
Participant: yes, and on the stomach
VN: oh, you got burn on the front side and the arm?
Participant: yes
VN: okay, you were still 4?
Participant: yes.
VN: so, you first got burnt and the burn healed and vanished?
Participant: yes. But it hadn’t healed yet because I had just been discharged from the hospital when I got injured here.
VN: oh! What burnt you, what happened?
Participant: I was burnt by water because I wanted to, I thought, where I went, I got in and there was a boy his name was [name] he was going to make food. Then I was sitting outside there was no one then I got inside when I was hungry and pulled the tea water then it spilt over me with the cloth.
VN: hmm okay. And then with the window what happened?

Participant: with the window we were playing
VN: so, where did you get cut, on your face?
Participant: yes
VN: how long is the cut perhaps, 8 centimeters?
Participant: I don't know how…
VN: okay. It starts from below the nose to the side of the face.
Participant: yes
VN: what happened then?
Participant: we were playing, and I was fetched when it was going to be my mother's funeral. I was taken from the hospital because there I stayed in hospital for a whole year because of getting burnt. So, I got home, and the other kids were playing, and I was following those older than me. I stood below the window, a window and was leaned against the wall which fell because of the wind. The steel from the window it fell and hit me here below the nose
VN: oh okay. So, when you got discharged from hospital it was to attend your mother's funeral?
Participant: yes
VN: and then you were hit by a window?
Participant: yes
VN: and your mother had passed on at that time?
Participant: yes, she had died.
VN: oh, okay and then what happened after you were hit by a window?
Participant: I was taken back to the hospital again.
VN: oh, so you were not available for your mom's funeral?
Participant: I was not present for mom's funeral
VN: oh, okay can you tell me from these things, how old were you when your mother passed?
Participant: I was 4
VN: and then your father?
Participant: my father died when it was just a year after, if I'm not mistaken. And then he died
VN: who?
Participant: my father. He got shot when we were with him inside the house.
VN: really?
Participant: yes. Shot by criminals. They had come for robbery they say, but when I found out that my father opened a case, for someone who…
VN: who did what?
Participant: who assaulted me.
VN: a person who did what?
Participant: who assaulted me.
VN: there is someone who assaulted you?
Participant: aha
VN: what is to assault?
Participant: they harassed me when I was young
VN: aha
Participant: they say he put a finger below.
VN: hmm

Participant: and then my father opened a case and then they killed my father so the case will be wiped off.
VN: hmm. Was your mother deceased at that time?
Participant: yes, she was deceased
VN: oh okay. Was it a male who did this, who assaulted you?
Participant: he assaulted me
VN: yes, was it a male or a female?
Participant: it was a male
VN: did you know him?
Participant: no but they knew him at home. [name]…they say [name] somewhere there
VN: so, he's the one who shot your father?
Participant: I think it was them who were trying to wipe off the case.
VN: and then what happened with the case?
Participant: then it ended because the person who was pushing me was no more.
VN: hmmm okay. And then what happened afterwards? Who did you live with?
Participant: we stayed with our grandmother. She could no longer live at the farms in [location]and we had to move from there. And we went to live in [location]
VN: wait, what caused you to no longer be able to live in [location]?
Participant: grandmother says that that place gave here bad experiences, so she couldn't live there. Then she moved
VN: so, she left because of bad experiences or she was afraid something else would happen?
Participant: I think she was fearful of that. That another person might get shot because it had already begun in the area to have gun battles.
VN: oh, there were gun battles?
Participant: yes
VN: at what time. In all you have told me, has anything ever happened in your childhood? Or in your life?
Participant: there is. My uncle also wanted to grab me when I was in university…when I was doing grade 8 there.
VN: hmm
Participant: I even quit school and went to live with my brother in [location] and separated from my family in [location]. And went to live with them because I was running away from that.
VN: so, your uncle wanted to do what?
Participant: to grab me, to also assault me
VN: oh, and did he assault you?
Participant: no, he didn't assault me. I left [location] quickly
VN: hmm so you went to live with who?
Participant: with my older brother. He'd found a job at that point
VN: yeah
Participant: in [location]
VN: is there anything else?
Participant: no there isn't anything else
VN: that you'd like to share. So, you've spoken about a lot of things and they're all painful. When you think about it, which one is more hurtful when you think about it, that causes pain?

Participant: the one I think of, that causes pain, is the one of when I was still a child I got assaulted.
VN: hmm
Participant: because now that creates a big scar that doesn't end. Knowing you'll always be a victim of abuse.
VN: so how do you think this has changed your life?
Participant: it changed it immensely because I didn't have anybody who was able to protect me when I was a child. And the one who was trying passed away early.
VN: hm. So, what hurt you the most is that the person who abused you did not get arrested?
Participant: yes
VN: oh okay. So, do you still remember the experience?
Participant: I don't remember it properly. I don't remember it
VN: but do you remember what was happening around that time in your life?
Participant: no, I don't remember because I was told by my aunts. They told me what happened to me.
VN: they told you at what age?
Participant: they told me when I was doing grade 6 at that time. I think it was a round 13 years to 14 years
VN: oh! Otherwise you grew up not knowing that?
Participant: yes. I grew up without knowing it
VN: oh. So, from about 13 years they told you, and then what happened in your life after that?
Participant: I changed and became a troublesome child. I just became out of order and was back chatting at home. And I couldn't cope in school and ended up failing grade 6. I failed grade 6
VN: you said you were a troublesome child. Besides back chatting what else did you do? What other things did you do?
Participant: they didn't want me to sleepover at my friends' and I slept over on purpose.
VN: how old was your friend?
Participant: they were also my age
VN: were there elders at your friend's place?
Participant: yes, there were, there was her mother and father.
VN: oh, what else did you do?
Participant: I ran away from home.
VN: and go where?
Participant: to my friend's place
VN: is there anything else you were doing?
Participant: no
VN: oh, you say when you heard of what happened to you, you changed?
Participant: yes
VN: okay. Do you still think about that now?
Participant: I don't think about it now because I can see that I need to try and become someone who is alright. But it comes up that my childhood was not a smooth one, a good one and that I went through difficulties.
VN: hmm

Participant: Because even now the brother who has been the bread winner at home, died. So, all of that combined and became an experience I can say became difficult because nobody else works. I can't even get a job.
VN: so, when did your brother pass away?
Participant: he passed away in [year]
VN: okay. So, when you were around 4 your mother died, 5 your father died and who did you live with?
Participant: we lived with grandmother
VN: and whose mother was she?
Participant: father's mother.
VN: okay, where?
Participant: we lived in [location]
VN: you lived with her until when?
Participant: until my brother found a job, the older one, [name]. In [location] and he gathered all of us, his mother's children, because there were 7 of us. [name], sis [name], [name], [name], [name], me [name] and lastly [name]. We lived with him in [location] when he found a job.
VN: who did grandmother remained and lived with?
Participant: then grandmother lived with aunty, and her child.
VN: okay and the uncle who attempted to assault you, where did live?
Participant: he lived in his house in [village].
VN: oh, he didn't live with you?
Participant: no, he didn't live with us because I've had the experience of not living at granny's only and that's it. We were also taken by aunts and uncles and separated us.
VN: hmm
Participant: because [name], me, [name] and [name] we were still young at that time. We'd get separated and one would live somewhere and have the experience of how things were done in that home. When you'd hardly gotten used to it, then you're removed and placed elsewhere to live. They were changing us around all the time like that.
VN: hmmm. And then when did the experience of your uncle take place?
Participant: I was at my uncle's in [village]
VN: oh, you were at his house?
Participant: yes, at his house
VN: and who did you tell?
Participant: I told my older sister, [name]. And she said if it happens again, I should tell her. And it happened again, he attempted because he was touching me on my body, and he was touching me in a way that is not right. Then I called her and told her
VN: where did your older sister live?
Participant: she lived in [township] in another uncle's house. At great uncle's, because the person who attempted was the young one. And then sis [name] told our older brother [name] to come and get me.
VN: they fetched only you?
Participant: they only fetched me because it was only me living at uncle's
VN: oh

Participant: yes, because [name] was living with aunty and [name] with grandmother, [name] with another uncle and his wife. Another one, an older one. As I was saying that we were just accommodated randomly.
VN: so, you've spoken that what is most traumatic is that you were almost abused at a young age
Participant: yes
VN: this matter is it something that comes as flashbacks when you think about it? How did it happen when it upsets you?
Participant: I didn't have flashbacks, but I had that I did become someone who was insulted and became a difficult child, taken as a difficult child. But then it's because of my experience; I was abused as a child.
VN: what is a difficult child?
Participant: a child that doesn't listen, is disrespectful
VN: oh
Participant: aha
VN: okay. have you ever felt like your spirit was down?
Participant: yes, it always happens, even now it still happens
VN: oh, but it began at that time when you were 13?
Participant: yes, when I was 13
VN: did you ever dream of this happening?
Participant: dream of it?
VN: having nightmares
Participant: I did dream of it.
VN: on one occasion?
Participant: yes, just once.
VN: it was not something you dreamt of in your dreams?
Participant: no.
VN: and then have you ever told yourself to forget this thing? To forget it
Participant: yes, it used to happen. But then I couldn't deal with my relationships properly which I entered
VN: aha. What was happening?
Participant: because I was someone who was angry, and unable to cope well with relationships
VN: aha. So, it has affected you in the behavior at home, and relationships, and then school?
Participant: no in school I failed 6th grade because I was not focusing properly at school. That's all, I failed only the 6th grade.
VN: oh, you couldn't focus because of this?
Participant: yes
VN: okay. So, when you think of it, I understand that it has changed your overall behavior, but when think in the moment what do you feel from the inside?
Participant: there was that why did it happen to me. You see, wishing that I was also normal like other kids. I didn't have that experience because another thing that was painful was when people entered the reed dance and as a child I couldn't go to the reed dance because I didn't know where my virginity had gone.
VN: oh! You were unable to join other virgins?

Participant: yes
VN: hmmm meanwhile you hadn't done anything?
Participant: yes, meanwhile I hadn't done anything
VN: and then what other thing had affected your life?
Participant: its just that.
VN: okay. When you look at your life now, how has it changed your life now the incident that happened to you? How has it affected your life?
Participant: I could say it affected it because of the things I have come across. Its one of the things that made me become a person who's not right. As I end up finding myself in a place like this, because of my experiences, and it became one of my experiences, my spirit was hurt.
VN: okay. You think the reason you are in hospital is because of your experiences?
Participant: yes.
VN: okay
Participant: because I found myself being involved in several relationships, because I couldn't be in a single relationship. Then I found myself with HIV. HIV is what caused me to be ill, even mentally ill. Because I wasn't taking my medication properly.
VN: hmm okay. When you first got ill until you were brought here were you seeing things or hearing voices?
Participant: yes, I was hearing voices, and didn't want to stay in hospital. I even got injured on my body, I got scratched all over my body running in a dark bush.
VN: you ran into a black bush from the hospital?
Participant: yes, I ran away from the hospital
VN: you ran away from the hospital, into the dark bush?
Participant: yes
VN: oh okay. What were you hearing? What made you run away from the hospital in the first place?
Participant: I was hearing voices telling me to run away. And not stay in hospital
VN: oh. So, you heard those voices, you ran because the voices were telling you what to do?
Participant: yes
VN: okay. Did you see things?
Participant: yes, there were.
VN: aha, what did you see?
Participant: I used to see shadows coming towards me, as if they're going to press against me, wanting to kill me.
VN: okay. How were you feeling about your life at that point when you saw shadows? Did you feel like your life was really crushing you?
Participant: yes, I felt like I was being pressed, as if I'm going to die.
VN: were you able to breathe or these shadows were also holding your breath?
Participant: yes, it was hard to breathe, it was hard to breathe.
VN: so, was it hard to breathe when you saw these shadows or it happened in spaces, out of the blue to have difficulty breathing?
Participant: it was hard to breathe when I saw these shadows.
VN: aha. What were you thinking when you saw the shadows?

Participant: I was thinking about, I was thinking about death. I was thinking they came to me to kill me. Or to finish with my life.
VN: kill you for what? Why did they want to kill you?
Participant: I also don't know
VN: okay. The voices that were speaking to you were only telling you to run away, is there anything else they were saying about you?
Participant: they were saying I should kill myself
VN: kill yourself for what? Why did you have to kill yourself?
Participant: kill myself because my life is useless
VN: how did you feel about your life?
Participant: I felt like, I felt like there was no going forward, like there was no success in what I was doing. But then my sister told me that I'm successful because I was able to graduate. And I should appreciate that because unclear I also did level 3, and I should be glad for that and look at it as achievements. That they're the good experiences I've had in my life.
VN: how many of you are there? You said there are 8 of you?
Participant: there are 7 of us
VN: 7. What position are you?
Participant: I'm the 5th
VN: so, there are 2 others younger than you?
Participant: yes, there are
VN: okay. So, your other siblings have never had bad experiences?
Participant: besides our parents' death, and our grandmother's passing, because grandma was a like a parent to us, no I don't know anything else.
VN: okay. Grandma passed when you were what age?
Participant: our grandma passed when we were really grown. When I was 22
VN: oh okay. Which aunt's place were living in where you were unhappy?
Participant: we were living with aunty and grandmother.
VN: oh, she was not treating you kindly?
Participant: yes it is her
VN: you lived with aunt and grandmother; couldn't granny see when aunt was mistreating you?
Participant: sometimes even when grandmother could see, she'd say "do not abuse unclear's children". Then it went on and grandma didn't speak for us anymore.
VN: hmm
Participant: she kept quiet and let it go, she just let it go.
VN: oh, and how did you feel about that thing, that grandmother didn't speak on your behalf anymore?
Participant: it was hurtful because there wasn't anyone to speak for us anymore. That was able to intervene for us on all this experience. Even when we got beaten up, we were beaten using the water pipe
VN: have you ever been beaten up until you went to hospital? Or got injured
Participant: yes, [name] had broken hands
VN: and was taken to hospital?
Participant: yes. [name] was beaten up by uncle [name] until his teeth came out.
VN: hmm

Participant: his teeth became loose and he had to take them out.
VN: they didn't get arrested?
Participant: no, they didn't get arrested because we didn't go to open a case. [name] was beaten up by [name] and her hands broke. As it is, she has the experience of not being able to use them properly.
VN: who is [name] ?
Participant: our cousin, older than us.
VN: whose child, is she?
Participant: our aunt's
VN: oh, the aunt she was living with?
Participant: another aunt. Not the one who was abusing us, another younger one. There were 2 aunts
VN: hmm
Participant: yes
VN: okay. So, is there anything else traumatic you'd like to tell me regarding other people or yourself?
Participant: no there is nothing else besides what I had, the experience I'd gone through.
VN: oh okay. So, you think it caused you to be sick?
Participant: being ill, I do have that in my heart there are a lot of things I think about. So, my soul is disturbed
VN: hm
Participant: and also, that I had a baby right after finishing school
VN: you had a baby at what age?
Participant: I was 22
VN: oh okay. How old is your baby now?
Participant: they're [number] years now. They'll turn [number] in [month]
VN: how was the experience of having a baby?
Participant: it was hard because I didn't live with my child. And she lived at her father's home, she left me at 5 months. And I couldn't live with her because I was staying at people's homes, without a stable home to live in with the child. Then they said at her home I should bring them, and I did. So, since their young age I've never had the experience of living with my child. She's stayed at her home since birth, until now I've never stayed with her.
VN: but are you able to see her?
Participant: yes, I can see her
VN: oh. How does that make you feel?
Participant: it makes me feel like I'm useless, to feel like now I can't cope with being a mother.
VN: but how is your child? Does she know you?
Participant: yes, she knows that I'm her mother.
VN: okay. So, the experience of having a baby, did you plan to have the baby?
Participant: no, I had not planned it. It just happened that I had a baby
VN: so how was the experience of having a child?
Participant: it was hard because I was not working. Even when I found a piece job, whenever I got paid from fort nights when I'd find a piece job, then I would go whenever I got paid from fort

nights and went to see and wasn't able to use money in a proper way. Or do a driver's license or carry on with school in that manner.
VN: hmm okay I understand you. But when I look at you, you're strong, you're still coping and carrying on with life. What gives you the strength to go on with life?
Participant: its because now I'm a mother, I've got a child. Even when sometimes it gets to me to commit suicide, but I don't kill myself because I know that my child needs me. I don't want her to grow up as orphan in the world, without a mother. They must grow up knowing their mother is present. Even if it ends with not finding a job but I am able to be there for them, even when they need advice or support, I'd give it to them.
VN: hmm. Can you see that your child is going to grow up better, nicely, with a caring mother?
Participant: it is better
VN: oh okay. So that's what came to mind and wakes you up in the morning, it's your child?
Participant: yes
VN: okay. What do you wish…if you could give advice for people who don't have your experience or people who do have your experience, how do you think they could be assisted? Someone who's had your experience, what kind of help do they need?
Participant: it is by being counselled psychologically.
VN: how?
Participant: properly by receiving counselling.
VN: aha
Participant: even though a person is not coping or if they had these experiences, to go on with life, and not let it be things that weigh on their soul.
VN: okay. At what point did you need counselling? At what point did you think you needed counselling?
Participant: when I was still young, when I was 4.
VN: hmm. So, when you think about it, you think people should be assisted with counselling?
Participant: yes
VN: okay. What else helps you, that makes you feel better?
Participant: it's the pills I'm taking.
VN: which pills?
Participant: the ones for the mind
VN: oh and how do they help you? What happens when you use them?
Participant: they heal me
VN: what is to heal?
Participant: it is to heal.
VN: oh okay. They make you feel…
Participant: there are these one I take; they help me feel good emotionally. They help me emotionally. Even though they have side effects I am assisted.
VN: oh okay. Is there anything else you'd like to tell me?
Participant: (*quiet*)
VN: Is there anything else you'd like to tell me?
Participant: I think it was just that only
VN: oh okay.

Transcript for QTS19

VN: understanding the traumatic experiences people have gone through. The things that make people sad because of pain
Participant: yes
VN: can you tell me what has happened in your life until now?
Participant: from childhood?
VN: yes.
Participant: from childhood my life was not right. My mother was working
VN: who?
Participant: my mother
VN: yes
Participant: but when someone wanted me to visit, my mother would just agree for me to visit. To a point where even the first I started doing it without staying with my mother. I went to [village] by grandmother unclear, I had visited there. I did grade 1 and 2 and I didn't go there anymore because there were wars. But when mom came to fetch me, they said I'm studying, I'm crying for those going to school, then I went to school with them. And then again there was her friend
VN: so, your mother in your childhood she was working and left you with who?
Participant: another grandmother called [name].
VN: she left you with another granny?
Participant: yes, but she lived at home.
VN: and she didn't want you to visit?
Participant: she let us visit, where you asked to visit, she'd take you. To a point where I lived in [village], as well as [place], and she would visit me there.
VN: oh, so your mother made you visit because she was working?
Participant: yes
VN: oh, so she'd take you to another house and another one and another one?
Participant: yes, but her relatives, her cousins
VN: oh, she kept taking you?
Participant: yes, especially me because my younger sister didn't visit. I was visiting frequently
VN: so, visiting, was it to keep you there in that place and not live with her?
Participant: no just to make me visit, and I would tell her I enjoy staying there.
VN: in that place?
Participant: yes
VN: hmm.
Participant: then she'd let me stay there, just like [place]. But in [place] my uncle [name] refused and said I'm not going to live that far and asked his friend. And XXX also said I won't live that far, and then I didn't live there. Then again, there was a XXX household where I did my washing during the weekends.
VN: how old were you at that time?
Participant: I was probably round the age of 11
VN: aha.

Participant: they asked me to go and live there. Then uncle denied for me to live in people's homes.
VN: and you wanted to live there?
Participant: I wanted to live there because maybe my life would've changed, maybe I would've finished school if I lived there.
VN: okay why didn't you finish school?
Participant: everyone who lives at [surname] homestead do not finish school. As well as daughters, I don't know why.
VN: so, everyone who lives where? Your mother didn't finish school?
Participant: yes, my mother's home. All of them didn't finish school, I don't know why. And all those who finished, did it because they didn't live there. They'd be taken to live in other places, then they finished school. But when you live there you don't complete school, besides the wives' children who complete it. But then now it's as if my child will also not finish school, it's as if they will be interrupted and not complete school like us.
VN: so, your child
Participant: my child, yes
VN: what happened to your child?
Participant: it's as if they will be interrupted, I'll just say that.
VN: oh. How old is your child?
Participant: they're 13
VN: so, you have anxiety that they won't finish school?
Participant: there's just a voice that says she won't complete school
VN: who said?
Participant: there is a voice that just keeps saying inside my ears, that this one is going to get pregnant. And I keep praying for her over and over. And her father and grandmother become angry when I pray for her and told not to pray for her because the thing keeps telling me to pray for her. And hold her here, in the stomach because she's had a problem; there's my sister's child that came for a visit and stayed there at my house, [name], he's tried to sleep with her. I caught him, and he tried again, and he went to [village] at his grandmother's. when he went to [village] at his grandmother's, his uncle showed him a gun like this and told him he doesn't want R200. If he could him R200 then he could do unclear. But they say [name] didn't do anything but now that's why I don't like it for a girl child to visit. Its better if they stay with you mother and father.
VN: oh, so the man who showed him the gun, what did he do to your child?
Participant: he didn't do anything, so he says. He says he didn't do anything because we came to the hospital. He said he asked for the key from his grandmother to go to the outside house.
VN: hmm but he didn't do anything?
Participant: no because we came to [hospital] with her dad and left her to get checked, and were told he didn't do anything, she doesn't have anything. And a case won't be opened because nothing was ruined.
VN: hmm. So, you have concern for your child, you have worry about her? How many children do you have?
Participant: there are 3
VN: and what spot is she?

Participant: [name]?
VN: hm
Participant: [name] is the second. There's one that passed away
VN: the first one?
Participant: yes
VN: okay. And then others?
Participant: the others are all alive
VN: two of them, so you've got two children?
Participant: I have 3 children
VN: oh okay. There are 3 of them
Participant: yes
VN: so, this one is 13?
Participant: yes, and the other one 10.
VN: aha
Participant: and then the one who is 3.
VN: oh okay. so, you've spoken about the worry for your child, right? Uh I'm saying the things that traumatized you in this world is that your mother made you visit, is there something that happened to you in these visits?
Participant: something did happen to me.
VN: what happened sister?
Participant: you see the time I was living with her sister?
VN: whose sister?
Participant: mom's sister
VN: oh, your mom's sister?
Participant: yes. Her man used to sleep with me forcefully. (*cries*) Now when I look at him in this grown age I feel like vomiting. I won't tell my aunt, and I won't tell my sisters too, because it'll be as if I came to ruin. My aunt once swore at me and said I 'make things happen', at the beginning, when I tried to speak up. I don't like it.
VN: here's some tissue.
Participant: me and my aunt were fighting for a place when my father said this place, we were looking for a place when my mother had passed on, and father said the place is where [name] lives, our aunt. When we got to aunt [name], aunt [name] called her man on us and her man was living elsewhere with another wife. Her man spoke the truth and said the place was bought by my mother with R300. She'd not finished paying her money, there's also [name], [name] and [name], aunt's children. I told them that is our home while we're homeless, father said it's the home she bought for us. There was silence and that passed but there is a letter that [name] wrote, the one who works there. Everything that we spoke she wrote down; I don't remember where she last kept it.
VN: okay. So, it was your aunt's man that was sleeping with you forcefully?
Participant: yes. He slept with me and said I must keep quiet because I used to work and live there and did cleaning and everything. He was the one who bought me clothes and everything, he bought me clothes and my aunt would be furious and say I receive everything from her man. She even told my mother I was dating her man.
VN: how old were you at that time?

Participant: I was very young I was about 16.
VN: were you blood relatives with this aunt?
Participant: my aunt was my mother's sister.
VN: she's your mother's sister?
Participant: yes
VN: oh okay. So, you lived at your mom's sister's house?
Participant: yes
VN: and then her man abused you?
Participant: aha
VN: you lived there for how many years?
Participant: I lived there; I grew up there. My mother preferred I went there than other places, because even when I was doing standard 3, 4, 5 I was living there. I only came back when I was a bit grown to do 6 and 7. Then I came back to live with my mother and not with them.
VN: when did you begin living with them?
Participant: I began when I was doing standard 2, 3.
VN: you began living with them when you were young?
Participant: yes, from 2,3,4,5 I was living with them. Then when I was about to go to high school I returned to my mother.
VN: when did he begin doing what he was doing to you?
Participant: ever since I went. And he was not doing it to me only. Everyone that came, my aunt was working, everyone that came the uncle would sleep with them. Even [name] another girl, and another sister that lived in [village] sister [name] too. And my aunt would say they want her man, and I'd also remain quiet and not say anything. Even [name] from home, she said Winnie is also doing what they're doing, that people went and got involved with uncle.
VN: hmm, so, this uncle raped everyone?
Participant: I guess he raped in style because you would keep quiet. I have never told anyone; I have never even told that to my child. It was the first time I told anyone
VN: you've never told your mother?
Participant: I never told my mother because she said they say I'm involved with him.
VN: oh. What happened to you is really painful because you were just a child at that time.
Participant: I was still a child. Even now I don't want my child to go visiting, even at my sister's or wherever, I don't like it. I want her to be on my sight, where I'm always going to see her.
VN: so, you say you lived there for 4 years?
Participant: yes
VN: at your…
Participant: my aunt's, yes.
VN: for about 4 years?
Participant: maybe even 5, I really lived there.
VN: oh, the whole time it was happening. What happened that led you to leave there?
Participant: I left when I got older, I realized I had grown up, then I left. I lived at grandmother's, and mom lived outside her home, in a shack. I lived and lived with her, while I was living with her, I got chased out by uncle. He said I was rude.
VN: your uncle chased you while living with your mother?
Participant: aha

VN: how old were you perhaps around that time?
Participant: when I was chased out by my uncle, I was probably 13. He kicked me out and told me to go away, then I left.
VN: wait, you went to your aunt's at what age, 8?
Participant: I must've been 8. I was still young, because my aunt is the one who taught me small house duties. Everything I was taught by her.
VN: it happened there.
Participant: even [name] was raised by me, and for [name] I was washing nappies and did everything.
VN: [name] and [name], who are they?
Participant: my aunt's children
VN: so, you went there when you were 8, but you were doing everything?
Participant: aha
VN: and the father there was abusing you?
Participant: yes
VN: then you left when you were 13?
Participant: yes
VN: to return to your mother?
Participant: to live with mother, yes.
VN: and when you got to your mother?
Participant: I stayed and stayed
VN: how old were you perhaps?
Participant: my uncle chased me out when a year was probably not finished, living with mother. And then my uncle said I should leave. Oh! I argued with his wife, I was always watching tv, *Days*.
VN: you were always watching tv?
Participant: aha
VN: what happened?
Participant: when I was watching tv his wife came and switched it off and said children do not watch this. Meanwhile her children did and told me to move from the couch. We didn't sit on the sofas we sat on the floor and her children sat on the sofa. We were always told to sit on the floor when watching the tv. I kept quiet, my uncle kicked me out and said I should leave, and I lived in [village].
VN: and who did you live with in [village].
Participant: in [village] I lived with some boy I was dating. He rented a room for me; he didn't live with me he lived with his brothers. I also didn't stay with him for long I was fetched by my sister Hle. She said I'm called back home, to come back. Then I went back.
VN: hmm.
Participant: but then when I went back that's when I got pregnant.
VN: so, you got pregnant with your first baby when you got back from there?
Participant: yes
VN: how old were you?
Participant: I was 14.
VN: oh, and you had your first baby at 14?

Participant: aha
VN: who was the child's father?
Participant: the child's father was some guy, who was working with my sister. It was not this one
VN: pardon?
Participant: it was someone else's
VN: it was not the boyfriend's? you were seeing each other, with the baby's father?
Participant: I was in love with the baby's father
VN: oh. How old is he, his age?
Participant: he was older than me.
VN: do you still remember his age?
Participant: I don't know how old he was.
VN: more or less, was he over 20?
Participant: he was over 20. He was older than me. And he didn't live here he was someone from the rural.
VN: was he over 30?
Participant: he was probably… the way he was built he was 20 something or 30 something, I don't know.
VN: oh okay.
Participant: he was the one who ended up buying me school things, uniform for school, he bought me everything when mom lost her job and was unemployed.
VN: so, when you came back you stayed for a little while, then was rented for by your boyfriend?
Participant: yes, in [village].
VN: and then your sister brought you back home?
Participant: yes
VN: when you returned home you met up with this guy?
Participant: yes. This guy was working with my sister unclear
VN: he worked with your sister?
Participant: yes, unclear
VN: okay. And then what did your sister say?

Someone: greetings
***Participant:** hello*

VN: and what did your sister say?
Participant: my sister didn't say anything because even Hle's baby dad was a foreigner, she found him while working with him. He drove their workplace vehicle
VN: oh okay. And how was the pregnancy because you were young?
Participant: I was young. It's something that ended, I came here to [hospital] to take out a tooth. And they said I shouldn't have taken out the tooth after that. Thereafter, I came back it was painful at night after tooth extraction the tummy was painful. It was painful in the stomach, there was pain and pain. And at 12 we opened a sewerage by our home, because they hadn't

opened the house at night. I couldn't poop at the sewerage, a water like substance came down my leg
VN: hm
Participant: and mother said I should go to the bucket and I went there. When we got there to the bucket that's when it came out and showed that the baby was small. And mom said we should call people and [name] refused to wake them up from the house. We called great aunt and Grandmother [name] and 2 other neighbors from home. They were called and showed unclear the baby. Then I came to [hospital] the next day with my great aunt.
VN: okay. How did that experience make you feel?
Participant: I didn't feel great, and I will never forget it, I always remember it because I went to church and confessed. I even named the child
VN: what? The baby okay
Participant: they say you don't name the baby.
VN: did you have a funeral?
Participant: pardon
VN: you buried it?
Participant: no, we didn't bury it because it was still something small
VN: it was small okay. And then what happened after that?
Participant: after that nothing happened. I carried on with life
VN: and studied?
Participant: I studied, yeah
VN: how was it at school?
Participant: it was fine
VN: you were able to concentrate?
Participant: yes
VN: you didn't have a problem?
Participant: no, I didn't have a problem
VN: okay until when?
Participant: until I quit school because mother no longer had the money for school transport, was out of a job and was selling at the station. And was paying for both of us, it was and [name]. [name] was schooling at [name] and I at [name]. And then I said mother I'm leaving school and went to look for a job and starting working.
VN: where did you work?
Participant: I worked at [location] in [location]
VN: oh okay. And then what happened [sibling]?
Participant: I worked, and the job there ended, moved to another place in…moved to [location] and worked somewhere else. In [location], I was taken in by [name]'s mother, at the electricity. When mom passed away in [year] that's when the job ended
VN: hm oh. Your mother died in [year]?
Participant: yes
VN: and your father, was he present in your life?
Participant: he's not in my life, even now he's not, but he is alive.
VN: is your father still alive?
Participant: yes, he's alive

VN: oh okay. In all that happened which one do you think is more traumatic to you? Which one is more heartbreaking?
Participant: all of it
VN: hmm
Participant: all of it. I think that even unclear my brother then maybe everything would be fine. Maybe I could've been working, or finished school.
VN: which brother?
Participant: my brother who passed away.
VN: your brother got arrested?
Participant: passed away
VN: oh. He died on which year?
Participant: I don't even know anymore, [name]
VN: how old were you when he died?
Participant: he died when I was in grade 2, he died when he was doing grade 11
VN: oh.
Participant: I wouldn't be suffering (*cries*) unclear. Because there is [name], he only put his sister at work. But we're present, we were always asking
VN: who is [name]?
Participant: [name]
VN: who is [name] to you?
Participant: my brother. he works with [occupation] unclear in [hospital] and he says it's not easy to put in people. But he hooked his sister up.
VN: hmm
Participant: even at [workplace] others are managers but they don't hook us up as relatives, they put in other people from elsewhere.
VN: hmm. Do you ever try to not think about this whole thing? All these problems?
Participant: yes, it happens
VN: what do you do to try and not think about it?
Participant: I'm fine, I become fine. But what made me think a lot is when I first began smoking weed
VN: did you think frequently and then started smoking weed or you started thinking after you started smoking weed
Participant: I started smoking weed jokingly, taking it and smoking…(*sobs*)
VN: when did you smoke weed? When did you begin smoking it?
Participant: I began unclear (*sobbing*) it brings back everything
VN: When did you begin smoking weed? Which year?
Participant: last year
VN: here's a tissue
Participant: it brings back everything, and thoughts that I'd forgotten. (*Blows nose*) another thing that upsets me is that when I check I am told I have HIV, when the father of my children checks he's told he doesn't have anything. My children's father is found with nothing, I don't know why.
VN: hmm.

Participant: he's checked several times which has caused me to discontinue medication because he says he doesn't have anything.
VN: oh, so that upsets you?
Participant: it upsets me
VN: why, what exactly is upsetting?
Participant: why doesn't he have it when we have babies together?
VN: oh, you wanted him to have it too?
Participant: he should have it too, where did I get it from then? Does it happen that you sleep with someone and even have a child, yet he doesn't get the disease?
VN: uhm did you see his results?
Participant: yes, I saw them
VN: okay. The problem is that people are not the same, in the way they're made up
Participant: hm
VN: there are women and their partner has it, but they don't. so, it happens
Participant: can I drink water?
VN: yes, sister you can drink. But then it does happen like that, some people are able to bypass it. But do you use the condom now to protect him from infection?
Participant: we don't use it, not because unclear he doesn't want it. He doesn't want it, at first it was as if he was putting in on, but he wasn't.
VN: who?
Participant: him
VN: the condom?
Participant: yes
VN: oh okay. So, what is he saying about him being negative and you…?
Participant: he doesn't say anything
VN: oh.
Participant: he doesn't have a problem with that. What confuses me is why he doesn't have a problem with me having the virus and him not having it. He even said he's not sick he doesn't have anything.
VN: so how does that make you feel when he speaks like that?
Participant: I become sad
VN: you also want him to have it?
Participant: yes, where did I get it from if he doesn't have it, meanwhile we both slept together? That means…oh they said at the clinic that doesn't happen, the person has it, but it hides.
VN: uhm I don't know because we didn't test him. But it happens that one person it and the other doesn't, that's why you must be careful. And perhaps you'd gotten it from the previous partner. There were others before, right?
Participant: yes. He also had them previously, his other girlfriend even passed away. That was dating another man who drove taxis.
VN: no, it does happen. We don't have answers for everything, but it does happen. The important thing is being careful and behaving well now. And also protecting yourself and using medication, uh and using condom so that if he gets infected to not infect you. But it happens. So, what has brought you to the hospital now, what happened?
Participant: supposedly I have lost my mind, they say.

VN: so, when did you begin smoking weed?
Participant: last year.
VN: okay, what was…why did you even begin using it?
Participant: it was just stress from worrying. Whenever I was worried. He was working, sometimes money was not available in the house I wouldn't know what's happening. And his mother kept on talking, saying I'm spending her son's money, just talking.
VN: so, your man works?
Participant: yes, he works for his children
VN: and his mother?
Participant: she just kept talking. It becomes a huge cause because she loves talking
VN: what does his mother do?
Participant: she also works at a joint and says she no longer gets her son's money I'm using it.
VN: she doesn't say it is spent by grandchildren?
Participant: she says it is spent by me
VN: hmm. So, then you smoked, and when you did every thought came?
Participant: yes
VN: and then you heard things, you heard voices?
Participant: but the voices I'm hearing, I see some of the things happening.
VN: like what perhaps?
Participant: like a lot happens. When I first realized this was working, is when I saw Nathi. I was very pained for [name] I prayed all the time I just prayed. Another sister [name] even said when I pray, I should pray with my young child and hold them by my hand. God hears the prayer quickly when you pray with the child, the child is innocent. When I looked at [name] and his mother was arrested
VN: how old is [name]?
Participant: [name] is the same age as [name], [name] is born in [month], but [name] is born in [month, year]
VN: [year]
Participant: same year [year]
VN: oh okay.
Participant: when I look at him
VN: your child?
Participant: no, the girl's child who used to be my friend [name]
VN: okay
Participant: [name] had been arrested, [name] was arrested on the [date] so I heard.
VN: so, your friend is arrested?
Participant: yes, on the [date]
VN: she has a child?
Participant: yes
VN: [date] of?
Participant: [date]. They say she killed someone there, [name], another one is not pointed out yet. She's running from the police. [name] ran away and came on the 1st 'you'll see'. She was seen by children they said she was beaten, injured and the police only found her on some other day. When the police found her, they took and arrested her and Lange came back and told me

that they found this woman, they found her at J. and then I said, ‘oh let’s hope she gets out soon’. The baby lives with [name], [name] is form the uncle, [name]’s father.
VN: hmm.
Participant: [name] loves children, I’m not saying she doesn’t but there’s that treatment showing that they’re not hers. And then I told [name], I looked at Nathi the other day I can’t remember what day it was.
VN: [name] is a child from there?
Participant: [name] is my friend’s child. I said [name] your mother is going to come back, [name] said when? I said but she will definitely return. Not much time passed by, I also tell you your mother will come back, right after I talked to him, they said there’s his mother calling him from across. It happened again
VN: calling him from where?
Participant: from the street. She was out of jail.
VN: so, her mother was discharged?
Participant: yes, they said she was out on bail. When I’d gone to [location] during the day and told her [name] will be out as you are abusing her children. But I didn’t say to [name] who is abusing the children. It’s one of the things that happen.
VN: some of the things that don’t happen you began seeing them after smoking weed?
Participant: I see things that become true. When I smoke weed that’s when I see things clearly after smoking weed.
VN: after smoking it?
Participant: yes
VN: it’s clear after you’ve smoked?
Participant: yeah
VN: oh okay. So, smoking helps you?
Participant: I see it as assisting me
VN: you don’t view it as causing you to get admitted in hospital?
Participant: no. you know since I’m not receiving it, I get headaches, I don’t sleep. But when I use it, I sleep deeply.
VN: so, the things you were telling me about, do you ever have flashbacks of them? Or think of them, or when you think of them, you’re unable to breathe and the heart beats faster? What happens?
Participant: when I think my breath is cut off and I cry. While alone in the house, and I cry.
VN: but the heart doesn’t beat faster?
Participant: I don’t know but it’s as if It’s going to get shut off
VN: it’s as if you’re going to get shut off? Oh okay. So, in all these that happened to you, did it change the way you do things or your being, the kind of person you are?
Participant: the life changed, I left in [year] to get baptized in church.
VN: to get baptized where?
Participant: at church, [church]. But then I stopped church
VN: uh
Participant: I’d go to church in [location], I’d also go everywhere and the church of the Saved, and they are sometimes wary. But when I was young living in [place] I used to go to the Roman church with grandmother.

VN: so, what did you do? Why did you leave church?
Participant: [church]?
VN: hm
Participant: something just told me to leave it. The woman from the top gave me a hiah but then that was the thing she used to make me stop going to church. There's a woman who was my neighbor, she gave me a grey hiah, [name]'s mother. I was not thinking about church at all. I didn't even think about going to the sabbath, even on the sabbath I was making fire. But then on the sabbath you don't make fire, nothing is done, I was even smoking on the sabbath. And she would laugh and be happy when they tell her I no longer go to…
VN: sorry she gave you what, a hiah?
Participant: a hiah
VN: what is a hiah?
Participant: something to dress with, she said her man said, she should give it to me
VN: oh, so this hiah caused you to not go to church anymore?
Participant: yes, to not go to church, I didn't go anymore
VN: so, she put something in it?
Participant: I don't know but it depowered me and ceased my strength.
VN: when did she give it to you?
Participant: she gave it to me while I went to church. Because I had even gone to [name], it's the 20… was it [year] when we went to church with [name]? [year] yeah
VN: oh.
Participant: unclear. On Sunday I went and went again. Since she gave me, I don't even look at the church's door
VN: because of this hiah?
Participant: yes, I also told her that this hiah you gave me
VN: okay so when you think, because you at least still wake in the morning and carry on with life, what keeps you going in life?
Participant: the thing that keeps me going?
VN: yes
Participant: its thinking
VN: thinking of what?
Participant: thinking that my life must go on and shine, it shouldn't be stopped by all these things.
VN: what gives you that hope?
Participant: what gives me what?
VN: that enables you to carry on
Participant: it's the strength I have
VN: oh, you have strength within you, that you get from you?
Participant: aha
VN: there are no other things that help you to carry on?
Participant: no. such as what? Medication?
VN: I don't know.
Participant: no, I don't use any medication. Because I even discontinued ARVs
VN: oh

Participant: yeah
VN: okay
Participant: I only used them during the time when I was protecting the baby when it was still small, after that I stopped
VN: hmm.
Participant: I don't know. Whether it's them, Dr. [name] said maybe they confuse me. So, I stopped them
VN: oh okay. So, the person who has gone through all these things that you've gone through, in what ways do you think they can be assisted?
Participant: me?
VN: I'd like to know how you think you should've been assisted after all that happened to you. What do you wish should happen?
Participant: they said I should have been raised up that's what holding my things back
VN: you should have what?
Participant: I should've been raised up
VN: what do you mean raised up?
Participant: yes, to do Mhlonyana for me. It is holding my things back. I went and told my father, and his wife said we must tell our men to come and pay dowry, so we'd have Memulo. But she did it for her child, as it is her child is working. She even had a stroke she became a teacher. But since she did Mhlonyana she became fine and is working there at [hospital].
VN: oh
Participant: she doesn't want to do our things for us.
VN: and your mother?
Participant: my mother passed away.
VN: on which year?
Participant: [year]
VN: what happened to her?
Participant: she got ill for a short while, then she passed on
VN: oh okay. So, the thing is caught up by not being raised?
Participant: yes
VN: okay. If it could happen
Participant: I think everything could be alright, all fastened things would open. Because when I go to people, they lie to me and not tell me that I was not raised. Others tell me that I was born clothed, all my children were born clothed and all those things.
VN: what is to clothe?
Participant: being born with a cover I don't know what they say is covering. But maybe you know that thing. They say you have luck when you have that thing, but that luck doesn't exist because my mother was Caesar, she didn't see anything, and I was Caesar I didn't see anything.
VN: hmm
Participant: but then I usually see a baby comes out white as if I was eating white sand. I wasn't eating it, so I don't know what causes it.
VN: so, you have luck but there's this thing.
Participant: yes

VN: okay. Isn't there another way that it could be done?
Participant: it could be done. They said my children's father should cleanse the Magubane home and pay so that I'll move out and go there. My luck will come through when I'm not there anymore, when I'm not calling that surname.
VN: [name] is a surname from where?
Participant: it's the one I'm using from my mother's side.
VN: oh, you're using your father's?
Participant: I could use even my father's it's not a problem or the one from the father of my children's side he can pay and take me, and I'd move out and use his surname.
VN: okay is there anything else you'd like to tell me?
Participant: like what sister?
VN: anything
Participant: no.
VN: about experiences. Okay uh so.
